# A small protein coded within the mitochondrial canonical gene *nd4* regulates mitochondrial bioenergetics

**DOI:** 10.1186/s12915-023-01609-y

**Published:** 2023-05-18

**Authors:** Laura Kienzle, Stefano Bettinazzi, Thierry Choquette, Marie Brunet, Hajar Hosseini Khorami, Jean-François Jacques, Mathilde Moreau, Xavier Roucou, Christian R. Landry, Annie Angers, Sophie Breton

**Affiliations:** 1 Département de sciences biologiques, Université de Montréal, Montréal, Canada; 2 Service de génétique médicale, Département de pédiatrie, Université de Sherbrooke, Sherbrooke, Canada; 3 Centre de recherche du Centre hospitalier universitaire de Sherbrooke (CRCHUS), Sherbrooke, Canada; 4 Département de biochimie et génomique fonctionnelle, Université de Sherbrooke, Sherbrooke, Canada; 5 Département de biochimie, de microbiologie et de bio-informatique, Faculté des sciences et de génie, Université Laval, Québec, Canada; 6 Institut de biologie intégrative et des systèmes, Université Laval, Québec, Canada; 7 PROTEO, Le regroupement québécois de recherche sur la fonction, l’ingénierie et les applications des protéines, Université Laval, Québec, Canada; 8 Centre de recherche sur les données massives, Université Laval, Québec, Canada; 9 Département de biologie, Faculté des sciences et de génie, Université Laval, Québec, Canada

**Keywords:** Mitochondria, Mitogenome, Alternative open reading frame, Mitochondrial proteome, Protein coding potential, *nd4*, Mitochondrial bioenergetics, *Homo sapiens*

## Abstract

**Background:**

Mitochondria have a central role in cellular functions, aging, and in certain diseases. They possess their own genome, a vestige of their bacterial ancestor. Over the course of evolution, most of the genes of the ancestor have been lost or transferred to the nucleus. In humans, the mtDNA is a very small circular molecule with a functional repertoire limited to only 37 genes. Its extremely compact nature with genes arranged one after the other and separated by short non-coding regions suggests that there is little room for evolutionary novelties. This is radically different from bacterial genomes, which are also circular but much larger, and in which we can find genes inside other genes. These sequences, different from the reference coding sequences, are called alternatives open reading frames or altORFs, and they are involved in key biological functions. However, whether altORFs exist in mitochondrial protein-coding genes or elsewhere in the human mitogenome has not been fully addressed.

**Results:**

We found a downstream alternative ATG initiation codon in the + 3 reading frame of the human mitochondrial *nd4* gene. This newly characterized altORF encodes a 99-amino-acid-long polypeptide, MTALTND4, which is conserved in primates. Our custom antibody, but not the pre-immune serum, was able to immunoprecipitate MTALTND4 from HeLa cell lysates, confirming the existence of an endogenous MTALTND4 peptide. The protein is localized in mitochondria and cytoplasm and is also found in the plasma, and it impacts cell and mitochondrial physiology.

**Conclusions:**

Many human mitochondrial translated ORFs might have so far gone unnoticed. By ignoring mtaltORFs, we have underestimated the coding potential of the mitogenome. Alternative mitochondrial peptides such as MTALTND4 may offer a new framework for the investigation of mitochondrial functions and diseases.

**Supplementary Information:**

The online version contains supplementary material available at 10.1186/s12915-023-01609-y.

## Background

Mitochondria, energy powerhouses of eukaryotic cells, arose from a symbiotic relationship between a bacterium and a primitive cell. Over approximately two billion years, most of the genes in the “primitive mitochondria” have been lost or transferred to the nuclear genome of their host cells. The nucleus has specialized in coding the anatomical elements of the cell, while the mitochondrion has specialized in coding the key elements of energy production [1, 2]. The human mitogenome (mtDNA) is a small circular DNA with a functional repertoire limited to 37 described genes, i.e., 13 genes encoding key proteins involved in energy production, as well as 2 ribosomal RNAs and 22 transfer RNAs [3]. Almost all these genes are found on the same coding strand, the complementary strand being mostly non-coding [3]. This architecture is radically different from that described for bacterial genomes (which are also circular, but much larger) that derive from the same ancestor as the mtDNA. For example, open reading frames (ORFs) antisense to reference genes can be found in bacteria (i.e., ORFs on the complementary sequence of annotated genes that are transcribed and translated) [4, 5], as well as ORFs within genes (i.e., ORFs nested in annotated genes but in alternative [sense] reading frames that are transcribed and translated) [6, 7]. These sequences, different from the reference coding sequences, are called alternatives (alternative ORFs or altORFs), and they are likely involved in important biological functions, as is the case for DNA sequences encoding very small proteins (small ORFs or smORFs) [8].

The identification of thousands of unannotated altORFs and smORFs as a new class of important genes was revealed more recently for nuclear genomes of eukaryotes including humans [9–15]. Although the elucidation of their function remains challenging, peptides derived from altORFs and smORFs reveal a new facet of eukaryotic proteomes, enabling a better understanding of key evolutionary processes such as the birth of novel genes and proteins, and providing novel fundamental biological knowledge as well as new avenues for therapeutic discovery. Mitochondrial genomes also encode similarly small proteins [16–19].

For example, the 16S and 12S rRNA genes encode 16–38-amino-acid-long micropeptides of functional importance [16–18], and another has been found over serine tRNA (and overlapping tRNA-Leu and *nd5*) [19]. These small proteins, called Humanin, SHLPs (Small Humanin-Like Peptides), MOTS-c, and SHMOOSE appear to modulate mitochondrial and cellular biology [18], as well as global physiology, acting as signaling molecules secreted from cells and found in circulation [17, 20]. However, whether altORFs and smORFs exist in mitochondrial protein-coding genes or elsewhere in the human mitogenome has not been fully addressed.

Mitochondrial DNA mutations are responsible for a variety of human disorders due to defects in energy metabolism, and they are also involved in aging and multiple types of cancer [2, 21, 22]. The diversity of pathologies caused by mtDNA mutations is remarkable and the relationship between genotype and disease phenotype is not always straightforward. For example, mutations within reference genes sometimes cannot be directly linked to effects on energy metabolism or mitochondrial protein synthesis. Breton [23] proposed the mitochondrial Russian doll genes hypothesis to explain some of these discrepancies between theoretical expectation and experimental observation when studying mitochondrial diseases linked to mtDNA mutations. Indeed, molecular screening of mitochondrial disorders has been usually restricted to common harmful mutations (“non-silent” mutations) and deletions of mtDNA, without taking into account rare or silent mutations in protein-coding genes [24]. However, a silent mutation in a reference gene could be non-silent in a mitochondrial altORF (mtaltORF). In other words, the existence of an alternative mitoproteome could well explain some of the discrepancies discussed here, and therefore, this possibility clearly requires attention and a systematic analysis. This is the aim of the present study, i.e., identify and validate mitochondrial altORFs to highlight the existence of a mitochondria-derived alternative proteome and explore the role of the validated mtaltORFs in cellular and mitochondrial physiology.

## Results and discussion

**Fig. 1:**
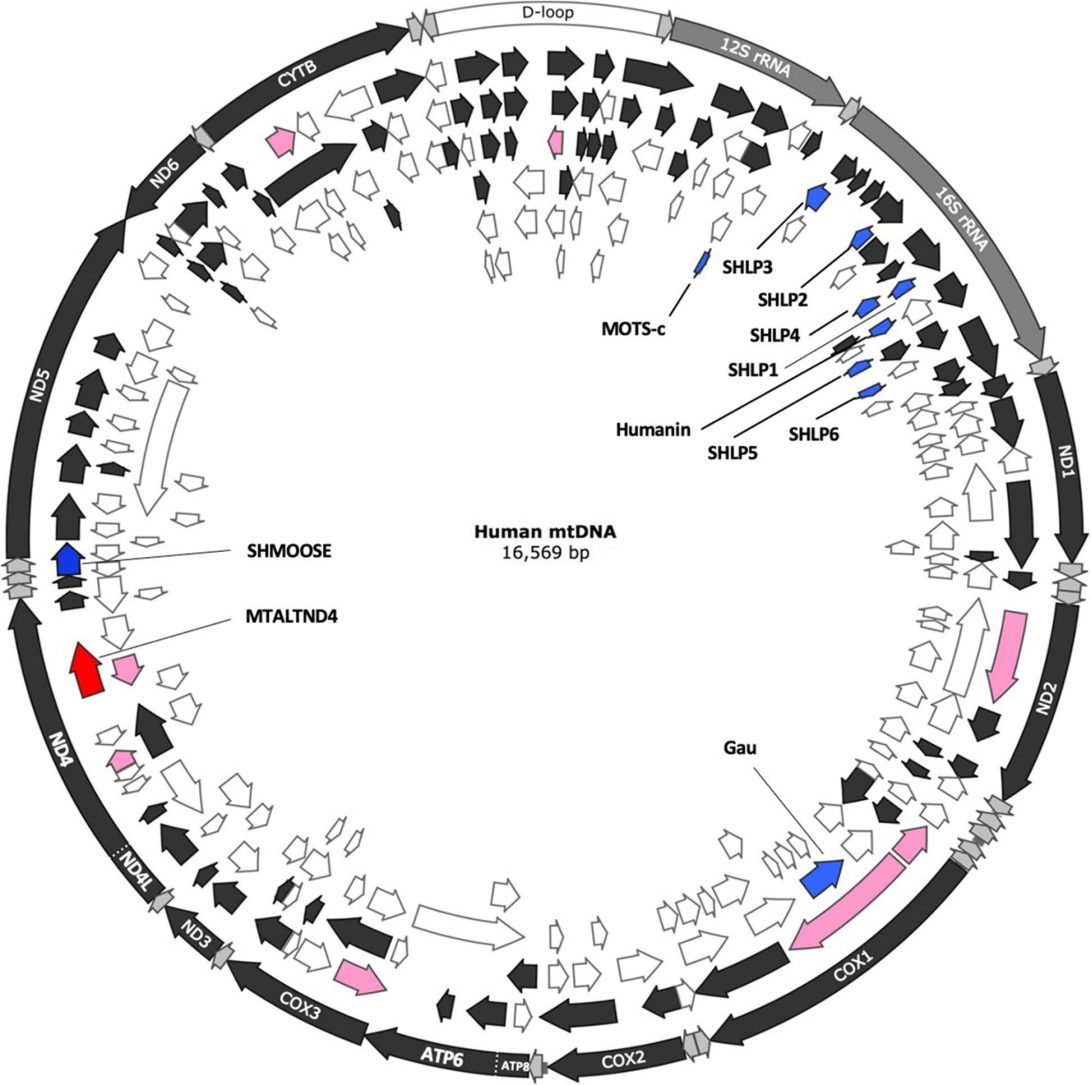
Small ORFs and alternative ORFs in the human mitochondrial genome. In dark gray in the external circle: the typical 13 protein-coding genes. In gray in the external circle: the two rRNA genes. In white in the external circle: the D-loop. Arrows positioned inside the circle indicate smORFs and altORFs on the main coding 5′–3′ (frames 1, 2, and 3: black arrows) and complementary 3′–5′ (frames − 1, − 2, and − 3: white arrows) strands. In blue: the ORFs coding for the micropeptides Humanin, SHLP1-6, and MOTs-c found in the 16S and 12S rRNA genes and SHMOOSE found in the tRNA-Ser overlapping with a small portion of *nad5*. The putative gau gene is also shown. In pink: the eight chosen candidates for antibody production. In red: MTALTND4

An in silico examination of the unresolved protein-coding potential of the human mtDNA revealed 227 unannotated ORFs of at least 60 nucleotides on either strand [25] (Fig. 1, Additional file 1: Table S1). To identify strong candidates for antibody production, unannotated ORFs were selected for further investigation using two different approaches: (i) we interrogated the OpenProt database (Additional file 1: Table S2), which implements a polycistronic model of eukaryotic nuclear and mitochondrial genome annotations [26], and a mass-spectrometry data set (CPTAC_Breast_Cancer, which is extremely rich in MS/MS spectra [~ 32 millions]), to see if peptides derived from our 227 unannotated ORFs could be detected (Additional file 1: Table S3, Additional file 2: Fig. S1). These two approaches respectively returned 14 and 36 candidates (with two overlaps) and we respectively chose 4 and 4 of them for antibody production (Fig. 1, Table 1). The 4 candidates from OpenProt were within protein-coding genes (i.e., “sense frameshifts” in *nd2*, *cox1*, *cytb*, and *nd4*) whereas one from the MS dataset was within a protein-coding gene (*nd4*) and the others were “antisense” to *cox3*, *nd4*, and *cox1* (Fig. 1, Table 1).

**Table 1:** Chosen candidates for antibody production

Approach used	Amino acid sequence (antigenic sequence in bold underlined)	Length	Predicted MW	Position in the reference human mtDNA
OpenProt IP_306387	IFYLSRPRNKHASFYSSSNQKNKPSFHRSCHQVFPHASNRIHNPSNSYPLQQYTLRTMNHNQYYQSMLIINNHNSYSNKTRNSPLSL LSPRGYPRHPSDIRPASSH **MTKTSPHLNHMPNLSLTKRKPSPHSLN**LIHHSRQLRWIKPNPATQNLSMLLNYPHRMNNSSSTVQP^a^	172aa	20 kDa^b^	Within *nd2* sense, frameshift 4547 to 5063
OpenProt IP_306389	ICNNLLHSNTHHNRRLWQLTSSPNNRCPRYGVSPHKQHKLLTLTSLSPTPARICYSGGRSRNRLNSLPSLSRELLPPWSLRRPNHLLL TPSRCLLYLRGHQFHHNNYQYKTPCHNP**MPNAPLRLIRPNHSSPTSPISPSPSCWHHYTTN**RPQPQHHLLRPRRRRRPHSMPTPILI FRSPWSLYSYPTRLRNNLPYCNLLLRKKRTIWMHRYGLSYDINWLPRVYRVSTPYIYSRNRRRHTSMFHLRYHNHRYPHRRQSI^a^	259aa	31.2 kDa^d^	Within *cox1* sense, frameshift 6089 to 6866
OpenProt IP_306398	**MSSSKPHLSPPWLSSPDEATSQNAWTQAHTSYSTP**^a^	35aa	3.8 kDa^c^	Within *nd4* sense, frameshift 11,115 to 11,219
OpenProt IP_306403	**MAESSATFTPMAPQYSLSASSYTSGEAYITDHFSTQKPETSALSSCLQL** ^a^	49aa	5.2 kDa^c^	Within *cytb* sense, frameshift 14,970 to 15,116
MS/MS	MGLSRIEGLFGQVVCGGLGMCFLVLHRAIIGMWLVCWLVGLVWGALWSG**SEITWLGRRSLGGLRGP**LLGVMGWVLLYDRHVIGGSLCVVVQVEAY	95aa	10,3kDa^c^	*cox3* anti-sense 9462 to 9177
MS/MS	MWSLPR**RLPGWPSSARMRRLRAVPRTPAHAPNNRY**SVPMSLWFVENSQRSANISGGEVKWLSEALDCKSKDRG	73aa	8,3kDa^c^	*cox1* anti-sense 6061 to 5842
MS/MS	IMRMTAPVKLQGVWMRMAVTTRAMWLIEEYAMSDFRSVCRRQ**MELVMIMPHRDSTRKG**	57aa	6,9kDa^b^	*nd4* anti-sense 11,709 to 11,535
MS/MS	MRHNYNKLHLPTTNRPKIAHCMLFNQPHS**PRSNSHSHPNPLKLHRRSHSHNRPR**AYILITILPSKLKLRTHSQSHHNPLSRTSNSTPTNSFLMTSSKPR	99aa	11,5kDa^e^	Within *nd4* sense, frameshift 11,557 to 11,854

^a^The sequence is presented with AGA and AGG codons translated to arginine, and the sequence in OpenProt is underlined (OpenProt considers AGA and AGG as stop codons for the mitogenome)

^b^Undetected by western blot

^c^Multiple bands > 40 kDa detected by western blot

^d^Western blot signal of expected weight, but that did not disappear in Rho0 cells devoid of endogenous mtDNA (ethidium bromide-treated HeLa-ρ0 obtained from Abcam [ab154479]), which renders improbable its mitochondrial origin

^e^MTALND4

Of these 8 candidates, one unannotated ORF-derived peptide, which was identified by mass spectrometry (Table 1, Additional file 1: Tables S3), was detected by western blotting in both HeLa and HEK-293 T cells. It is referred to as MTALTND4 (mitochondrial alternative ND4 protein) because its ORF sequences is found in the *nd4* gene in an alternative reading frame. We were unable to obtain western blot signals for the other candidates, or we detected multiple bands > 40 kDa for expected signals < 10 kDa, or we obtained one signal about the expected weight, but it did not disappear in Rho0 cells devoid of endogenous mtDNA (ethidium Bromide-treated HeLa-ρ0 obtained from Abcam [ab154479]), which renders improbable its mitochondrial origin (Table 1).

**Fig. 2:**
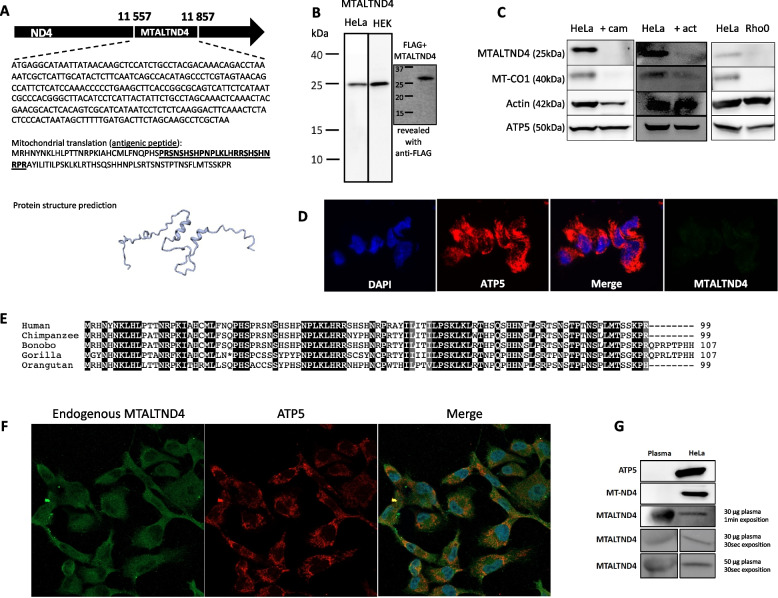
Identification of an alternative protein in the mtDNA-encoded *nd4* gene. **A** The MTALTND4 ORF and its DNA and protein sequences (antigenic sequence underlined), as well as its protein structure prediction. This ORF is frameshifted by 2 nucleotides relative to ND4 reading frame. **B** MTALTND4 detected by western blotting in HeLa and HEK-293 T cells—a protein lysate of HeLa cells transfected with the coding sequence of the corresponding MTALTND4 protein with a FLAG tag was used as a positive control for MTALTND4. **C** MTALTND4, mtDNA-encoded cytochrome c oxidase subunit I (CO1), nuclear-encoded Actin and nuclear-encoded mitochondrial ATP5 in HeLa (control) and HeLa cells treated with chloramphenicol (cam) or actinonin (act), and in HeLa Rho0 cells. **D** Detection of MTALTND4 and nuclear-encoded mitochondrial ATP5 by immunofluorescence in HeLa cells treated with chloramphenicol. **E** Multiple peptide sequence alignment in five primate species. Black background for residues conserved in all species and gray for residues conserved in 4 out of 5 species. Asterisk indicates a stop codon. **F** Detection of MTALTND4 and nuclear-encoded mitochondrial ATP5 by immunofluorescence in HeLa cells. **G** Detection of MTALTND4 and nuclear-encoded mitochondrial ATP5 by western blotting in human plasma

*Mtaltnd4* consists of 300 base pairs that translates into a 99-amino-acid peptide using the mitochondrial vertebrate genetic code and considering that human mitochondria use only UAA and UAG as stop codons as recently suggested [27, 28] (Fig. 2A, Additional file 3: Supplementary text 1) [16, 17, 20, 27–30]. The signal detected for MTALTND4 (~ 24 kDa) was somewhat higher than expected (~12 kDa), but confirmed with our positive control, i.e., a protein lysate from HeLa cells transfected with the coding sequence of the corresponding MTALTND4 protein and a n-terminal FLAG (Fig. 2B, Additional file 2: Fig. S3A), suggesting that FLAG-MTALTND4 in HeLa cells probably formed dimers that could survive boiling 5 min in 4% SDS. The possibility that dimerization (or post-translational modifications) could be responsible for our results was checked (Additional file 2: Fig. S2, Fig. S3B-D), and we showed that purified synthetic MTALTND4 of 12 kDa assembles into homodimers of 24 kDa (Additional file 2: Fig. S3D) and forms higher molecular complexes suggesting that MTALTND4 can self-associate to form homomultimers (Additional file 3: Supplementary text 2) [31–35]. The specificity of the antibody was also checked by adding the synthetic antigenic peptide to the primary antibody solution before the incubation to competitively chelate every antigenic site and show attenuation of the specific bands (Additional file 2: Fig. S3B). The mitochondrial origin of the peptide was proven with the loss of western blot signal in cells treated with actinonin and chloramphenicol that respectively inhibit mitochondrial transcription and protein synthesis, and with the loss of signal in Rho0 cells devoid of endogenous mtDNA (Fig. 2C), limiting the possibility of a nuclear origin due to a nuclear mtDNA transfer (NUMT) [36] (Additional file 2: Fig. S4). The absence of immunofluorescent signal in HeLa-chloramphenicol treated cells confirmed again the mitochondrial origin of the alternative peptide (Fig. 2D). Multiple peptide sequence alignments suggest that MTALTND4 is well conserved in primates (Fig. 2E, Additional file 2: Fig. S5, Additional file 3: Supplementary text 3) [16, 37–42]. The peptide does not possess any predicted transmembrane segment.

We assessed MTALTND4’s subcellular localization and presence in plasma. Similar to Humanin, MOTS-c, and SHLPs [16–18], our results showed that endogenous MTALTND4 is localized in the cytoplasm where it is co-localized to mitochondria (Fig. 2F), and it is detected in plasma, which was not the case for the mitochondrial protein ATP5 (encoded by the nuclear DNA and also devoid of predicted transmembrane helices [TMH]) (Fig. 2G). It is worth noting that the localization of MTALTND4 using a deep learning-based tool [43] is mainly predicted to be extracellular (probability 0.82) or cytoplasmic (0.53). Importantly, our custom antibody, but not the pre-immune serum, was able to immunoprecipitate MTALTND4 from HeLa cell lysates (two different IPs with 2 unique peptide counts and 5 total spectrum counts; Additional file 1: Table S4, Additional file 2: Fig. S6), confirming the existence of an endogenous MTALTND4 peptide (Additional file 3: Supplementary text 4) [27, 44–60].

Following the detection of MTALTND4 in the plasma, we hypothesized that, as other mtDNA-encoded micropeptides [16–18], it could act as a signaling molecule secreted from cells in the circulation and regulate cell physiology. Indeed, emerging studies show that Humanin, SHLPs, and MOTS-c constitute a whole new class of bioactive peptides from the secretome [16–18, 20], although the intracellular mechanisms regulating their production and secretion are still relatively unknown. For example, Humanin increases mitochondrial respiration, cell proliferation, and cell survival through cell membrane receptors [18]. MOTS-c, which appears to exert its function in the cytoplasm and nucleus, decreases mitochondrial respiration, increases glycolysis, and activates transcriptional stress response through its translocation to the nucleus and interaction with transcription factors [16, 20]. Of the six SHLPs, the most studied SHLP2 and SHLP3 have been shown to increase mitochondrial respiration and ATP levels while reducing reactive oxygen species [17]. However, this was observed through an incubation of cells with synthetic SHLP2 and SHLP3, i.e., by testing their exogenous effects on mitochondrial physiology [17]. It thus remains to be established if these micropeptides exert their or some of their functions in the mitochondrial compartment.

**Fig. 3:**
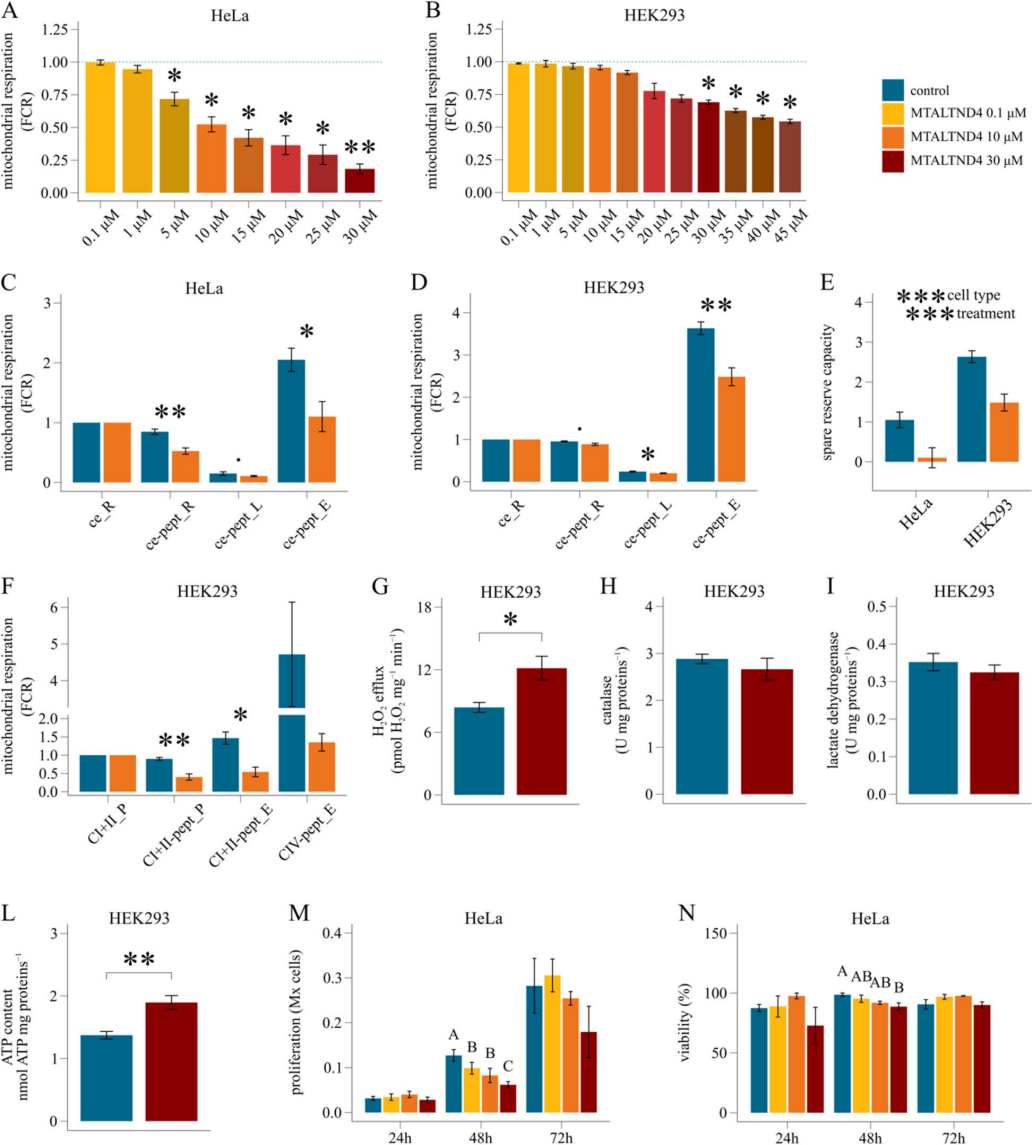
Impact of MTALTND4 upon mitochondrial and cell physiology. **A**–**B** Dose-dependent effect on intact (ce) HeLa and HEK-293 T cells routine respiration (*n* = 3 and *n* = 4, respectively). Respirometry data were normalized for the routine respiration in the absence of the peptide. For each titration point, data are represented as fraction of their own simultaneous control (blue dotted line). **C**–**D** Mitochondrial respiration in intact (ce) HeLa and HEK-293 T cells in the presence of 10 µM MTALTND4 (*n* = 6–9 and *n* = 6, respectively). Respirometry data were expressed as flux control ratios (FCR), normalized for the routine respiration before treatment (*ce_R*). *ce-pept_R* routine respiration with MTALTND4, *ce-pept_L* leak respiration with MTALTND4, *ce-pept_E* maximal uncoupled respiration with MTALTND4. **E** Mitochondrial spare reserve capacity (SRC) in HeLa and HEK-293 T cells in the presence of 10 µM MTALTND4 (*n* = 6 and *n* = 6, respectively). **F** Mitochondrial respiration in permeabilized (pce) HEK-293 T cells in the presence of 10 µM MTALTND4 (*n* = 6). Respirometry data were normalized for the CI + II-sustained coupled respiration before treatment (*CI* + *II_P*). *CI* + *II-pept_P* CI + II-linked coupled respiration with MTALTND4, *CI* + *II-pept_E* CI + II-linked uncoupled respiration with MTALTND4, *CIV-pept_E* cytochrome *c* oxidase standalone capacity with MTALTND4. **G** Hydrogen peroxide efflux rate (pmol H_2_O_2_ ∙ mg proteins^−1^ ∙ min^−1^) in intact HEK-293 T cells in the presence of 30 µM MTALTND4 (*n* = 5). **H** Catalase activity (U ∙ mg proteins^−1^) in intact HEK-293 T cells incubated 4 h with 30 µM MTALTND4 (*n* = 5). **I** Lactate dehydrogenase activity (U ∙ mg proteins^−1^) in intact HEK-293 T cells incubated 4 h with 30 µM MTALTND4 (*n* = 5). **L** ATP content (nmol ATP ∙ mg proteins.^−1^) in intact HEK-293 T cells incubated 4 h with 30 µM MTALTND4 (*n* = 5). **M**–**N** Proliferation (Mx cells) and viability (%) of HeLa cells measured at different time points (24, 48, and 72 h) and different MTALTND4 concentrations (0, 0.1, 10, and 30 µM) (*n* = 3–5). Statistical analyses: **A**–**B** one sample *t* test, **C**, **D**, **F**, **G**, **H**, **I**, **L** paired *t* test, **E** linear mixed model. Factors *cell type* (2 levels) and *treatment* (2 levels) plus interaction. **M**, **N** linear mixed model. Factor *treatment* (4 levels), with letters indicating statistical difference following a post hoc multi comparison test. Data shown as mean ± sem. 0.05 > *p* ≤ 0.09; *p ≤ 0.05; **p ≤ 0.01; ***p ≤ 0.001. A detailed summary (individual values) is reported in Additional file 1: Tables S6-S10

To explore the role of MTALTND4, we used a similar approach than Cobb et al. [17] for the study of the mitochondria-derived micropeptides SHLPs and assessed its exogenous actions by performing cell-based assays to verify if it modulates cell and mitochondrial functions. The effect of MTALTND4 on cellular metabolism was examined using intact HeLa and HEK-293 T cells treated with the complete synthetic peptide at different concentrations. Overall, we observed a strong, immediate effect of MTALTND4 on mitochondrial respiration. Specifically, the presence of the peptide decreased oxygen consumption in both HeLa and HEK-293 T cells. The effect appeared to be dose-dependent, although the concentration threshold differed between cell lines. For example, MTALTND4 at a concentration of 5 µM was sufficient to impact the basal oxygen consumption rate (i.e., the ROUTINE respiration) of HeLa cells, whereas up to 30 µM was necessary in the case of HEK-293 T cells (Fig. 3A–B, Additional file 1: Table S6). Such effect was not observed using the shorter antigenic peptide as a control. It is worth mentioning that a concentration of 30 µM did not negatively impact HeLa or HEK cell proliferation or viability after 24 h treatment (see below).

Additional evidence of the exogenous impact of MTALTND4 comes from analyzing mitochondrial respiration in different respiratory states (Fig. 3C–D, Additional file 1: Table S6). In HeLa cells, 10 µM MTALTND4 significantly decreased both the basal respiration (routine respiration, ce-pept_R) and the maximal uncoupled respiration (ce-pept_E), but not the residual leak respiration (ce-pept_L) (Fig. 3C). In HEK-293 T cells, both leak respiration and maximal uncoupled respiration, but not the routine respiration, were impacted in the presence of 10 µM MTALTND4 (Fig. 3D). This is in line with the dose-dependent effect of the peptide and the higher threshold revealed for HEK-293 T compared to HeLa cells (Fig. 3A–B). The analysis of the “spare respiratory capacity”—i.e., the difference between the basal respiration and the maximal uncoupled respiration (E − R) [61] (and reference therein)—revealed a main effect of both factors “treatment” (control vs peptide addition) and “cell type” (HeLa vs HEK-293 T), with no interaction between the two (Fig. 3E, Additional file 1: Table S7). In accordance with the general notion that cancer cells generally show lower spare respiratory capacity levels compared to their normal counterparts [61], our results suggest that HeLa cells have a physiological respiration much closer to their maximum capacity, thus a lower mitochondrial reserve compared to HEK-293 T cells. A smaller reserve capacity likely makes these cells more sensitive to MTALTND4 treatment, as a lower decrease in maximal respiration is needed to impact the physiological routine respiration. In other words, HeLa cells were less able to withstand the inhibitory effect of MTALTND4 because when E − R reaches zero, mitochondrial energy production fails to meet the minimal needs of the cell.

The impact of MTALTND4 upon mitochondrial respiratory properties does not require cells to be intact. We examined MTALTND4 endogenous effect in permeabilized HEK-293 T cells and observed that 10 µM MTALTND4 impacted both coupled (CI + II-pept_P) and uncoupled maximal respiration (CI + II-pept_E) sustained by CI + II-linked substrates. Although not significant, a sharp decreasing trend in O_2_ consumption was also revealed when testing cytochrome *c* oxidase standalone capacity (CIV-pept_E) (Fig. 3F, Additional file 1: Table S8). This result is of particular interest and deserves further investigations, as it may indicate that the site of action for MTALTND4 within the electron transport system (ETS) could reside in its final oxidase.

A change in reactive oxygen species (ROS—hydrogen peroxide) homeostasis was also observed. Intact HEK-293 T cells in the presence of 30 µM MTALTND4 showed an immediate, steady increase in H_2_O_2_ efflux but no increase in the total H_2_O_2_ scavenging capacity measured after 4 h of incubation (Fig. 3G–H, Additional file 1: Table S9). An increased antioxidant capacity would be expected to lower cell oxidative stress. However, an alternative role for the revealed increased ROS efflux is also possible. Rather than being just deleterious by-products of an impaired mitochondrial respiration, mitochondrial ROS are pivotal mediators for many physiological processes including mitochondrial biogenesis, cell proliferation and differentiation, aging, apoptosis, and response to hypoxia [62]. Given its impact on mitochondrial activity (potentially targeting CIV itself) and ROS metabolism, the potential role of MTALTND4 in cell adaptive response to stress such as hypoxia surely need further attention.

The reduced mitochondrial respiration was not accompanied by a parallel increase in anaerobic glycolysis to partially fulfill the cell energy demand. Four hours of exogenous treatment with MTALTND4 (30 µM) had no impact on the activity of lactate dehydrogenase in HEK-293 T cells (Fig. 3I, Additional file 1: Table S9). Interestingly, despite a reduced mitochondrial capacity and no-change in lactic fermentation, the total ATP content of treated cells increased (Fig. 3L, Additional file 1: Table S9). These results are potentially in line with a general cell metabolism undergoing downregulation, i.e., involving a coordinate downregulation of ATP-producing and ATP-demanding processes. This is further supported by the analyses of the effect of MTALTND4 (0.1, 10, and 30 µM) upon HeLa or HEK-293 T cell proliferation and viability at three different time points (24, 48, and 72 h). A dose-dependent impact of the peptide upon cell proliferation was revealed at 48 h, with a trend still perceivable at 72 h although no more significant (Fig. 3M, Additional file 1: Table S10). The impact of the peptide upon cell viability was less pronounced, with no significant impact after 72 h of treatment (blue trypan) (Fig. 3N, Additional file 1: Table S10), or with a small, but significant negative impact mainly for HeLa cells after 48 h (alamarBlue) (Additional file 2: Fig. S7).

To summarize, we identified a novel human mitochondrial alternative peptide of 99 amino acids (i.e., longer than ATP8 [68 a.a.] and ND4L [98 a.a.]) whose sequence resides inside the *nd4* gene. MTALTND4, which is relatively well conserved in other primate species, is translated inside mitochondria and is also found in the plasma, and it appears to modulate cell and mitochondrial functions. Specifically, MTALTND4 impacted mitochondrial respiration in intact and permeabilized human cells (HeLa and HEK-293 T), decreasing the routine oxygen consumption rate, maximum coupled and uncoupled respiration, as well as the spare reserve capacity. These results were associated with an increase in cellular ATP content and ROS efflux. To our knowledge, none of the previously characterized mitochondria-derived micropeptides induced a similar effect [16–19]. For example, Cobb et al. [17] reported an increase in basal oxygen consumption rate (OCR) caused by SHLPs 2 & 3, which was associated with an increase in cellular ATP and a decrease in ROS production, suggesting that these SHLPs enhance mitochondrial metabolism and have a cytoprotective role. Similar results were obtained upon cell exogenous stimulation with the mitochondria-derived micropeptide Humanin [63].

It is well established that each mitochondrial respiratory complex can be implicated as primary targets of respiratory reserve capacity regulation [61], and our results with permeabilized cells pointed to complex IV as a good candidate. However, it is also well established that spare reserve capacity depends on multiple mitochondrial parameters (e.g., integrity of respiratory complexes and supercomplexes, proton permeability, availability of mitochondrial substrates and TCA activity, mitochondrial homeostasis) and that spare reserve capacity variations render it impossible to identify a specific regulation mechanism [61], although spare reserve capacity determination does provide a synthetic view of the bioenergetic metabolism.

To better understand the biological functions of MTALTND4, we used two complementary approaches, co-immunoprecipitation and pulldown assays, to determine its interacting partners. Immunoprecipitation from HeLa cell extracts allowed to detect MTALTND4 by mass spectrometry and to identify other proteins that could be potential interacting partners of MTALTND4 (Additional file 1: Table S4). One of these proteins, the complement component 1q subcomponent binding protein (C1qbp), was also identified as an interacting partner using pull-down assays with both HeLa and HEK-293 T cell lysates (Additional file 1: Table S5). This interaction was validated by Western blot, i.e., we analyzed the pull-down eluates from GST-MTALTND4 and GST-alone by western blotting using anti-C1qbp antibodies and showed that C1qbp (~ 31 kDa) was present in the input lysates used in both the GST-MTALTN4 and GST-alone pull-down assays and also in the GST-MTALTND4 pull-down, but it was absent in the GST-alone eluate (Additional file 2: Fig. S8). C1qbp, also known as p32, gC1qR, or HABP1, is a multifunctional and multicompartmental protein mostly localized in the mitochondrial matrix but also present at the cell surface, cytosol, and nucleus [64, 65]. C1qbp is an evolutionarily conserved protein that can interact with a diverse array of mitochondrial and cellular, plasma, and microbial proteins [65]. C1qbp regulates cellular energy metabolism through modulation of mitochondrial translation or through protein–protein interactions [66, 67]. It has also a regulatory role in ROS production and can protect cells against oxidative stress [68]. This interaction could potentially explain the observed MTALTND4-mediated changes in mitochondrial respiration and ROS production. The lack of potential compensatory mechanisms accompanying the MTALTND4-mediated increase in H_2_O_2_ efflux and downregulation of mitochondrial respiration such as increased antioxidant activity and upregulation of anaerobic glycolysis, together with the no/little impact on cell viability, suggest that the effects of MTALND4 might not be the result of a deleterious impairment of mitochondrial functions. This bioenergetic depression does however link with a sharp decrease in cell proliferation and increased total ATP content, suggesting that the cells might enter a sort of quiescent state.

## Conclusions

Our findings have several implications. First, they suggest that mitochondrial genes in humans might have gone unnoticed. This calls into question the evolution of the mitochondrial genome as well as the selection pressures exerted on the mitochondria and the mechanisms allowing the translation of these alternative proteins. Our findings also offer a new framework for the investigation of mitochondrial diseases as the existence of an alternative mitochondrial proteome could well explain some of the discrepancies observed previously between mitochondrial genotype and disease phenotype [23]. Indeed, some mutations found in patients with mitochondrial diseases are overlooked because of their synonymous effect on the reference protein [24]. Yet, they could exert a deleterious effect causing non-silent mutations in the altORF protein [23, 24], as shown previously for nuclear genes [69]. In sum, not only does this study enhance our understanding of mitochondrial biology, but it also provides a new mechanism through which mtDNA mutations might generally affect health. Collectively, we anticipate that more studies on the alternative mitochondrial proteome will expedite the discovery of new mitochondrial genes with key biological roles.

## Methods

### Identification of mitochondrial smORFs and altORFs and choice of candidates for antibody production

First, an in silico search was performed for ORFs on both strands encoding peptides > 20 amino acids using the vertebrate mitochondrial genetic code. However, we considered AGA and AGG as arginine codons as it was recently suggested that they are not normally used as stop codons in human mitochondria [27, 28] (Additional file 3: Supplementary text 1) [16, 17, 20, 27–30]. This analysis returned 249 sequences encoding 20- to ≈600-amino-acid-long peptides (the human mtDNA reference sequence GenBank accession number NC_012920 was used; see [25]). We specifically detected 227 unannotated ORFs + the 13 typical protein-coding genes + *Humanin*, *SHLPs* 1–6, *MOTS-c*, and *gau* [70]) (Additional file 1: Table S1).

Two different approaches were then used to choose ORF-derived peptide sequences against which antibodies would be generated. In the first approach, we interrogated the OpenProt database [26]. OpenProt is the first database that enforces a polycistronic model of eukaryotic genome annotations. Specifically, OpenProt retrieves transcripts from two standard annotations (Ensembl and NCBI RefSeq), which constitutes an exhaustive transcriptome. A three-frame in silico translation then yields the ORFeome: any ORF longer than 30 codons and starting with an ATG in any frame of any transcript. This ORFeome is then filtered to categorize predicted ORFs. The first filter retrieves all known protein (all ORF already annotated in Ensembl, NCBI RefSeq, and/or UniProtKB), these are the RefProts. The second filter looks at the homology of the currently not annotated ORFs with the RefProt of the same gene (if applicable) and retrieves novel predicted isoforms. The remaining ORFs encode novel proteins, called AltProts [26]. For mitochondrial transcripts, OpenProt uses the vertebrate mitochondrial genetic code (but with AGA and AGR considered as stop codons). To increase confidence in ORF expression, OpenProt also cumulates several lines of evidence, such as (i) conservation evidence (for every ORF annotated, OpenProt identifies orthologs and paralogs across the 10 species currently supported by OpenProt), (ii) translation evidence (OpenProt retrieves publicly available ribosome profiling datasets and re-analyze them using the Price algorithm with a stringent 1% FDR), and (iii) expression evidence (OpenProt retrieves publicly available mass spectrometry datasets and re-analyze them using multiple search engines, and a stringent 0.001% FDR) [26]. The search in OpenProt returned 14 altORFs encoding 29–61-amino-acid-long peptides, eight of them were found within the 12S and 16S ribosomal genes and six in protein-coding genes (Additional file 1: Table S2). We choose to focus on these latter six, which were all conserved in *Pan troglodytes* except for one altORF. Moreover, these six altORFs generated very few hits > 70% similarity (i.e., less than 4) when searched against the nuclear genome (data not shown). Four of these altORFs were used for antibody production (Table 1, Additional file 1: Table S2).

In the second approach, we interrogated the CPTAC_Breast_Cancer mass spectrometry dataset to see if peptide sequences resulting from our first approach could be detected. Specifically, we used the novel tool PepQuery to interrogate spectra from the published CPTAC_Breast_Cancer dataset. This approach compares spectrum annotation with peptides from our novel sequences to annotation with any protein from the reference database (known proteins). We chose the CPTAC_Breast_Cancer dataset as it is one of the largest in terms of MS/MS scans with more than 32 million. We first identified all possible peptides from our 249 proteins (Additional file 1: Table S1). We performed an in silico Trypsin digestion, allowing up to 2 miscleavages, a minimum peptide length of 7 amino acids, and a mass from 400 to 6000 Da to mimic experimental conditions. Then, each peptide was queried using the PepQuery tool (Additional file 2: Fig. S1).

From the PepQuery tool [71], we retrieved any peptide spectrum match (PSM) that is better matched with our queried peptide than with any protein from the reference database. Various filters were used to validate the results as confident or not. For a PSM to be considered as confident, a *p-*value under 0.01 and a score higher than any peptide carrying any post-translational modification (PTM) from the reference database were needed. Using these criteria, we could be confident a given spectrum corresponds to our queried peptide and not to a reference peptide with any PTM [71]. As such, Table S3 (Additional file 1) contains for each of the 249 predicted proteins (Additional file 1: Table S1) the number of PSMs with higher score than a referenced peptide, the number of confident PSMs, the best confident PSM (score), its corresponding *p-*value, and its total mass. This analysis returned 46 protein sequences with confident detection by mass spectrometry (including the 13 reference genes). Antibodies have been generated against 4 of the putative proteins (Table 1, Additional file 1: Table S3).

### Analyses of mtaltnd4 sequences

To test for the presence of nuclear mtDNA sequences (NUMTs) identical or very similar to *mtaltnd4* due to recent mitochondrial transfer [36], we used the NCBI nucleotide basic local alignment search tool (BLASTN) [72] against the human genome reference (Additional file 2: Fig. S4). The degree of conservation of MTALTND4 was assessed by aligning the protein sequences from primate and mammal species obtained from the NCBI database. TBLASTn searches were used to ensure correct extraction of the protein sequences, which were aligned using ClustalW Multiple Alignment [73] (Fig. 2, Additional file 2: Fig. S5). Putative transmembrane (TM) helices were searched using TMPred [74], but none was found. Putative post-translational modifications (PTM) of MTALTND4 were searched using some of the PTM prediction webservers recently reviewed by Audagnotto and Dal Peraro [75], only scores with 80% probabilities were retained (Additional file 2: Fig. S2). RoseTTAFold was used to predict the protein structure of MTALTND4 (Fig. 2A) [76].

### Rabbit anti-MTALTND4 antibody generation and MTALTND4 peptide synthesis

The custom rabbit anti-MTALTND4 was ordered from MediMabs (Montreal, QC, Canada) (and also the other custom antibodies; Fig. 1, Table 1). High-titer polyclonal anti-sera against MTALTND4 was obtained. The complete MTALND4 peptide was synthesized, using Fmoc and Boc chemistry, microwave technology, and a proprietary solid support resin, by LifeTein (Somerset, NJ, USA). The purity of the peptide was verified by HPLC by the supplier. MTALTND4 (M.W. 11,532.06 gr∙mol^−1^) was shipped lyophilized to the University of Montreal and resuspended to a final stock concentration of 4 mM in ultrapure H_2_O following the manufacturer instructions. A working solution of 0.4 mM was prepared, and aliquots stored at − 80 °C for further experiments.

### Cell culture, HeLa rho0 cells, actinonin, and chloramphenicol treatments

HeLa and HEK-293 T cells were cultured in Dulbecco’s modified Eagle medium (DMEM) supplemented with 10% calf bovine serum, penicillin, streptomycin, and fungizone and were kept at 37 °C with 5% CO_2_.

Actinonin treatment was realized on HeLa cellular cultures. They were split into 2 groups, the first serving as control and was thus left untouched, while the second was incubated with actinonin (150 µM). Both treatments were then left to incubate for 48 h [77]. Chloramphenicol-treated HeLa cells were produced by adding 2 ml of chloramphenicol diluted at 50 µg·ml^−1^ in 95% ethanol in a 10-cm petri dish of 80% cell confluency. Cells were treated for 48 h before being harvested for western blot analysis [78]. Ethidium Bromide-treated HeLa-ρ0 cell lysates (vs. control) were obtained from Abcam, Toronto, Canada (ab154479).

### Western blotting

HeLa or HEK-293 T cells were washed in PBS, harvested, and lysed with a sonic dismembrator sonicator (Fisher, Ottawa, Canada) in a homogenizer buffer with a pH of 7.4 containing 10 mM HEPES, 150 mM NaCl, and a cocktail of protease inhibitors (benzamidine, PMSF, aprotinin, and leupeptin). Triton X-100 was added to 10% final concentration after sonication. Total protein concentration was estimated using the Bradford dosage method. The samples were then mixed with a Laemmli buffer containing DTT and ß-mercaptoethanol reducing agents and heated at 95 °C for 5 min before being loaded at equal protein concentration (100 µg) on Tricine-SDS-PAGE gels. SDS-PAGE gels were composed of three parts (separating, spacer, and compaction gels) [79]. To maximize the separation of low molecular weight proteins, the migration was first done for an hour at low voltage (30 V). Then the voltage was increased to 43 V, and the migration carried out overnight at room temperature. For the transfer, PVDF membranes of 0.2 µm were used which allows better retention of small proteins. The transfer was done for 1 h at 1000 mA at 4 °C in a transfer buffer containing 194 mM glycine, 25 mM Tris-base, and 20% methanol in H_2_0. Before incubation with the primary antibodies, membranes were blocked for 30 min at room temperature in a blocking buffer containing 5% milk powder and 0.05% tween-20 diluted in PBS. Primary antibodies rabbit anti-MTALTND4 (1:1000), mouse anti-ATP5 (1:1000; Abcam [ab14748]), mouse anti-MTCO1 (1:500; Abcam [ab110270]), and mouse anti-actin (1:2000; Abcam [ab14705]) were diluted in a PBS + 0.05% tween-20 solution. Membranes were incubated with the primary antibodies for 2 h at room temperature. After incubation, membranes were washed 3 × 5 min in TBS-T at room temperature. Secondary antibodies goat anti-rabbit IgG (1:2000) and 46 goat anti-mouse IgG (1:2000) coupled to the horseradish peroxidase (HRP) were diluted in a PBS + 0.05% tween-20 solution. Membranes were incubated with secondary antibodies for 30 min at room temperature. Finally, membranes were washed 3 × 10 min with TBS-T and 1 × 5 min with TBS at room temperature. Protein signals were visualized by adding Montreal Biotech Inc.’s enhancer and substrate solutions and images were captured with a FUSION FX chemiluminescence imaging system. A Flag-MTALTND4 plasmid construct that includes a n-terminal FLAG sequence (DYKDDDDK), a GS linker (GGGGSGGGGSGGGGS), and MTALTND4 (only the ORF) was used as a positive control (Fig. 2B). The full sequence was generated by Bio Basic Gene Synthesis service (Markham, ON, Canada) and subcloned into a pcDNA 3.1- plasmid (XhoI/EcoRI). HeLa cells were transfected with jetPRIME® (CA89129-924, VWR, Toronto, Canada) according to the manufacturer’s protocol. Twenty-four hours later, cells were washed in PBS and scratched in RIPA lysis buffer (1% Triton X-100, 1% NaDeoxycholate, 0.1% SDS, 1 mM EDTA, 50 mM Tris–HCl pH 7.5). Protein concentration was measured using the BCA protein assay reagent (Pierce, Waltham, MA, USA). The samples were then mixed with a Laemmli buffer and heated at 95 °C for 5 min before being loaded (130 μg) on a 15% SDS-PAGE gel. After migration, proteins were transferred on PVDF 0.45 µm membrane. Anti-Flag antibody (F1804, Sigma, 1:4000) and anti-mouse HRP antibody (sc-516102, Santa Cruz, 1:8000) were used for the revelation.

To verify if predicted post-translational modifications (Additional file 2: Fig. S2), i.e., glycosylation and phosphorylation, were responsible for the observed MTALTND4 band (~ 24 kDa) at higher MW than expected (11.5 kDa), whole cell lysates were treated with a complete protein deglycosylation mix II kit (NEB, Ipswich, MA, USA; Cat# P6044) following the manufacturer’s protocol, as well as with a lambda protein phosphatase (NEB, P0753S) following the manufacturer’s protocol (Additional file 2: Fig. S3). To verify the specificity of the anti-MTALTND4 antibody, the synthetic antigenic peptide was added to the primary antibody solution at a 10 × concentration before the incubation, to competitively chelate every antigenic site of the primary antibody and to show the attenuation of the specific MTALTND4 band (Additional file 2: Fig. S3). To verify if a SDS-resistant dimer could be responsible for the observed MTALTND4 band at higher MW than expected [80], we exposed our samples to different concentrations of ß-mercaptoethanol (0%, 5%, and 10%) and different heating times (5 min, 30 min and 3 h) (Additional file 2: Fig. S3). The synthetic MTALTND4 peptide was also subjected to chemical cross-linking to verify if it could self-associate to form a dimer. Specifically, 3 µg of synthetic MTALTND4 peptide was incubated at 30 °C for 10 min in 20 µl of PBS 1 × containing 0%, 0.5%, or 1% paraformaldehyde. The cross-linking reaction was stopped by the addition of LSB 1 × and the resulting products were separated on a two-part 16.5% and 5% tricine-SDS-PAGE gel. The gel was stained with Coomassie Imperial Protein Stain. The presence of MTALTND4 in the plasma was verified by western blotting (blood-derived human plasma containing K2-EDTA anticoagulant was obtained from Innovative Research [IPLASK2E2ML]).

### Immunofluorescence

HeLa cells were cultured on an 8-well PCA detachable microscope slide in DMEM supplemented with 10% calf bovine serum, fungizone, penicillin, and streptomycin and were kept at 37 °C with 5% CO_2_. Each chamber was washed with PBS and fixed with 4% paraformaldehyde for 20 min. Cells were then washed 3 × 5 min in PBS before their membranes were permeabilized by adding 0.2% Triton for 4 min. Cells were washed 3 × 5 min with PBS and then blocked with 5% NGS in PBS for 30 min at room temperature before incubating for 1 h with primary antibodies rabbit anti-MTALTND4 antibody (1:50) and mouse anti-ATP5 antibody (1:250; Abcam) diluted in 1% NGS in PBS. Cells were washed 3 × 5 min in PBS before incubating with the secondary antibodies (Alexa Fluor ™ 488 goat anti-rabbit IgG [1:500] and Alexa Fluor ™ 594 goat anti-mouse IgG [1:500]) in PBS containing 1% NGS for 1 h at room temperature. Finally, cells were washed 3 × 5 min in PBS before adding a mounting drop containing DAPI and visualized with an EVOS M5000 microscope (Fig. 2D) or Zeiss-LSM800 (Fig. 2F).

### Immunoprecipitation and mass spectrometry analysis

HeLa cells (*n* = 3 replicates) were washed with PBS and harvested in a lysing buffer containing 10 mM HEPES, 150 mM NaCl, and a cocktail of protease inhibitors (benzamidine, PMSF, aprotinin, and leupeptin). Cells were then lysed with a sonic dismembrator sonicator (Fisher) 3 times for 30 s before adding 100 µl of Triton X-100 10%. Cell lysates were incubated on ice for 20 min and then centrifuged for 15 min at 15,000 RPM at 4 °C. The supernatant was kept; 20 µl of protein A agarose was added to 1 ml of samples and incubated at 4 °C for 1 h. Samples were then centrifuged at 15,000 rpm for 1 min at 4 °C and the supernatant was kept; 20 µl of rabbit anti-MTALTND4 antibody and 20 µl of protein A agarose were added to the samples before incubating at 4 °C overnight, and 20 µl of rabbit pre-immune serum was used as control. Samples were washed 6 times with lysing buffer. Samples were then sent to the proteomics platform of the McGill University Health Center (MUHC) Research Institute for mass spectrometry analysis. Samples were loaded onto a single stacking gel band to remove lipids, detergents, and salts. The single gel band containing all proteins was reduced with DTT, alkylated with iodoacetic acid, and digested with trypsin; 2 µg of extracted peptides were re-solubilized in 0.1% aqueous formic acid and loaded onto a Thermo Acclaim Pepmap (Thermo, 75 µM ID × 2 cm C18 3 µM beads) precolumn and then onto an Acclaim Pepmap Easyspray (Thermo, 75 µM × 15 cm with 2 µM C18 beads) analytical column separation using a Dionex Ultimate 3000 uHPLC at 250 nl·min^−1^ with a gradient of 2–35% organic (0.1% formic acid in acetonitrile) over 3 h. Peptides were analyzed using a Thermo Orbitrap Fusion mass spectrometer operating at 120,000 resolutions (FWHM in MS1) with HCD sequencing (15,000 resolution) at top speed for all peptides with a charge of 2 + or greater. The raw data were converted into *.mgf format (Mascot generic format) for searching using the Mascot 2.6.2 search engine (Matrix Science) against Human Uniprot sequences (2022). The database search results were loaded onto Scaffold Q + Scaffold_4.9.0 (Proteome Sciences) for statistical treatment and data visualization (Additional file 1: Table S4).

### Pull-down assay and mass spectrometry analysis

Transformation of competent cells and protein expression were performed following manufacturer’s instructions (Thermo Fisher Scientific, Waltham, MA, USA; BL21(DE3) Competent Cells EC0114). For the transformation, 1 µl of GST-tagged MTALTND4 expression plasmid (LifeTein, Somerset, NJ, USA) was added to 50 µl of BL21(D3) competent cells originating from an *E. coli* B strain. A GST expression plasmid was used as control. Cells were incubated on ice for 30 min, then heat-shocked for 30 s in a 42 °C water bath before returning on ice for 2 min; 250 µl of LB broth growth medium was then added, and the tube was placed in a shaking incubator at 225 rpm for 1 h at 37 °C. The transformed cells were then spread on previously prepared LB plates containing 25 µl of ampicillin and incubated at 37 °C overnight. For protein expression, a transformant colony was picked and inoculated in 5 ml of LB medium with 5 µl ampicillin. The culture was incubated overnight with shaking at 37 °C, allowing it to grow to saturation; 200 µl of overnight culture was added to 10 ml of fresh LB medium containing 10 µl of antibiotic. The culture was incubated for 2 h, 10 µl of IPTG was then added, and it was incubated for an additional 2 h. The induced culture was centrifuged at 2500 rpm for 10 min and the pellet was resuspended in 2 ml of PBS 1 × with 4 M urea. A cocktail of protease inhibitors was added, and the pellet was lysed as described above for the HeLa cells.

For protein extraction, the bacterial lysates were incubated overnight with 100 µl of a 50% slurry of glutathione sepharose beads (Abcam) at 4 °C for pre-coupling. The beads were washed 3 times with PBS 1 × and the protein quantity assessed via a Bradford protein assay. HeLa or HEK-293 T cell lysates were then prepared as described above and pre-cleared using 50 µl of glutathione sepharose beads and 25 µg of GST protein (Sino Biological, Beijing, China) for 2 h at 4 °C with end-over-end mixing. The lysates were then centrifuged for 2 min at 15,000 rpm at 4 °C and the supernatant was collected. From the pre-coupled MTALTND4 protein, 10 µg was added to 1 ml of the lysates and incubated overnight at 4 °C. Samples were washed 6 times with lysing buffer. Samples (two replicates for HeLa cells and one sample for HEK-293 T cells) were then sent to the proteomics platform of the McGill University Health Center (MUHC) Research Institute for mass spectrometry analysis. Samples were treated and analyzed as above for immunoprecipitations (Additional file 1: Table S5).

The validation of a predicted interaction with the complement component 1q subcomponent binding protein (C1qbp) was done via Western blot as described above (primary antibody mouse anti-GC1q (1:1000; Abcam [ab24733]), was diluted in a PBS + 0.05% tween-20 solution, and secondary antibody goat anti-mouse IgG (1:2000) coupled to the horseradish peroxidase (HRP) was diluted in a PBS + 0.05% tween-20 solution) (Additional file 2: Fig. S8).

### Cell proliferation and viability

HeLa or HEK-293 T cells were treated with complete synthetic MTALTND4 (0.1, 10, or 30 μM) or water (control) in DMEM low glucose buffer for 24, 48, and 72 h. Cell proliferation (*n* = 3) was evaluated directly by counting manually with a hemocytometer. To test for cell viability (*n* = 3), 10 μL of 4% trypan blue exclusion dye were added to 10 μL of treated or untreated HeLa cells and examined under a microscope. Numbers of viable cells were estimated using a hemocytometer. Cell viability (*n* = 5) was also determined by alamarBlue assay kit (Themo Fisher; DAL1025) following the manufacturer’s protocol.

### High resolution respirometry (HRR)

Cells (~ 80% confluency) were trypsinized (2.5%), washed, centrifuged, and finally resuspended in MiR05 mitochondrial respiratory buffer (110 mM D-sucrose, 60 mM lactobionic acid, 20 mM taurine, 20 mM HEPES, 10 mM KH_2_PO_4_, 3 mM MgCl_2_, 0.5 mM EGTA, BSA 1 g∙L^−1^). MiR05 was supplemented with 5 mM pyruvate (MiR05 + P) as external energy source to support intact cell respiration [51]. Cell viability (%) and concentration (Mx cells∙mL^−1^) were determined through a Neubauer hemocytometer and trypan blue staining.

Mitochondrial respiration has been characterized by high resolution respirometry (HRR), using a dedicated Oxygraph-2 k with DatLab software v7.4 (Oroboros Instruments, Innsbruck, Austria). The respiratory chambers were preloaded with 2.1 mL MiR05 + P respiratory medium and calibrated for oxygen saturation at 37 °C. Collected cells were transferred into each respiratory chamber by replacing part of the chamber media, reaching 2.5 mL total chamber volume and cell concentration of 0.5–0.8 Mx cells∙mL^−1^, depending on the cell line and the specific experiment [81, 82]. Cell suspensions were continuously stirred at 750 rpm and part of the solution (400 µL) was removed, sonicated (5 times 1 s, on ice), and stored at − 80 °C for protein determination. Chambers (final volume 2.1 mL) were then sealed, and oxygen consumption monitored. Data were corrected for instrumental background O_2_ flux measured at 37 °C in a separate experiment [81, 82]. Mitochondrial respiration was investigated in both intact (ce) and permeabilized (pce) cells.

#### HRR in intact cells

Oxygen consumption in intact HeLa and HEK-293 T cells has been monitored following sequential titration of MTALTND4 peptide. After recording the respiration of intact cells (ROUTINE, R-state) in the presence of 5 mM pyruvate (ce_R), increasing peptide concentrations were tested. This included 0.1 µM, 1 µM, 5 µM, 10 µM, 15 µM, 20 µM, 25 µM, and 30 µM for *n* = 4 HeLa, and 0.1 µM, 1 µM, 5 µM, 10 µM, 15 µM, 20 µM, 25 µM, 30 µM, 35 µM, 40 µM, and 45 µM for *n* = 3 HEK-293 T. Simultaneous controls were treated with an equal volume of H_2_O titrated at the same time points. Respirometry data were normalized for an internal parameter, the ROUTINE (R) respiration—i.e., the respiration rate of intact cells before treatment—and expressed as flux control ratios (FCR). For each titration point, data are expressed as fraction of the control respiration measured in parallel at the same time. The exogenous impact of peptide addition was also assessed on intact HeLa (*n* = 6–9) and HEK-293 T (*n* = 6) cells following a specific coupling control protocol (CCP). After the measurement of ROUTINE respiration in the presence of 5 mM pyruvate (ce_R), the exogenous impact of MTALTND4 was assessed at a final concentration of 10 µM (ce_pept_R). Addition of the ATP-synthase inhibitor oligomycin (15 nM) declined the respiration to the level of leak respiration (L, leak state—state 4 or 2’) and allowed the measurement of the residual respiration due to the futile proton cycle (ce_pept_L). The maximal respiration was then stimulated by the titration of FCCP (0.125 µM each step) (ce_pept_E). Finally, the residual oxygen consumption (ROX) was determined in the presence of 2.5 µM antimycin A (complex III inhibitor) and subtracted from the data. The “spare respiratory capacity” (SRC) was obtained for both HeLa and HEK-293 T cells by subtracting routine respiration from the maximal uncoupled respiration (E − R) (*n* = 6–6). Data were normalized for the ROUTINE respiration and expressed as flux control ratios.

#### HRR in permeabilized cells

HEK-293 T cells (*n* = 6) were added intact to each chamber and ROUTINE respiration (ce_R) was determined in presence of complex I (CI) substrates pyruvate (5 mM), malate (2 mM), and glutamate (10 mM). The plasma membrane of cells was then selectively permeabilized with 7.5 µg∙mL^−1^ digitonin, and leak respiration (leak, L-state—state 4 or 2’) in the presence of CI-linked substrates measured (CI_L). The optimum digitonin concentration for complete plasma membrane permeabilization of cultured cells was determined empirically following existing protocols [81–84]. Addition of 2.5 mM ADP stimulated mitochondrial respiration (OXPHOS, P-state or state 3) sustained by CI substrates (CI_P). To verify the intactness of mitochondrial membranes, cytochrome *c* (10 µM) was added to the chambers (c_P). Addition of complex II (CII) linked substrate succinate (10 mM) fueled the OXPHOS activity, now sustained by both CI and CII substrates (CI + II_P). This represents the maximal coupled respiration and data presented as flux control ratios have been normalized for it. Graphical representations of the data start from this point onward. The impact of MTALTND4 was tested against the CI + II sustained coupled respiration, at a final peptide concentration of 10 µM (CI + II-pept_P). Further addition of FCCP (titration, 0.125 µM each step) stimulated the maximal uncoupled respiration (ETS, E-state or state 3u) (CI + II-pept_E). Residual oxygen consumption (ROX) was achieved in the presence of 0.5 µM rotenone (Rot—complex I inhibitor) and 2.5 µM antimycin A (complex III inhibitor), and subtracted from the data. Cytochrome *c* oxidase (complex IV, CIV) standalone activity was measured in the presence of 2 mM ascorbate and 0.5 mM TMPD (CIV-pept_E). The residual oxygen consumption due to autoxidation was determined with 20 mM sodium azide (complex IV inhibitor) and subtracted from CIV activity.

### Hydrogen peroxide efflux

The rate of hydrogen peroxide (H_2_O_2_) efflux from intact cells was determined using the Amplex® Red Hydrogen Peroxide Assay Kit (Invitrogen, Waltham, MA, USA; A22188). HEK-293 T cells (*n* = 5) were collected and suspended in the respiratory buffer MiR05 supplemented with 5 mM pyruvate at a concentration of 5 Mx cells∙mL^−1^. Each sample was split in two (control and treatment). Before the treatment, homogeneous aliquots were collected, lysed through sonication (5 times 1 s strokes on ice) and kept frozen at − 80 °C for further protein determination. Intact cells were incubated with either 30 µM MTALTND4 (*n* = 5) or the same volume of H_2_O for the paired controls (*n* = 5) for 30 min at 37 °C. After peptide exposition, samples were transferred to a black microplate and incubated in the dark for 30 min at 37 °C in the presence of 0.01 mM Amplex® Red, 0.1 U∙mL^−1^ horseradish peroxidase (HRP), and 50 mM sodium phosphate buffer, pH 7.4. The H_2_O_2_ efflux was then measured following the increase in fluorescence (excitation 560 nm; emission 615 nm) for 1.5 h, using a dedicated Mithras LB940 microplate reader (Berthold technologies, Bad Wildbad, Germany). Hydrogen peroxide efflux rate was determined with the use of a standard curve and normalized for the amount of proteins (pmol H_2_O_2_ ∙ mg proteins^−1^ ∙ min^−1^).

### Enzymatic activities and ATP content

HEK-293 T cells (10 Mx cells∙mL^−1^) were incubated for 4 h at 37 °C in MiR05 supplemented with 5 mM pyruvate and 30 µM MTALTND4 (*n* = 5). Each sample was split and had its own control, with H_2_O used instead of the peptide (*n* = 5). Cells were lysed (5 times 1 s strokes on ice) using a sonic dismembrator (Fisher) and kept frozen at − 80 °C for further analysis of enzymatic activities and ATP content.

The rates of enzymatic activity were measured spectrophotometrically with a Mithras LB940 microplate reader (Berthold technologies, Bad Wildbad, Germany). Enzymatic capacities were expressed as µmol of substrate transformed to product per minute (units—U) over mg of proteins. Enzymatic assays were performed in duplicates at 37 °C in the following conditions.

#### Catalase (CAT, EC 1.11.1.6)

CAT activity was estimated as the total H_2_O_2_ scavenging capacity. It was measured at 240 nm following the disappearance of H_2_O_2_ (*ε*_240_ = 43.6 M^−1^∙cm^−1^) for 1 min. The medium was composed of 50 mM potassium phosphate, 0.1% (*v*/*v*) triton X 100 and 60 mM H_2_O_2_, pH 7.0. The background activity in the absence of sample was subtracted from the main results [85, 86].

#### Lactate dehydrogenase (LDH, EC 1.1.1.27)

LDH activity was determined by the rate of NADH oxidation (*ε*_340_ = 6.22 mM^−1^∙cm^−1^) following pyruvate reduction to lactate. The medium was composed of 50 mM potassium phosphate, 0.16 mM NADH, and 0.4 mM pyruvate, pH 7.0. The specificity of the reaction was tested in the presence of 30 mM oxamate (LDH inhibitor) [87].

#### ATP content

ATP was quantified using the ATP Determination Kit (Sigma, St. Louis, MO, USA; A22066). The bioluminescence assay follows the ATP-dependent light production in a reaction involving a recombinant firefly luciferase and its substrate D-luciferin. The conditions were set as follows: 0.5 mM D-luciferin, 1.25 μg·mL^−1^ firefly luciferase, 1 mM dithiothreitol, 5 mM MgSO_4_, 100 μM EDTA, 100 μM sodium azide, and 25 mM tricine buffer, pH 7.8. The luminescence was measured in duplicates immediately after sample addition, in a luminometer (Mithras LB940, Berthold technologies) set at 28 °C. The content of ATP in the sample was derived using a standard curve with known concentrations of ATP, to which the background luminescence recorded in the absence of sample was subtracted. Data are presented as ATP over total protein content (nmol ATP ∙ mg proteins^−1^).

#### Protein content

The total level of proteins (mg∙ml^−1^) of each sample was determined in duplicate through the Bicinchoninic Acid (BCA) assay (Bicinchoninic Acid Protein Assay Kit, Sigma BCA1) using a Bovine Serum Albumin (BSA) standard curve [88].

### Statistical analyses

High-resolution respirometry data were collected and preliminary analyzed using DatLab v7.4 (Oroboros Instruments), while enzymatic activities, ROS efflux rate, ATP, and protein content with MikroWin 2010 software (Labsis Laborsysteme, Neunkirchen-Seelscheid, Germany). Graphical representation and statistical analyses were performed using the R software [89] and ggplot2 package [90]. The normality and homoscedasticity of data were verified with Shapiro and Levene’s tests, respectively. The dose-dependent impact of peptide addition upon mitochondrial respiration was determined at each titration point using a one sample *t* test, comparing each treatment with its respective parallel control. For all the parameters included in the CCP and SUIT protocols, as well as for enzymatic activities (CAT and LDH), ATP content, and H_2_O_2_ efflux rate, the effect of peptide addition was tested with the means of paired *t* test or Welch test. A linear mixed model was implemented for the parameters SRC, proliferation, and viability. The factors “cell type” and “treatment” (effect of peptide addition) were considered as fixed effects when analysing HeLa and HEK-293 T SRCs. The only factor “treatment” was considered for HEK-293 T cell proliferation and viability. In both cases, cell ID was implemented as random effect (paired samples). The significance of both factors and their possible interaction was determined through a type III ANOVA and post hoc multi comparison. Data are graphically presented as mean ± standard error of the mean (s.e.m). Statistical significance was set at *p* ≤ 0.05 and *p*-values were corrected with Holm adjustment for multiple testing. Detailed summaries are provided in Additional file 1: Tables S6-S10.

## Supplementary Information

**Additional file 1: Table S1.** Identification of mitochondrial smORFs and altORFs - in silico approach. **Table S2.** Identification of mitochondrial smORFs and altORFs - OpenProt approach and sequences selected for antibody production. **Table S3.** Identification of mitochondrial smORFs and altORFs - MS approach and sequences selected for antibody production. **Table S4.** List of proteins found by mass spectrometry following immunoprecipitation. **Table S5.** List of proteins found by mass spectrometry following pull down assay. **Table S6.** Dose-dependent impact of MTALTND4 on routine respiration of intact HeLa and HEK-293T cells. **Table S7.** Impact of 10 µM MTALTND4 on mitochondrial respiration of intact HeLa and HEK-293T cells. **Table S8.** Impact of 10 µM MTALTND4 on mitochondrial respiration of permeabilized HEK-293T cells. **Table S9.** Impact of MTALTND4 on intact HEK-293T cell's lactic fermentation, antioxidant capacity, ATP content and hydrogen peroxide efflux rate.** Table S10.** Dose- and time-dependent impact of MTALTND4 on HeLa cells proliferation and viability.

**Additional file 2: Figure S1.** Using the PepQuery tool to interrogate spectrums from published proteome datasets. **Figure S2.** Putative post-translational modifications of MTALTND4. O-Glyc: putative O-glycosylation; P: putative phosphorylation. PTM prediction were done using webservers reviewed by [75], only scores with 80% probabilities were retained. **Figure S3.** Specificity of the anti-MTALTND4 antibody and detection of putative post-translational modificationsof MTALTND4. A. Complete western blot of the antibody on a cell lysate. B. Specificity of anti-MTALTND4 and PTMs. Deglyc: deglycosylationnon-treated cells,treated cells and fetuin positive control are shown); Dephosphos: dephosphorylationnon-treated cells andtreated cells are shown). C. Apparent molecular weight of MTALTND4 exposed to different concentrations of denaturing agent β-Mercaptoéthanol and different heating times at 95˚C. A = 5 minutes, B = 30 minutes and C = 3 hours. 1 = 0% β-Mercaptoethanol, 2 = 5% β-Mercaptoethanol and 3 = 10% β-Mercaptoethanol. D. Self-association of MTALTND4: 3 µg of synthetic peptide were incubated at 30 °C for 10 min in the presence of 0%, 0.5% and 1% paraformaldehyde, quenched with LSB, separated by tricine-SDS-PAGE and visualized with Coomassie Imperial Protein stain. Arrowheads indicate main cross-linked products. **Figure S4.** Comparison of MTALTND4 peptide encoded in mitochondrial DNAand hypothetically in nuclear DNA that have been transferred from mtDNAthrough evolution. Only sequences with >50% identities and complete without stop codons are shown. **Figure S5.** Multiple MTALTND4 peptide sequence alignments. A. In 15 mammal species: human, chimpanzee, bonobo, gorilla, orangutan, mouse, rat, naked mole rat, dog, cow, zebrafish, lion, bear, horse, dolphin. B. In the genus Homo: Homo heidelbergensis; Homo sapiens neanderthalensis; Homo sapiens neanderthalensis; Homo sapiens neanderthalensis; Homo sapiens neanderthalensis; Homo sapiens neanderthalensis; modern human, chimpanzee. *Indicates a stop codon. **Figure S6.** Endogenous MTALTND4 detected in HeLa cells by mass spectrometry. Unique mass spectrometry MTALTND4-derived peptides detected in HeLa cells lysates. Probabilities = 100%. **Figure S7.** Effect of MTALTND4 on cell viability. HeLa and HEK-293T cells were cultured in low glucose DMEM with 0.1 µM, 10 µM or 30 µM MTALTND4 peptide or waterfor 24h or 48h and assessed for cell viability using the alamarBlue assay. **Figure S8.** GST pull-down assay indicating that MTALTND4 and Complement component 1 Q interact. Western blot probed with anti-C1qbp antibodies. Lane 1: pull-down products from GST bound glutathione beads. Lane 2: pull-down products from GST-MTALTND4–bound glutathione beads. Lane 3: HeLa cells lysate. Lane 4: pull-down products from GST bound glutathione beads. Lane 2: pull-down products from GST-MTALTND4–bound glutathione beads. Lane 3: HEK-293T cells lysate. **Figure S9.** Original uncropped blots for MTALTND4.

**Additional file 3: Supplementary Text 1.** Mitochondrial genetic code. **Supplementary Text 2**. Observed and expected molecular weights on SDS-PAGE. **Supplementary Text 3**. Conservation of MTALTND4. **Supplementary Text 4**. Transcription and translation of MTALTND4.

## Data Availability

All data generated or analyzed during this study are included in this published article, its supplementary information files, and publicly available repositories. The uncropped gels/blots are provided in Additional file 2: Fig. S3, Fig. S9. Bioinformatics data are provided in Additional file 1: Tables S1-S3. Physiological data are provided in Additional file 1: Tables S6-S10. The mass spectrometry data are provided in Additional file 1: Tables S4-S5 (summary) and have been deposited (scaffold files) in Dryad 10.5061/dryad.n02v6wx2p .

## Abbreviations

altORF: Alternative open reading frame
ce-pept_E: Maximal uncoupled respiration of intact cells
ce-pept_L: Residual leak respiration of intact cells
ce-pept_R: Basal or routine respiration of intact cells
CI + II-pept_E: Uncoupled maximal respiration of permeabilized cells sustained by CI+II
CI + II-pept_P: Coupled respiration of permeabilized cells sustained by CI+II
CIV-pept_E: Cytochrome *c* oxidase standalone capacity
MTALTND4: Mitochondrial alternative ND4 protein
mtaltORF: Mitochondrial alternative open reading frame
mtDNA: Mitogenome or mitochondrial DNA
NUMT: Nuclear mtDNA Transfer
ORF: Open reading frame
ROS: Reactive oxygen species
SHLP: Small humanin-like peptide
sORF: Small open reading frame
SRC: Mitochondrial spare reserve capacity
TMH: Transmembrane helix
