# Supplementary material for: A small protein coded within the mitochondrial canonical gene nd4 regulates mitochondrial bioenergetics
Source: BMC Biol. 2023 May 18;21:111. doi: 10.1186/s12915-023-01609-y (PMC10193809; doi:10.1186/s12915-023-01609-y)
Supplement: Supplementary file 1 — Additional file 1: Table S1. Identification of mitochondrial smORFs and altORFs - in silico approach. Table S2. Identification of mitochondrial smORFs and altORFs - OpenProt approach and sequences selected for antibody production. Table S3. Identification of mitochondrial smORFs and altORFs - MS approach and sequences selected for antibody production. Table S4. List of proteins found by mass spectrometry following immunoprecipitation. Table S5. List of proteins found by mass spectrometry following pull down assay. Table S6. Dose-dependent impact of MTALTND4 on routine respiration of intact HeLa and HEK-293T cells. Table S7. Impact of 10 µM MTALTND4 on mitochondrial respiration of intact HeLa and HEK-293T cells. Table S8. Impact of 10 µM MTALTND4 on mitochondrial respiration of permeabilized HEK-293T cells. Table S9. Impact of MTALTND4 on intact HEK-293T cell's lactic fermentation, antioxidant capacity, ATP content and hydrogen peroxide efflux rate. Table S10. Dose- and time-dependent impact of MTALTND4 on HeLa cells proliferation and viability. [file 12915_2023_1609_MOESM1_ESM.pdf]

Supplementary Tables

Table S1. Identification of mitochondrial smORFs and altORFs - in silico approach.

Vertebrate mitochondrial genetic code
Start: ATG, ATT, ATA
Stop: TAA, TAG
AGA, AGG coding

START	STOP	LENGTH	CODON I	CONTEXT	Kozak -3	Kozak +4	CODON F	**FEATURE**	PROTEIN SEQUENCE
49	199	50	ATT	TCTCCATGC ATT G	T	G	TAA		IWYFRLGGMHAMALRDAGAGAPYVAVSVFDSC<br>LILLFIAPTFNITGEHTY
232	334	34	ATA	TTGTAGGAC ATA T	G	T	TAA		MMMTIECLHSHFPHRHHNKKFPPNPPSPASGH ST
448	832	128	ATT	AACTAACAC ATT T	C	T	TAG		IIFPSHSHTTNLINTTPAHPTQHTHTAANPMPRT<br>NQTPKTPPTVYVAYLLKAMHWKCLDGLTSPHKQ<br>MGLVLAFLLALSKITHASIPVPVSSPSKSPRSKGTSI<br>KHAAMQLKTLSLATPPRETAVINL
967	1195	76	ATA	CCCTCCCCA ATA A	C	A	TAG	**RNR1**	MKLKLTWVVKNSSWHKMDYESGFNMSEHTMA<br>KTQTGIRYPTMLSPKPQQLNQQNCSPEHYEPQL<br>KTQRTWRCFMSL
1258	1426	56	ATA	TCAGCCTAT ATA C	T	C	TAG	**RNR1**	MPPSSANPDEGYKVSASTHVKTLGQGVAHEVAR<br>NGLHFLPQKTTMALMKLKGRRWI
1540	1609	23	ATT	CCCCTACGC ATT A	C	A	TAG	**RNR1**	IYMEETSRNMVSVLESALGRTRV
1963	2038	25	ATA	TGTAGCAAA ATA T	A	T	TAG	**RNR2**	MVGRFMGRGDKPTEPGDSWLSKMES
2323	2563	80	ATT	CATGAAAAC ATT T	A	T	TAA	**RNR2**	ILLRMSLRQIKTLNWQLTAQYLQSTNKSLLPSLST<br>QHRHAHKERLKKVKGTRQILPRLFTKNITSSITSIR<br>GTACPVTHV
2617	2800	61	ATA	GTTCCTTAA ATA G	T	G	TAA	**RNR2**	MGTCMNGSTRVQLSLTFNQWNWPAREEAGM<br>TQQDEKTLWSFNLLMQTVPNKPTGPKLPNLH
2893	3232	113	ATT	CTATACTCA ATT A	T	A	TAA	**RNR2**	IDPMTWPTEQVTLGMTAQSYSRVHINNRVYDL<br>DVGSGHPDGAAAIKGSFVQRLKSYVIWVQTGVI<br>QVGFYLXSNSSLYERTREMRPTSQSAFPRKWYHL<br>NLVLYPHPPKNRVC

3289	4261	324	ATT	AGAGGTTCA ATT C	T	C	TAA	**TRNL1**	IPLLNNMPMANLLLLIVPILIAMAFLMLTERKILGY<br>MQLRKGPNVVGPYGLLQPFADAMKLFTKEPLKP<br>ATSTITLYITAPTLALTIALLLWTPLPMPNPLVNLN<br>LGLLFILATSSLAVYSILWSGWASNSNYALIGALRA<br>VAQTISYEVTLAIILLSTLLMSGSFNLSTLITTQEHL<br>WLLLPSWPLAMMWFISTLAETNRTPFDLAEGES<br>ELVSGFNIEYAAGPFALFFMAEYTNIIMMNTLTTT<br>IFLGTTYDALSPELYTTYFVTKTLLLTSLFLWIRTAY<br>PRFRYDQLMHLLWKNFLPLTLALLMWYVSMPITI<br>SSIPPQT
5425	5500	25	ATA	AGTTTGAAC ATA A	A	A	TAA	**ND2**	MQNPPHSSPHSSPLPRYSYLSPLLY
5857	5989	44	ATT	TGTCTTTAG ATT A	T	A	TAG		IYSPMLHSAILPHPHWCSPTVDYSLQTTKTLEHYT<br>YYSAHELES
7540	7738	66	ATA	AACCATTTC ATA C	T	C	TAA	**TRND**	MTLSKLNYRLNPMYLNGTCSASRSTRRYFPYHRR<br>AYHLSWSRPHNHFPYLLPSPVCPFPNTHNKTN
7894	8311	139	ATG	TGGCCACCA ATG T	C	T	TAG	**COX2**	MVLNLRVHRLRRTNLQLLHTSPIIPRTRRPATPW<br>RWQSSSTPDWSPHSYNNYITRRLALMSCPHIRLK<br>NRCNSRTSKPNHFHRYTTGGMLRSMLWNLWSK<br>PQFHAHRPRINSPKNLWNRARIYPMAPPLPPLEP<br>TVKLT
8335	8506	57	ATT	AAGTTAAAG ATT A	A	A	TAA	**TRNK**	IKRTNTSLQWNAPTKYYRMAHHNYPHTPYTIPH<br>HPTKNIKHKLPPTSLTKAHKNKKL
8527	9205	226	ATG	AGAACCAAA ATG A	A	A	TAA	**ATP8**	MNENLFASFIAPTILGLPAAVLIILFPPLLIPTSKYLI<br>NNRLITTQQWLIKLTSKQMMTMHNTKGRTWSL<br>MLVSLIIFIATTNLLGLLPHSFTPTTQLSMNLAMAI<br>PLWAGTVIMGFRSKIKNALAHFLPQGTPTPLIPM<br>LVIIETISLLIQPMALAVRLTANITAGHLLMHLIGSA<br>TLAMSTINLPSTLIIFTILILLTILEIAVALIQAYVFTLL<br>VSLYLHDNT
9721	9802	27	ATT	TGGGTCTCT ATT T	T	T	TAG	**COX3**	ILPSYKPQSTSSLPSPFPTASTAQHFL
9943	10003	20	ATG	ATTTTGTAG ATG G	T	G	TAA	**COX3**	MWFDYFCMSPSIDEGLTLLV
10789	10861	24	ATT	AACAATTAT ATT C	T	C	TAG	**TRNC-comp**	ITTTDMTFQKTHNLNQHNHPQPNY

11086	11410	108	ATT	AATTATAAC ATT A	A	A	TAA	**ND4**	IHSHRTNHILYLLRNHTYPHLGYHHPMRQPARTP<br>ERRHMLPILHPSRLPSPTHRTNLHSQHPRLTKHST<br>THSHCPRTIKLLSQQLNMTSLHNSFYSKDTSLRTP<br>LMTP
11557	11854	99	ATG	ACTATCCCT ATG G	C	G	TAA	**ND4**	MRHNYNKLHLPTTNRPKIAHCMLFNQPHSPRSN<br>SHSHPNPLKLHRRSHSHNRPRAYILITILPSKLKLRT<br>HSQSHHNPLSRTSNSTPTNSFLMTSSKPR
12046	12139	31	ATT	AAAACCCTC ATT A	C	A	TAA	**ND4**	IHTRKHPHVHTPIPHSPPIPQPRHHYRVFLL
12310	14146	612	ATT	GCCCCAAAA ATT T	A	T	TAA	**TRNL2**	ILVQLQMKVMTMHTTMTTLTLTSLIPPILTTLVNP<br>NKKNSYPHYVKSIVASTFIISLFPTTMFMCLDQEVI<br>ISNWHWATTQTTQLSLSFKLDYFSMMFIPVALFV<br>TWSIMEFSLWYMNSDPNINQFFKYLLIFLITMLIL<br>VTANNLFQLFIGWEGVGIMSFLLISWWYARADA<br>NTAAIQAILYNRIGDIGFILALAWFILHSNSWDPQ<br>QMALLNANPSLTPLLGLLLAAAGKSAQLGLHPW<br>LPSAMEGPTPVSALLHSSTMVVAGIFLLIRFHPLA<br>ENSPLIQTLTLCLGAITTLFAAVCALTQNDIKKIVAF<br>STSSQLGLMMVTIGINQPHLAFLHICTHAFFKAM<br>LFMCSGSIIHNLNNEQDIRKMGGLLKTMPLTSTS<br>LTIGSLALAGMPFLTGFYSKDHIIETANMSYTNA<br>WALSITLIATSLTSAYSTRMILLTLTGQPRFPTLTNI<br>NENNPTLLNPIKRLAAGSLFAGFLITNNISPASPFQ<br>TTIPLYLKLTALAVTFLGLLTALDLNYLTNKLKMKS<br>PLCTFYFSNMLGFYPSITHRTIPYLGLLTSQNLPLLL<br>LDLTWLEKLLPKTISQHQISTSIITSTQKGMIKLYFL<br>SFFFPLILTLLLIT
14152	14218	22	ATT	ACATAACCT ATT C	C	C	TAA		IPPSNLNYNMYTNKQCSTSNYY
14230	14386	52	ATA	TCAACGCCC ATA T	C	T	TAA		MIMQSPRTNRILPNQPWPLSFMNYSASYTIKVY<br>HNHHPIMLFHPQHQSYLHR
14524	14584	20	ATA	TAAACCCAT ATA C	C	C	TAA		MTSPKIQNNNTPDHTANNQY
14665	14767	34	ATA	AAACAAAGC ATA A	A	A	TAA		MHHYSRTDYNHDQWYEKPSLYFNYKNTNDPNT<br>QN

14797	15394	199	ATT	TAACCACTC ATT A	C	A	TAA	**CYTB**	IHRPPHPIQHLRMMKLRLTPWRLPDPPNHHRTI<br>PSHALLTRRLNRLFINRPHHSRRKLWLNHPLPSR<br>QWRLNILYLPLPTHRARPMLRIISLLRNLKHRHYP<br>PACNYSNSLHRLCPPVRPNIILRGHSNYKLTIRHP<br>MHWDRPSSMNLRRLLSRQSHPHTILYLSLHLALH<br>YCSPSNTPPPILARNGIKQPPRNHLPFR
15469	15601	44	ATT	CTTAATGAC ATT A	G	A	TAA	**CYTB**	INTILTRPPRRPRQLYPSQPLKHPSPHQARMMFP<br>IRLHNSPIRP
15628	15895	89	ATT	CCTTGCCCT ATT C	C	C	TAG	**CYTB**	ITIHPHPSNNPHPPYIQTTKHNISPTKPITLLTPSRR<br>PPHSNLNRRTTSKLPFYHHWTSSIRTMLHNNPN<br>PNTNYLPNWKQNTQMGLSL
15907	15997	30	ATA	TATAAACTA ATA A	C	A	TAA	**TRNT**	MHQSCKPEMKTFFQGQIREKVFNSTISTQS
16087	16315	76	ATG	ACAACCGCT ATG A	G	A	TAA		MYFVHYCQPPWMLYGTMNTWPPVVHKNPIHI<br>KTPSPCLQASTAINPQLSHINCNSKATPHPLGYQ<br>QTYPPLTVHST
16333	16462	43	ATA	TTACCGTAC ATA C	T	C	TAA		MAHYSQIPSRPHGWPPSDRGPLTTILREINIPHKS<br>ATLLAPGP
116	209	31	ATG	GAGCACCCT ATG C	C	C	TAA		MSQYLSLIPASSYYLSHLRSMLQANMLTKVC
281	383	34	ATA	ACAGACATC ATA C	A	C	TAA		MTKNFHQTPPPPLLATALKHISAKPQKQRTLTPA
470	584	38	ATA	CCCACTCCC ATA T	C	T	TAG		MLLISSMQPPPILPSTHTPLLTPYPEPTKPQRHPP<br>QFM
695	794	33	ATG	AGATTACAC ATG A	C	A	TAG	**RNR1**	MQASPFQWVHPLNHHDQKEQASSTQQCSSKRL<br>A
860	968	36	ATA	AACTAAGCT ATA T	G	T	TAA	**RNR1**	MLTPGLVNFVPATAVTRLTQVNRSRRKECFRSPP<br>PQ
1751	1862	37	ATA	GTATAGGCG ATA A	G	A	TAA	**RNR2**	MEIETWRNRYSTARERWKIMTKHNMARTNPYT<br>FCMMN
1871	1934	21	ATA	TAACTAGAA ATA C	G	C	TAA	**RNR2**	MTLQGEPKLRPPKPDELPKNS
2192	2336	48	ATT	CAGCCACCA ATT A	C	A	TAA	**RNR2**	IKKAFKLNTHYLKNPKHMTELLTPNWTNLSPYRR<br>TNVSMSNMKTFSSA
2633	2705	24	ATG	CCTGTATGA ATG C	T	C	TAA	**RNR2**	MAPRGFSCLLLLTSEIDLPVKRRA
2798	2903	35	ATT	CAAACCTGC ATT A	T	A	TAA	**RNR2**	IKNFGWGDLGAEPNLRAVHAKTSPVKANYYTQLI<br>Q
2951	3050	33	ATT	CGCAATCCT ATT T	C	T	TAA	**RNR2**	ILESMSTMGFTTSMLDQDIPMVQPLLKVRLFND

3236	3347	37	ATG	TTTGTTAAG ATG C	A	C	TAA	**TRNL1**	MAEPGNRMKLKTLQSEVQFLFLTTYPWPTSYSSL<br>YPF
4217	4277	20	ATG	TTATATGAT ATG C	G	C	TAA	**ND1**	MSPYPLQSPAFPLKPKKYVW
4325	4418	31	ATT	AACCCCCTT ATT C	C	C	TAA	**TRNI**	ISRTMRIEPIPENPKFSVPPITPHPKVRSAK
4547	5063	172	ATT	CTCGCACTG ATT T	C	T	TAA	**ND2**	IFYLSRPRNKHASFYSSSNQKNKPSFHRSCHQVFP<br>HASNRIHNPSNSYPLQQYTLRTMNHNQYYQSM<br>LIINNHNSYSNKTRNSPLSLLSPRGYPRHPSDIRPA<br>SSHMTKTSPHLNHMPNLSLTKRKPSPHSLNLIHH<br>SRQLRWIKPNPATQNLSMLLNYPHRMNNSSSTV<br>QP
5114	5291	59	ATT	TACTACCGC ATT C	C	C	TAG	**ND2**	IPTTQLKLQHHDPTTISHLKQANMTNTLNSIHPPL<br>PRRPAPANRLFAQMGHYRRIHKKQ
5411	5519	36	ATG	AAAAATAAA ATG C	A	C	TAG	**ND2**	MTVWTYKTHPIPPHTHRPYHATPTYLPFYTNNL<br>MEI
5573	5642	23	ATT	CAATACTTA ATT C	T	C	TAA	**TRNW**	ISVTAKDCKTPLCINWTQISHFN
5807	6050	81	ATG	CAATTCAAT ATG A	A	A	TAA		MKITSELVKRGLTPVFRFTVQCFTQPFYLTPTDVR<br>RPLTILYKPQRHWNTMPIIRRMSWSPRHSSKPPY<br>SSRAGPARQPSR
6089	6866	259	ATT	AGCCCATGC ATT G	T	G	TAG	**COX1**	ICNNLLHSNTHHNRRLWQLTSSPNNRCPRYGVS<br>PHKQHKLLTLTSLSPTPARICYSGGRSRNRLNSLPS<br>LSRELLPPWSLRRPNHLLLTPSRCLLYLRGHQFHH<br>NNYQYKTPCHNPMPNAPLRLIRPNHSSPTSPISP<br>SPSCWHHYTTNRPQPQHHLLRPRRRRRPHSMP<br>TPILIFRSPWSLYSYPTRLRNNLPYCNLLLRKKRTI<br>WMHRYGLSYDINWLPRVYRVSTPYIYSRNRRRH<br>TSMFHLRYHNHRYPHRRQSI
6902	7442	180	ATG	CAATATGAA ATG T	G	T	TAG	**COX1**	MICCSALSPRIHLSFHRRWPDWHCISKLITRHRTT<br>RHVLRCSPLPLCPINRSCICHHRRLHSLISPILRLHP<br>RPNLRQNPFHYHIHRRKSNFLPTTLSRPIRNAPTL<br>LGLPRCMHHMKHPIICRLIHFSNSSNINNFHDLRS<br>LRFEAKSPNSRRTLHKPGVTMWMPPTLPHIRRT<br>RMHKI

7586	8267	227	ATG	TATATCTTA ATG C	T	C	TAG	**COX2**	MAHAAQVGLQDATSPIMEELITFHDHALMIIFLI CFLVLYALFLTLTTKLTNTNISDAQEMETVWTILP AIILVLIALPSLRILYMTDEVNDPSLTIKSIGHQWY WTYEYTDYGGLIFNSYMLPPLFLEPGDLRLLDVD NRVVLPIEAPIRMMITSQDVLHSWAVPTLGLKTD AIPGRLNQTTFTATRPGVYYGQCSEICGANHSFM PIVLELIPLKIFEMGPVFTL
8366	8570	68	ATG	TACAGTGAA ATG C	G	C	TAG	**ATP8**	MPQLNTTVWPTMITPMLLTLFLITQLKMLNTNY HLPPSPKPMKMKNYNKPWEPKWTKICSLHSLPP QS
9251	9653	134	ATG	ACCCAGCCC ATG C	C	C	TAG	**COX3**	MTPNRGPLSPPNDLRPSHVISLPLHNAPHTRPTN QHTNHMPMMARCNTRKHMPRPPHTTCPKRPS MRDNPIYYLRSFFLRRIFLSLLPLQPSPYPPIRRALA PNRHHPAKSPRSPTPKHIRITRIRSINHLSSP
9794	9998	68	ATT	CGGCTCAAC ATT T	A	T	TAG	**COX3**	IFCSHRLPRTSRHYWLNFPHYLLHPPTNISLYIQTS LWLRSRRLMLAFCRCGLTISVCLHLLMRVLLF
10121	10304	61	ATT	AATTATTAC ATT T	T	T	TAA	**ND3**	ILTTTTQRLHRKIHPLRVRLRPYIPRPRPFLHKILLSS YYLLIIWSRNCPPFTPTMSPTNN
10337	10412	25	ATT	ATCCCTCTT ATT A	C	A	TAG	**ND3**	INHHPSPKSGLWVTTKRIRLNRIGM
10502	10742	80	ATT	TATACTAGC ATT A	A	A	TAA	**ND4L**	IYHLTSRNTSMSLTPHILPTMPRRNNTIAVHYSYS HNPQHPLPLSQYCAYCHTSLCRLRSSGGPSPTSL NLQHMWPRLRT

10760	12278	506	ATG	CCTACTCCA ATG T	C	T	TAA	**TRNC-comp**	MLKLIVPTIMLLPLTWLSKKHMIWINTTTHSLIISII<br>PLLFFNQINNNLFSCSPTFSSDPLTTPLLMLTTWLL<br>PLTIMASQRHLSSEPLSRKKLYLSMLISLQISLIMTF<br>TATELIMFYIFFETTLIPTLAIITRWGNQPERLNAG<br>TYFLFYTLVGSLPLLIALIYTHNTLGSLNILLLTLTAQ<br>ELSNSWANNLMWLAYTMAFMVKMPLYGLHL<br>WLPKAHVEAPIAGSMVLAAVLLKLGGYGMMRL<br>TLILNPLTKHMAYPFLVLSLWGMIMTSSICLRQTD<br>LKSLIAYSSISHMALVVTAILIQTPWSFTGAVILMI<br>AHGLTSSLLFCLANSNYERTHSRIMILSQGLQTLLP<br>LMAFWWLLASLANLALPPTINLLGELSVLVTTFS<br>WSNITLLLTGLNMLVTALYSLYMFTTTQWGSLTH<br>HINNMKPSFTRENTLMFMHLSPILLLSLNPDIITG<br>FSSCKYSLTKTSDCESDNRGLRPLIYRESSQELLTH<br>APMSNNMAFSTFKG
12824	12893	23	ATG	CCCGAGCAG ATG C	C	C	TAG	**ND5**	MPTQQPFKQSYTTVSAMSVSSSP
14171	14231	20	ATT	GCAATCTCA ATT C	T	C	TAA		ITMYTPTNNVQPVTTTNQRP
14297	14498	67	ATT	CCTTCATAA ATT T	T	T	TAA		IIQLPTLLKFTTTTTPSYSFTHSTNPTSIANPTKTLTK<br>TSTPDPHASGYSSMAIAVVYPKTTIIPPK
14597	15905	436	ATA	CCCCCATAA ATA G	T	G	TAA		MGEGLEENPTNPITKPTLNRNKAYIIILARTTTTTN<br>DMKNHRCISTTRTPMTPMRKTNPLMKLINHSFI<br>DLPTPSNISAWWNFGSLLGACLILQITTGLFLAM<br>HYSPDASTAFSSIAHITRDVNYGWIIRYLHANGAS<br>MFFICLFLHIGRGLYYGSFLYSETWNIGIILLLATM<br>ATAFMGYVLPWGQMSFWGATVITNLLSAIPYIG<br>TDLVQWIWGGYSVDSPTLTRFFTFHFILPFIIAALA<br>TLHLLFLHETGSNNPLGITSHSDKITFHPYYTIKDAL<br>GLLLFLLSLMTLTLFSPDLLGDPDNYTLANPLNTPP<br>HIKPEWYFLFAYTILRSVPNKLGGVLALLLSILILAM<br>IPILHMSKQQSMMFRPLSQSLYWLLAADLLILTW<br>IGGQPVSYPFTIIGQVASVLYFTTILILMPTISLIENK<br>MLKWACPCSMN
16016	16136	40	ATT	ATTTAAACT ATT T	A	T	TAA		ILCSFMGKQIWVPPKYWLTHQQPLCISYITASHH<br>EYCTVP

16160	16271	37	ATA	CTGTAGTAC ATA A	T	A	TAG		MKTQSTSKPPPHAYKQVQQSTLNYHTSTATPKP<br>PLTH
16340	16475	45	ATT	ACATAGCAC ATT C	C	C	TAG		ITVKSLLVPMDDPPQMGVPWPPSSVKSMSRTR<br>VLLSSLRAHNTWG
21	213	64	ATT	TATCACCCT ATT A	C	A	TAA		INHSRELSMHLVFSSGGYARDSIARRWSRSTLCRS<br>ICLWFLPHPIIYRTYVQYYRRTYLLKCVN
219	282	21	ATG	AATTAATTA ATG T	T	T	TAA		MLVGHNNNNWMSAQPLSTQTS
291	372	27	ATT	TAACAAAAA ATT C	A	C	TAA		ISTKPPLPRFWPQHLNTSLPNPKNKEP
390	477	29	ATT	CCTAACCAG ATT C	C	C	TAA		ISNFIFWRYALLTVTPQLTHYFPLPLPYY
924	1035	37	ATA	CCCAAGTCA ATA A	T	A	TAA	**RNR1**	MEAGVKSVLDHPLPNKAKTHLSCKKLQLTQNRL<br>RKWL
1218	1407	63	ATA	CTGTAATCG ATA A	T	A	TAA	**RNR1**	MNPDQPHHLLLSLYTAIFSKPWWRLQSKRKYPR<br>KDVRSRCSPWGGKKWATFSTPENYDSPYET
2052	2226	58	ATT	CAACTTTAA ATT G	T	G	TAA	**RNR2**	ICPQNPLNPLVNLTVSPKRNSSLDTRKKPCRESKK<br>FNTHSRPKSSHQLRKRSSSTPTT
2370	2445	25	ATT	GAACTGACA ATT A	A	A	TAA	**RNR2**	INSPMSTINQQVIITLTVNPTQACS
2598	2667	23	ATA	AAAGGTAGC ATA T	A	T	TAA	**RNR2**	MITCSLNRDLYEWLHEGSAVSYF
2805	2955	50	ATT	GCATTAAAA ATT C	A	C	TAG	**RNR2**	ISVGATSEQNPTSEQYMLRLHQSKRTTMLNWSN<br>NLTNGTSYPRDNSAILF
3048	3174	42	ATT	TGTTCAACG ATT A	A	A	TAA	**RNR2**	IKVLRDLSSDRSNPGRFLSXFKFLPVRKDKRNKAY<br>FTKRLPP
3177	3258	27	ATG	CCCCCGTAA ATG T	T	T	TAA	**RNR2**	MMSSQLSIMPTPTQEQGLLRWQSPVIA
3360	3633	91	ATT	CGCAATGGC ATT C	G	C	TAG	**ND1**	IPNAYRTKNSRLYTTTQRPQRCRPLRATTTLRWR<br>HKTLHQRAPKTRHIYHHPLHHRPDLSSHHRSSTM<br>NPPPHTQPPGQPQPRPPIYSSHL
3825	4299	158	ATT	ACACCTCTG ATT C	C	C	TAA	**ND1**	ITPAIMTLGHNMIYLHTSRDQPNPLRPCRRGVRT<br>SLRLQHRMRRRPLRPILHSRMHKHYYNKHPHHY<br>NLPRNNMWRTLPWTLHNMFCHQDPTSNLPVL<br>MNSNSMPPIPLRPTHTPPMKKLPTTHPSITYMM<br>CLHTHYNLQHSPSNLRNMSDKRVTLME

4470	5511	347	ATT	CCCGTACTA ATT A	C	A	TAG	**ND2**	INPLAQPVIYSTIFAGTLITALSSHWFFTWVGLEM<br>NMLAFIPVLTKKMNPRSTEAAIKYFLTQATASMIL<br>LMAILFNNMLSGQWTMTNTTNQYSSLMIMMA<br>MAMKLGMAPFHFWVPEVTQGTPLTSGLLLLTW<br>QKLAPISIMYQISPSLNVSLLLTLSILSIMAGSWGG<br>LNQTQLRKILAYSSITHMGWMMAVLPYNPNMT<br>ILNLTIYIILTTTAFLLLNLNSSTTTLLLSRTWNKLTW<br>LTPLIPSTLLSLGGLPPLTGFLPKWAIIEEFTKNNSLI<br>IPTIMATITLLNLYFYLRLIYSTSITLLPMSNNVKMK<br>WQFEHTKPTPFLPTLIALTTLLLPISPFMLMIL
5904	7563	553	ATG	CCCCCACTG ATG T	C	T	TAG	**COX1**	MFADRWLFSTNHKDIGTLYLLFGAWAGVLGTAL<br>SLLIRAELGQPGNLLGNDHIYNVIVTAHAFVMIFF<br>MVMPIMIGGFGNWLVPLMIGAPDMAFPRMN<br>NMSFWLLPPSLLLLLASAMVEAGAGTGWTVYPP<br>LAGNYSHPGASVDLTIFSLHLAGVSSILGAINFITTI<br>INMKPPAMTQYQTPLFVWSVLITAVLLLLSLPVLA<br>AGITMLLTDRNLNTTFFDPAGGGDPILYQHLFWF<br>FGHPEVYILILPGFGMISHIVTYYSGKKEPFGYMG<br>MVWAMMSIGFLGFIVWAHHMFTVGMDVDTR<br>AYFTSATMIIAIPTGVKVFSWLATLHGSNMKWSA<br>AVLWALGFIFLFTVGGLTGIVLANSSLDIVLHDTYY<br>VVAHFHYVLSMGAVFAIMGGFIHWFPLFSGYTL<br>DQTYAKIHFTIMFIGVNLTFFPQHFLGLSGMPRR<br>YSDYPDAYTTWNILSSVGSFISLTAVMLMIFMIW<br>EAFASKRKVLMVEEPSMNLEWLYGCPPPYHTFE<br>EPVYMKSRQKRKESNPPKLVSSQPHGLHDFFKKV<br>LEKPFHNFVKVKL
8502	8715	71	ATT	AAATAAAAA ATT T	A	T	TAA	**ATP8**	IMTNPENQNERKSVRFIHCPHNPRPTRRSTDHSI<br>SPSIDPHLQMSHQQPTNHHPTMTNQTNLKTND<br>NHTQH
8799	8883	28	ATT	GCCTCACTC ATT A	C	A	TAA	**ATP6**	IYTNHPTIYKPSHGHPLMSGHSDYRLSL

9204	10764	520	ATA	CGACAACAC ATA T	C	T	TAA	**ATP6**	MMTHQSHAYHMVKPSPWPLTGALSALLMTSGL AMWFHFHSMTLLMLGLLTNTLTMYQWWRDV TRESTYQGHHTPPVQKGLRYGMILFITSEVFFFAG FFWAFYHSSLAPTPQLGGHWPPTGITPLNPLEVP LLNTSVLLASGVSITWAHHSLMENNRNQMIQAL LITILLGLYFTLLQASEYFESPFTISDGIYGSTFFVAT GFHGLHVIIGSTFLTICFIRQLMFHFTSKHHFGFEA AAWYWHFVDVVWLFLYVSIYWWGSYSFSMNS TVNFQLTSFDNIQKRVMNFALILMINTLLALLLMII TFWLPQLNGYMEKSTPYECGFDPMSPARVPFS MKFFLVAITFLLFDLEIALLLPLPWALQTTNLPLMV MSSLLLIIILALSLAYEWLQKGLDWTELVYSLNKTN DFDSLNYDNHIYQMPLIYMNIMLAFTISLLGMLV YRSHLMSSLLCLEGMMLSLFIMATLMTLNTHSLL ANIVPIAMLVFAACEAAVGLALLVSISNTYGLDYV HNLNLLQC
11115	11220	35	ATA	TCATATTTT ATA C	T	C	TAG	**ND4**	MSSSKPHLSPPWLSSPDEATSQNAWTQAHTSYS TP
12165	12228	21	ATT	AAACATCAG ATT T	C	T	TAA	**TRNH**	IVNLTTEAYDPLFTEKAHKNC
12234	12408	58	ATG	TGCTAACTC ATG C	C	C	TAA	**TRNQ-comp**	MPPCLTTWLSQLLKDNSYPLVLGPKNFGATPNKS NNHAHYYNHPNPDFPNSPHPYHPR
12429	12678	83	ATA	AAAAAACTC ATA C	C	C	TAA	**ND5**	MPPLCKIHCRIHLYYQSLPHNNIHVPRPRSYYLELT LSHNPNNPALPKLQTRLLLHNIHPCSIVRYMVHH RILTVMYKLRPKH
12747	12954	69	ATT	TAACAACCT ATT C	C	C	TAA	**ND5**	IPTVHRLRGRRNYILLAHQLMMRPSRCQHSSHSS NPMQPYRRYRFHPRLSMIYPTLQLMRPTTNSPS KR
13014	13143	43	ATT	ATCAGCCCA ATT G	C	G	TAG	**ND5**	IRSPPLTPLSHRRPHPSLSPTPLKHYSCSRNLLTHPL PPPSRK
13170	13386	72	ATG	TCTAACACT ATG T	A	T	TAA	**ND5**	MLRRYHHSVRSSLRPYTKWHQKNRSLLHFKSTRT HNSYNRHQPTTPSIPAHLYPRLLQSHTIYVLRVHH PQP
13473	13602	43	ATT	CAGCCTAGC ATT G	A	G	TAG	**ND5**	ISRNTFPHRFLLQRPHHRNRKHIMHKRLSPIYYSH RYLPDKRL
14145	14208	21	ATA	CCTAATCAC ATA C	C	C	TAA	**ND5**	MTYSPEQSQLQYMHQQTMFNQ

14703	14775	24	ATG	CCACGACCA ATG T	C	T	TAA		MMWKTIVVFQLQEHQWPQYAKLTP
14967	15117	50	ATT	GAGACGTAA ATT T	T	T	TAG	**CYTB**	IMAESSATFTPMAPQYSLSASSYTSGEAYITDHFS TQKPETSALSSCLQL
15387	15462	25	ATT	TCACCTCCC ATT C	C	C	TAA	**CYTB**	IPMKSPSTLTTQSKTPSAYFSSFSP
16062	16161	33	ATT	CACCCAAGT ATT A	A	A	TAA		IDSPINNRYVFRTLLPATMNIVRYHKYLTTCST
16194	16308	38	ATG	CCCCTCCCC ATG T	C	T	TAG		MLTSKYSNQPSTITHQLQLQSHPSPTRMPTNLPT LNST
16314	16386	24	ATA	ACATAGTAC ATA A	T	A	TAG		MKPFTVHSTLQSNPFSSPWMTPLR
-16315	-16087	76	ATG	AATGGCTTT ATG A	T	A	TAG		MYYVLLRVGRFVGILVGEGWLWSCSWCVMVEG WLLYLLVSMGRGFWCGLGFYVLQVVKYLWYRT MFMVAGSNVRNT
-16075	-16006	23	ATG	CGGTTGTTG ATG G	T	G	TAG		MGESMLGWYPNLLPHERTENSLN
-15139	-15052	29	ATA	CGGGAGGAC ATA C	G	C	TAG	**CYTB-comp**	MAYEGCCYSCKQEDNADVSGFWVEKWSVM
-15001	-14899	34	ATT	TGAGGCGCC ATT G	G	G	TAG	**CYTB-comp**	IGVKVADDSAMIYVSSDVGDWWKGGWGVWW VVHG
-14719	-14611	36	ATG	AATACAACG ATG T	A	T	TAA		MVFHIIGRGCSPCENNDVCFVSVECGFSNGVCG VFF
-14470	-14320	50	ATA	TGTCTTTGG ATA A	T	A	TAA		MYYSDGYWGVSWGMGVRGWGLGECFSGVSD GGRIGAVGERVWWGGGCGKL
-14221	-14152	23	ATT	TGGGCGTTG ATT G	T	G	TAG		ISSSYWLNIVCWCMYCNWDCSGE
-13564	-13387	59	ATA	GAGTAATAG ATA G	T	G	TAA	**ND5-comp**	MGLRRLCMMCLRFRWCGLWSRNLWGKVFLLM LGCQWWGRLKWEVWFWVVLLFFEYLVHC
-13354	-13282	24	ATA	AGCACATAA ATA T	T	T	TAA	**ND5-comp**	MVWLWRRRGYRCAGMLGVVGWCRL
-13171	-13090	27	ATA	CGCCTAAGC ATA T	A	T	TAG	**ND5-comp**	MVLEFGLVGYFLLGGGSGWVRRFLLQL
-13015	-12955	20	ATT	GGAGACCTA ATT G	C	G	TAG	**ND5-comp**	IGLICLLLLGGGLVVGWGLD
-12898	-12784	38	ATG	GGATAAATC ATG T	A	T	TAA	**ND5-comp**	MLRRGWNRYRRYGCMGLLEWLLCWHLLGRIIN WWARRM
-12616	-12523	31	ATG	TAACGAACA ATG T	A	T	TAA	**ND5-comp**	MLQGWMLWRSSLVWSLGRAGLFGLWLSVSSR
-10711	-10483	76	ATT	GTGTTGGAG ATT A	G	A	TAA	**TRNY**	IETSRARPTAASQAAKTSMAMGTMLAKREWVL RVMRVAMMNSDSIIPSRHSREDMRCERYTSIPR SEMVNASMMFM
-10348	-10168	60	ATG	AGGGCTAGG ATG T	A	T	TAA	**ND3-comp**	MMINKRDDMTISGRLVVCRAHGRGKRRAISRSN NKKVMATKKNFMEKGTRAGDMGSKPHS

-10117	-9991	42	ATA	CAAAATGTA ATA T	G	T	TAA	**ND3-comp**	MIISSKARRVLIIKIKAKFITLFWMLSKLVNWKLTV LFMLKE
-9973	-9784	63	ATG	CATCAATAG ATG A	T	A	TAG	**COX3-comp**	METYRNSQTTSTKCQYQAAASKPKWCLDVKWN ISWRMKQMVRKVEPMMTWSPWKPVATKNVE P
-9781	-9721	20	ATG	GAGCCGTAG ATG C	T	C	TAG	**COX3-comp**	MPSEMVKGDSKYSEACRRVK
-9706	-9511	65	ATT	CCCAGTAAA ATT T	A	T	TAA	**COX3-comp**	IVMSSAWIIWFRLFSIRLWWAQVIDTPDASNTD VFRSGTSRGFSGVMPVGGQCPPNWGVGARLE W
-9466	-9229	79	ATA	TCTGAGGTA ATA A	G	A	TAG	**COX3-comp**	MNRIIPYRRPFWTGGVWWPWYVLSRVTSRHH WYMVSVLVSRPSMRSVMEWKWNHMARPEVI RRAERAPVRGHGLGFTMW
-9208	-9127	27	ATT	TGGTGGGTC ATT T	G	T	TAG	**COX3-comp**	IMCCRAGRGLLEVWKRRLGLRRQRFLG
-8668	-8470	66	ATT	TGATTAGTC ATT T	G	T	TAG	**ATP6-comp**	IVGWWLVGCWWDIWRWGSMEGEMEWSVLR RVGLGLWGQWMKRTDFRSFWFSGFVMIFYFYG LWWGR
-7372	-7231	47	ATG	TCCAGGTTT ATG A	T	A	TAG	**COX1-comp**	MEGSSTIRTFRFEAKASQIMKIINITAVREMNEPT DDRMFHVVYASG
-7210	-7129	27	ATT	CGTCGGGGC ATT C	G	C	TAG	**COX1-comp**	IPDRPRKCCGKKVRFTPMNMMVKWILA
-6997	-6685	104	ATG	TGTAGTACG ATG C	A	C	TAG	**COX1-comp**	MSSDEFANTMPVRPPTVKRKMNPRAQSTAADH FMLLPWSVASQLNTLTPVGMAMIMVAEVKYAR VSTSIPTVNMWCAHTMNPRKPIDIMAQTMPM YPNGSFFPE
-6589	-6310	93	ATG	TGGTATAGA ATG G	A	G	TAG	**COX1-comp**	MGSPPPAGSKKVVLRLRSVSSMVMPAARTGRD RRSRTAVIRTDQTKRGVWYWVMAGGFMLMIV VMKLMAPKMEETPARCKEKMVRSTEAPGWE
-6253	-6064	63	ATA	GCCTCCACT ATA C	A	C	TAG	**COX1-comp**	MADASRSRREGGKSQKLMLFMRGNAMSGAPII RGTSQLPKPPIMMGITMKKIITNAWAVTMTL
-6061	-5842	73	ATG	ACGTTGTAG ATG G	T	G	TAG	**COX1-comp**	MWSLPRRLPGWPSSARMRRLRAVPRTPAHAPN NRYSVPMSLWFVENSQRSANISGGEVKWLSEAL DCKSKDRG
-5806	-5710	32	ATT	GATTTTCAT ATT A	C	A	TAG		IELQIRRSSFKPAGASPAFFPGGGRSRLKPVD
-5641	-5572	23	ATT	CTTAGCTTA ATT A	T	A	TAA		IKVADLRSVDAEWGFAVLSCYRN

-5506	-5362	48	ATT	TTCTATAAG ATT T	A	T	TAG	**ND2-comp**	IISMKGEMGRSSVVRAMSVGRNGVGFVCSNCH FIFTLLDMGSSVIEVE
-5323	-5092	77	ATG	AGGAGGGTG ATG T	G	T	TAA	**ND2-comp**	MVAMMVGMMRLLFFVNSSMMAHLGKKPVSG GRPPRERRVDGIKGVSHVSLFQVRDSSRVVVLEF KLSSRNAVVVRMM
-5089	-4498	197	ATA	ATAATATAA ATA T	T	T	TAG	**ND2-comp**	MVKLRMVMLGLYGRTAIIHPMWVIEEYAKILRS WVWFNPPQLPAMMDKIERVRRRLTFSEGEIWY MIEMGASFCHVRRSRPDVRGVPWVTSGTQKW KGAIPSFIAMAIMIINDEYWLVVLVMVHCPESML LKRMAIRRIMDAVACVRKYLMAASVERGFIFLVR TGMKASMFISRPTQVKNQCELSAVMSVPAKMV E
-4396	-4315	27	ATG	TACTTTAGG ATG G	A	G	TAA		MGCDRWHGEFWILRDGFDSHSPRNKGV
-4306	-4234	24	ATT	TAAGCTCCT ATT T	C	T	TAA	**TRNI-comp**	IIYSIKVTLLSDMFLRFEGECWRL
-4204	-4024	60	ATG	ATATAAGTA ATG T	G	T	TAG	**ND1-comp**	MLGWVVGSFFMGGVWVGRSGIGGMLFEFMR TGRLEVGSWWQNMLCRVQGRVRHMLFLGRL
-3988	-3865	41	ATT	TGTTTGTGT ATT G	T	G	TAA	**ND1-comp**	IRLWRMGRRGLRRIRCWSLRLVRTPLRQGRRGF GWSLLVWR
-3766	-3634	44	ATG	TTATTAGTA ATG T	G	T	TAG	**ND1-comp**	MLMVEWWLGWLHMRLFGLLLAVRRSGRSLSL MLTLIRGLSKRLG
-3616	-3505	37	ATA	CTAGAATAA ATA G	T	G	TAG	**ND1-comp**	MGGLGWGWPGGWVWGGGFMVEERWWELR SGRWCRGWW
-3502	-3373	43	ATG	TGATGGTAG ATG G	T	G	TAA	**ND1-comp**	MWRVLGALWWRVLWRQRRVVVARRGLQRW GLCVVVVYSLEFFVR
-3361	-3301	20	ATG	GCATTAGGA ATG C	G	C	TAA	**ND1-comp**	MPLRLEWVQWGVGGWPWVCC
-3253	-3151	34	ATT	TTTTATGCG ATT C	G	C	TAG	**TRNL1-comp**	ITGLCHLNKPCSWVGVGMMLSWDDIIYGGRRFV K
-2887	-2782	35	ATA	CAATTGAGT ATA T	A	T	TAG	**RNR2-comp**	MVVRFDWWSLSMYCSEVGFCSEVAPTEIFNAGL VV
-2731	-2668	21	ATA	TAAAGCTCC ATA G	T	G	TAA	**RNR2-comp**	MGSSRLAVLCPPLHGQVNFTG
-1861	-1705	52	ATT	TTCTAGTTA ATT A	T	A	TAG	**RNR2-comp**	IHYAEGMGVSPCYIMLGYNFSSFPCGTMSIAPGF NFYRLYFIWVNGLAKVVW
-1267	-1159	36	ATG	TTGCTGAAG ATG C	A	C	TAA	**RNR1-comp**	MAVYRLSKRWWGWSGFIDYRTGSSRGMWSTA RSFEF

-673	-532	47	ATA	TAAGAGCTA ATA A	C	A	TAG	**RNR1-comp**	MERLGPNLFVYGVMWARLNIFSVLLWGGKLHKL WGVSLGFGWFGVWG
-292	-214	26	ATT	TTGGTGGAA ATT T	G	T	TAA		IFCYDVCVESGCADIQLLLLCPTSIN
-157	-13	48	ATA	GTGCGATAA ATA T	T	T	TAG		MMGWGRNQRQMLRHRVLRLQRLAMLSRAYP PDENTKCMESSREWLMGW
-16500	-16326	58	ATG	AGGAACCAG ATG C	C	C	TAA		MSDTVHFSYPQVLWARSEESSTLVRDIDFTEDGG QGTPIWGGSSMGTRRDLTVMCYVR
-16161	-16011	50	ATG	TGGGTTTTT ATG A	T	A	TAA		MYYRWSSIYGTVQYSWWLAVMYEMHSGCWW VSQYLGGTQICFPMKEQRMV
-16008	-15906	34	ATT	ATAGTTTAA ATT G	T	G	TAG		IRILALGANGGVKDFFSDLSLEKGFHLRFTRLVY
-15900	-15780	40	ATA	TATTAGTTT ATA T	T	T	TAG	**TRNT-comp**	MLQGQAHLSILFSIREMVGIRIRIVVKYSTDATCP MMVKG
-15693	-15579	38	ATT	GGGCGAAAT ATT T	A	T	TAG	**CYTB-comp**	IMLCCLDMWRMGIIARMRMDSNRARTPPSLLG TDRRIV
-15399	-15249	50	ATT	TGGAAGGTG ATT T	G	T	TAG	**CYTB-comp**	ILSEWEVIPRGLFDPVSCKNRRWSVARAAMMKG KMKWKVKNRVRVGLSTE
-15237	-15138	33	ATT	CCTCCTCAG ATT A	C	A	TAG	**CYTB-comp**	IHWTRSVPMYGMADSKFVITVAPQNDIWPHGR T
-15132	-15072	20	ATG	ACATAGCCT ATG A	C	A	TAG	**CYTB-comp**	MKAVAMVASRRMMPMFQVSE
-14904	-14139	255	ATG	GAGTAGTGC ATG C	T	C	TAG	**CYTB-comp**	MARNSPVVIWRIRQAPRSEPKFHHAEMLDGVG RSMNEWLINFIRGLVLRIGVIGVLVVEMQRWFF MSLVVVVVRARMMMYALFLLSVGLVMGFVGFS SKPSPIYGGLVLIVSGVVGCVIILNFGGGYMGLMV FLIYLGGMMVVFGYTTAMAIEEYPEAWGSGVEV LVSVLVGLAMEVGLVLWVKEYDGVVVVVNFNSV GSWMIYEGEGSGLIREDPIGAGALYDYGRWLVV VTGWTLFVGVYIVIEIARGNRLCD
-13953	-13887	22	ATA	AAGGCCTAG ATA G	T	G	TAG	**ND5-comp**	MGDCAVCDARVESEYVGEMKCA
-12696	-12582	38	ATA	GATGAGTAG ATA T	T	T	TAG	**ND5-comp**	MFEELINVWVWVYMSQWEFYDGPCNEQCYRD EYYGEVV
-12441	-12381	20	ATA	GGATTTTAC ATA T	T	T	TAG	**ND5-comp**	MMGVWVFFVRVNEGGKDGGN
-12291	-12204	29	ATG	CTAAGACCA ATG A	C	A	TAA	**TRNL2-comp**	MDSCYPLKVEKAMLLDMGAWVSSSCELSR

-12201	-11985	72	ATA	TCTCGGTAA ATA G	T	G	TAG	**TRNQ**	MRGRKPLLSDSQSDVLVKLYLQEENPVMMSGLR<br>DRRRMGDRCMNMRVFSRVNEGFMLLMWWV<br>SEPHCVVVNM
-11958	-11760	66	ATG	GTGACTAGT ATG T	A	T	TAG	**ND4-comp**	MLSPVSRRVMFDQENVVTSTESSPSRLMVGGKA<br>RLARLARSHQKAISGSRVWSPWERIMMRLWVR<br>S
-11709	-11535	58	ATT	CCGTGGGCG ATT T	G	T	TAG	**ND4-comp**	IMRMTAPVKLQGVWMRMAVTTRAMWLIEEYA<br>MSDFRSVCRRQMELVMIMPHRDSTRKG
-11343	-11256	29	ATT	GCTAGTCAT ATT A	C	A	TAA	**ND4-comp**	IKLLAQEFDSSWAVRVSSRMFSEPRVLWV
-11109	-11043	22	ATG	ATATAAAAT ATG T	A	T	TAG	**ND4-comp**	MISSVAVNVMIKEICREISMER
-10992	-10650	114	ATG	TGGCTTGCC ATG T	G	T	TAG	**ND4-comp**	MIVRGRSQVVSIRRGVVRGSEEKVGEQLNRLLLI<br>WLKNSRGMMLMIRLWVVVLIQIMCFLESHVSG<br>SNMIVGTISFSIGVGLGYVRSLGHMCWRLRLVGL<br>GPPLLRRRQRLVWQ
-10584	-10506	26	ATA	TGAACAGCG ATA T	G	T	TAA	**ND4L-comp**	MVLFLLGMVGRMWGVSDMLVFLEVRW
-10443	-10323	40	ATG	CATAATTTA ATG G	T	G	TAA	**TRNR-comp**	MSRNHSFCLNYMPIRFSLILFVVTHRPDLGLGW<br>WLMRGMT
-9957	-9888	23	ATA	CATACAGAA ATA T	G	T	TAA	**COX3-comp**	MVKPHLQNASIRRRLRSQSDVWM
-9795	-9726	23	ATG	CTACAAAAA ATG T	A	T	TAA	**COX3-comp**	MLSRRCRRKWWRETRSTLRLVGG
-9633	-9531	34	ATA	AGGTGATTG ATA T	T	T	TAG	**COX3-comp**	MLLMRVMRMCLGVGLLGDLAGWCLLGASALLI<br>GG
-9462	-9177	95	ATA	AGGTAATAA ATA G	T	G	TAG	**COX3-comp**	MGLSRIEGLFGQVVCGGLGMCFLVLHRAIIGMW<br>LVCWLVGLVWGALWSGSEITWLGRRSLGGLRG<br>PLLGVMGWVLLYDRHVIGGSLCVVVQVEAY
-8448	-8358	30	ATT	GTGTTTAAT ATT T	A	T	TAA	**ATP8-comp**	IFSWVMRNSVRSMGVIMVGHTVVFSWGISL
-8247	-8163	28	ATT	ACGGGCCCT ATT C	C	C	TAG	**COX2-comp**	ISKIFRGINSRTMGMKLWFAPQISEHWP
-8109	-7947	54	ATT	CGTCCGGGA ATT C	G	C	TAG	**COX2-comp**	IASVFKPNVGTAHECKTSCDVIIMRMGASIGSTTR<br>LSTSRSRRSPGSRNNGGSM
-7833	-7569	88	ATG	ATGTAAAGG ATG G	A	G	TAG	**COX2-comp**	MRRDGRAMRTRMMAGRMVQTVSISWASEML<br>VLVSFVVSVRKRAYRTRKQMRKMIMRAWSWKV<br>MSSSMMGEVASCRPTCAACAIKMYRI
-7536	-7443	31	ATG	AGTTATGAA ATG T	G	T	TAG	**TRND-comp**	MVFLMPFWKSHGGHGVGLKPALGGSIPSFFV

-7440	-7149	97	ATT	TTTGTCTAG ATT T	T	T	TAG	**COX1-comp**	ILCMRVLRMCGRVGGIHMVTPGLWRVLLLLGLF ASKRRLLKSWKLLMLLLLEKWMSLQMMGCFM WCMHRGSPSNVGAFRMGRESVVGRKLDLRRW MW
-7101	-6867	78	ATA	AGCCTGAGA ATA G	A	G	TAA	**COX1-comp**	MGEISEWSLLWWQMQLLLMGHSGSGLQRSTC RVVRCLVMSLLMQCQSGHLRWKERWILGLRAL QQIISYCFRGVWRVS
-6705	-6642	21	ATG	TGTATCCAA ATG T	C	T	TAA	**COX1-comp**	MVLFFRSSKLQYGRLFRSLVG
-6606	-6525	27	ATA	AAAATCAGA ATA G	A	G	TAG	**COX1-comp**	MGVGMEWGLLLRRGRRRWCWGCGLLVV
-6495	-6408	29	ATA	CTGGGAGAG ATA G	G	G	TAA	**COX1-comp**	MGEVGLLWLGRIRRRGAFGIGLWQGVLYW
-6246	-6072	58	ATG	CTATAGCAG ATG G	C	G	TAA	**COX1-comp**	MRAGVGEREVRVRSLCCLCGETPYRGHRLLGELV SCQSLRLWWVLLWRRLLQMHGLWR
-5814	-5694	40	ATT	TCCGAGGTG ATT T	G	T	TAA		IFMLNCKFEEAASNLPGLLPPFFPAAGEVDWSQL IRVLSC
-5628	-5520	36	ATT	AAGTGGCTG ATT G	C	G	TAA		ICVQLMQSGVLQSLAVTEIKYCNLLRALKALGLYL T
-5259	-5109	50	ATT	TAATGGCCC ATT G	C	G	TAG	**ND2-comp**	IWAKSRLAGAGLLGRGGWMELRVLVMLACFRC EMVVGSWCWSLSWVVGMR
-5010	-4824	62	ATG	TTGAGGAGT ATG T	A	T	TAA	**ND2-comp**	MLRFCVAGFGLIHLNCLLWWMRLREWGEGLRL VRERFGMWLRWGLVFVMWEEAGRMSEGCLG
-4668	-4590	26	ATG	GGATTATGG ATG G	T	G	TAA	**ND2-comp**	MRLLAWGNTWWQLLWNEGLFFWLELE
-4434	-4287	49	ATG	TTTCGGGGT ATG G	G	G	TAA	**TRNM-comp**	MGPMAYLADLTLGWGVMGGTENFGFSGMGSI LMVLEMRGFKLLLFTLSK
-3732	-3639	31	ATA	GGTGACTTC ATA G	T	G	TAG	**ND1-comp**	MWDCLGYCSQCADQGVVWVWCSPWSEDWV NG
-3186	-3105	27	ATG	TAAGTTGAG ATG T	G	T	TAG	**RNR2-comp**	MMSFTGEGALWSRPYFSCPFVQGGIWX
-3006	-2931	25	ATG	ACCATCGGG ATG C	G	C	TAG	**RNR2-comp**	MSWSNIEVVNPIVDMDSRMGLRCYP
-2679	-2487	64	ATT	GGCAGGTCA ATT C	T	C	TAA	**RNR2-comp**	ISLVKSKRQLNPRGAIHTGPYLRNKWLCYLCTVRV PRPLNMCHWAGGASNTGDARGDVFGKQAG
-1824	-1731	31	ATA	GTCCTTGCT ATA T	G	T	TAA	**RNR2-comp**	MLCLVMIFHLSLAVLYLLRQVSISIAYTLFG
-1506	-1374	44	ATA	CTTTGAAGT ATA T	A	T	TAG	**RNR1-comp**	MLEEGDGRCVRASGPCSTKHSTLSLLLNPPSTLKF HKGYRSFLG
-1257	-1116	47	ATA	TGGCGGTAT ATA G	T	G	TAA	**RNR1-comp**	MGWARGGEVDRGLSITEQAPLEGYEAPPGPLSF KLWLVVFWRAVLLI

-582	-402	60	ATA	GTAAGCTAC ATA A	T	A	TAA	**TRNF-comp**	MNCGGCLWGLVGSGYGVSSGVCVLGRMGGGC IDEISSMGVGGENNVLVGGWLLKVHTAKR
-342	-210	44	ATG	TTGGCAGAG ATG G	G	G	TAA		MCLSAVARSGGGGVWWKFFVMMSVWKVAVQ TFNCYYYVLQALIN
-117	-24	31	ATA	TACTGCGAC ATA G	G	G	TAA		MGCSGSSVSQCYRVHTPQTKMPNAWRAPVSG
-16562	-16343	73	ATG	TCCATCGTG ATG C	G	C	TAA		MSYLRGTCGLFRLYDPEVGTRCRMQFTLATPKCY GPGARRVALLCGMLISRRMVVKGPLSEGGHPW GREGIWL
-16238	-16103	45	ATG	TTGCAGTTG ATG G	T	G	TAA		MCDSWGLIAVLACKHGEGVLMWIGFLCTTGGQ VFMVPYNIHGGWQ
-16088	-16016	24	ATA	ACGAAATAC ATA C	T	C	TAG		MAVVDGWVNTWVVPKSASPWKNRE
-15983	-15851	44	ATG	TGGGTGCTA ATG T	C	T	TAG		MVELKTFSLICPWKKVFISGLQDWCISLYYKDRPI WVFCFQLGR
-15713	-15602	37	ATT	AATAAAGTG ATT G	G	G	TAG	**CYTB-comp**	IGLVGEMLCFVVWMYGGWGLLLGWGWMVM GQGRLLVC
-15572	-15311	87	ATA	TGTAGGCGA ATA G	C	G	TAA	**CYTB-comp**	MGNIIRAWCGEGCLRGWLGYNCLGRLGGLVRM VLMSLRREGREVSRGRLWLCSKGGRWFYRNGR WFLGGCLIPFRARMGGGVLLGLQ
-15014	-14789	75	ATA	AGATAAAGA ATA T	A	T	TAA	**CYTB-comp**	MLRRHWREGSGWFSHNLRLEWCGRLMKRRLR RLVSSAWLGMVLWWFGGSGRRQGVSRSFIMR RCWMGWGGRWMSG
-14468	-14396	24	ATA	TCTTTGGAT ATA T	G	T	TAG		MLQRWLLRSILRHGGQGLRSWWVF
-14294	-14219	25	ATG	GAATAATTT ATG A	T	A	TAG		MKERGQGWFGRILLVRGLCMIMGVD
-14087	-13952	45	ATT	TAAAGTTTA ATT T	T	T	TAG	**ND5-comp**	IMPFWVEVMMEVEIWCCEIVLGNSFSSQVRSRR SRGRFWLVRRPR
-13781	-13601	60	ATT	TAGAGGGGG ATT T	G	T	TAG	**ND5-comp**	IVVWKGDAGEMLLVMRNPANRLPAARRLMGFS RVGLFSLMLVRVGKRGWPVRVRRIIRVL
-13577	-13505	24	ATG	GAGGTAGCG ATG G	G	G	TAG	**ND5-comp**	MRVMDRAQAFVYDMFAVSMMWSLE

-13484	-12587	299	ATT	AGGAAAGGT ATT C	G	C	TAG	**ND5-comp**	IPANARLPMVREVEVRGMVLSSPPIFRMSCSLLR LWMMDPEHMNSMALKKAWVQMCRNARCG WLMPIVTIMSPSWLEVEKATIFLMSFCVRAQTA ANRVVMAPKHSVRVWISGLFSARGWKRMSKKI PATTMVLEWSRAETGVGPSMAEGSQGWRPN WADLPAAARRRPSSGVRLGLAFRRAICCGSHELE CRMNHAKARMKPMSPMRLYRIAWMAAVLASA RAYHQLMSKKDMIPTPSQPMNSWNRLLAVTKIS MVIRKMSRYLKNWLMFGSEFMYHSENSMMDH VTNNATGMNIMEK
-12524	-12440	28	ATA	CAGTTCGAG ATA T	G	T	TAA	**ND5-comp**	MMTSWSRHMNIVVGKRLMMKVDATMDFT
-12392	-12299	31	ATG	GTGGTAAGG ATG G	A	G	TAA	**ND5-comp**	MGGIREVRVRVVMVVCMVITFIWSCTKIFGA
-12101	-12029	24	ATA	GGTTGAGGG ATA G	G	G	TAA	**ND4-comp**	MGGEWGMGVWTWGCFLVWMRVLCC
-11558	-11411	49	ATA	TTATGCCTC ATA G	C	G	TAG	**ND4-comp**	MGMVQGRGRLCVLSGGWEWVWGVLYHSRLV LRVLRQVLLTQRWGLRHGL
-11345	-11153	64	ATA	AAGCTAGTC ATA T	G	T	TAG	**ND4-comp**	MLSCWLRSLMVLGQWEWVVECLVSLGCCECKL VRWVGEGSLLGCRMGSMCLRSGVLAGCLIGW W
-11027	-10967	20	ATA	TTTTTCGTG ATA T	G	T	TAG	**ND4-comp**	MVVHWMSGVGLPWLWGVGVR
-9935	-9875	20	ATG	ATCTACAAA ATG C	A	C	TAG	**COX3-comp**	MPVSGGGFEAKVMFGCKVKY
-9152	-8420	244	ATT	TAGGCTTGG ATT A	T	A	TAA	**ATP6-comp**	IKATAISRMVSRIRIVKMMSVEGRLMVDIARVAL PIRCMSRWPAVMLAVRRTARAIGWMSRLMVS MMTSMGMRGVGVPCGKKWARAFLILERKPMI TVPAHKGMAMARFMDSWVVGVNEWGRSPRR LVVAMKMIKDTSMRDQVRPLVLCMVIICFEVSLI SHCWVVISRLLMRYLEVGINRGGNRMISTAAGR PRIVGAMNEANRFSFILVLRVCYNFLFLWALVRE VGGSLCLMFLVGWWGMV
-8258	-8144	38	ATA	ATAGGGTAA ATA G	T	G	TAG	**COX2-comp**	MRALFQRFLGELILGRWAWNCGLLHRFQSIDRS MPPVV
-7964	-7871	31	ATA	GTTCTAGGA ATA T	G	T	TAA	**COX2-comp**	MMGEVCRSWRLVRRSRCTRRFSTIGGQLIWW
-7505	-7388	39	ATG	GAAAAAGTC ATG A	G	A	TAG		MEAMGLAWNQLWGVRFLPFLSRFYVYGFFECV VGWGASM

-6896	-6752	48	ATT	TCATTTCAT ATT C	C	C	TAG	**COX1-comp**	IASVECGESAKYFDAGGDSDDYGSGGEMCSCVY VYSYCKYMVCSHDKP
-5801	-5729	24	ATT	TCATATTGA ATT C	T	C	TAG		IANSKKQLQTCRGFSRLFSRRREK
-4742	-4682	20	ATT	TGAGTATTG ATT G	T	G	TAG	**ND2-comp**	IGSIGYGSLSGEYIVEEDSY
-4247	-4151	32	ATG	TGAGGGGGA ATG T	G	T	TAG	**ND1-comp**	MLEIVMGMETYHMSNARVSGRKFFHRRCMSW S
-4028	-3686	114	ATT	CCTAGGAAG ATT T	A	T	TAG	**ND1-comp**	IVVVRVFIMMMFVYSAMKNRAKGPAAYSMLKP ETSSDSPSARSKGVRLVSASVEMNHIMAKGHDG RSNQRCSCVVMRVERLKEPLISNVDSRMMARVT SYEIVWATARSAPIRA
-3620	-3518	34	ATA	GTGGCTAGA ATA A	A	A	TAG	**ND1-comp**	MNRRPRLRLTRGLGMGRGVHSRRAMVRAKVG AVM
-3509	-3434	25	ATG	TAGAGGGTG ATG T	G	T	TAG	**ND1-comp**	MVDVAGFRGSLVKSFMASAKGCSSP
-3386	-3266	40	ATT	TAGCCTAGA ATT T	A	T	TAA	**ND1-comp**	IFRSVSIRNAIAIRMGTMRSRRLAMGMLLRRGIE PLTVKF
-2750	-2615	45	ATT	ACTGTTTGC ATT A	T	A	TAA	**RNR2-comp**	INKLKLHRVFSSCCVMPASSRAGQFHWLKVRDS WTLVEPFMQVPI
-2531	-2303	76	ATA	GTGCCTCTA ATA T	C	T	TAA	**RNR2-comp**	MLVMLEVMFLVNRRGKICRVPFTFFNLSLWACL CWVDSEGNNDLLVDCRYWAVNCQFSVLIWRRL MRRRMFSCYLY
-2279	-2195	28	ATA	TATAGGGTG ATA A	G	A	TAA	**RNR2-comp**	MDWSNWVWGVQLYVWDFLGSGCWAWTLS
-2168	-2090	26	ATG	AGGCCTACT ATG G	A	G	TAA	**RNR2-comp**	MGVKFFTLSTRFFPSVQRAVPLWTNS
-1520	-1427	31	ATG	TTTAGTTAA ATG C	T	C	TAA	**RNR1-comp**	MSFEVYLRRVTGGVYALQGPVQLSTLLLVYC
-1400	-1292	36	ATA	TTAAGTTTC ATA G	T	G	TAG	**RNR1-comp**	MRAIVVFWGRKCSPFLATSWATPWPNVFTWVL ALTL
-1091	-1022	23	ATA	GGGCTAAGC ATA T	A	T	TAG	**RNR1-comp**	MVGYLIPVWVLAIVCSDMLKPLS
-1016	-950	22	ATT	TCGTAGTCT ATT T	T	T	TAA	**RNR1-comp**	ILCQLEFFTTQVSFSFIGEGVI
-773	-590	61	ATT	TTGAGCTGC ATT C	T	C	TAA	**RNR1-comp**	IAACLMLVPFDRGDLEGELTGTGMLACVILLRAN RKARTKPICLWGDVSPSKHFQCIALRR
-539	-443	32	ATG	GTTCGGGGT ATG G	G	G	TAG		MGLAAVCVCWVGWAGVVLMRLVVWEWEGK MMC
-416	-335	27	ATA	TAAAAGTGC ATA C	T	C	TAA		MPPKDKIWNLVRLVLGFFVFGVWQRCV
-179	-116	21	ATA	TCGCCTGTA ATA T	G	T	TAG		MLNVGAMNNRMRQESKTDTAT

Table S2. Identification of mitochondrial smORFs and altORFs - OpenProt approach and sequences selected for antibody production.

Genomic regions without an associated transcript will not be in OpenProt (i.e. regions with no gene name, or -comp)

Genes	Present_in_OP
ATP6	1
ATP8	1
COX1	1
COX2	1
COX3	1
CYTB	1
ND1	1
ND2	1
ND3	1
ND4	1
ND4L	1
ND5	1
RNR1	1
RNR2	1
TRND	1
TRNH	1
TRNI	1
TRNK	1
TRNL1	1
TRNL2	1
TRNQ	1
TRNS1	1
TRNT	1
TRNW	1
TRNY	1

OpenProt predictions for genes above - AltProt (novel protein from non canonical ORF) are in green - sorted by MS detection - more info on each protein on OpenProt website Identification of mitochondrial smORFs and altORFs - OpenProt approach

OpenProt release 1.3 - August 30 2018

Protein accession	Protein Type	Species	Protein length (a.a.)	Molecular weight (kDa)	Isoelectric point	Gene symbol	Transcript accession	Type	MS score	TE score	Domains	Orthology Across10 Species = Species name : id %
P00403	RefProt	Homo sapiens	227	25.56	4.44	COX2	COX2	CDS	335	0	25	SC:44.09,DR:67.73, MM:73.64,RN: 74.09,DM:58.45,CE: 44.5,BT:74.55,PT: 97.8,OA:73.64
P03915	RefProt	Homo sapiens	603	67.03	9.32	ND5	ND5	CDS	110	0	62	DR:55.18,MM: 66.13,RN:64.68, DM:37.2,CE:35.31, BT:71.23,PT:93.27, OA:71.6
P03928	RefProt	Homo sapiens	68	7.99	10.56	ATP8	ATP8	CDS	88	0	7	MM:46.27,RN: 47.76,BT:55.17,PT: 94.12,OA:49.23
YP_003024035.1	RefProt	Homo sapiens	459	51.58	9.67	ND4	ND4	CDS	72	0	45	DR:60.22,MM: 67.03,RN:67.9,DM: 42.96,CE:32.93,BT: 74.56,PT:94.99,OA: 75.66
YP_003024026.1	RefProt	Homo sapiens	318	35.66	6.53	ND1	ND1	CDS	71	0	32	DR:67.2,MM:78.33, RN:77.89,DM: 49.34,CE:35.33,BT: 78.55,PT:94.65,OA: 77.99
P00395	RefProt	Homo sapiens	513	57.04	6.7	COX1	COX1	CDS	66	0	56	SC:66.05,DR:85.57, MM:91.02,RN: 90.62,DM:76.24,CE: 60.67,BT:90.82,PT: 98.83,OA:91.99
YP_003024031.1	RefProt	Homo sapiens	226	24.82	10.68	ATP6	ATP6	CDS	57	0	30	SC:35.58,DR:52.86, MM:75.66,RN: 75.66,DM:37.95,CE: 32.69,BT:77.88,PT: 94.25,OA:77.43
YP_003024032.1	RefProt	Homo sapiens	261	29.95	7.34	COX3	COX3	CDS	49	0	28	SC:43.85,DR:80.84, MM:86.97,RN: 87.74,DM:65.25,CE: 43.31,BT:87.69,PT: 97.32,OA:86.97

YP_003024027.1	RefProt	Homo sapiens	347	38.96	10.3	ND2	ND2	CDS	37	0	39	DR:44.8,MM:58.06,RN:57.76,DM:41.64,BT:63.93,PT:96.81,OA:63.85
YP_003024038.1	RefProt	Homo sapiens	380	42.72	8.22	CYTB	CYTB	CDS	28	0	37	SC:50.27,DR:70.26,MM:78.57,RN:78.63,DM:62.81,CE:44.87,BT:78.89,PT:93.67,OA:77.84
YP_003024033.1	RefProt	Homo sapiens	115	13.19	4.08	ND3	ND3	CDS	17	0	13	DR:57.52,MM:70.79,RN:73.03,DM:47.62,BT:73.91,PT:94.78,OA:73.04
IP_306405	AltProt	Homo sapiens	61	6.38	11.88	RNR1	RNR1	rRNA	1	0	8	PT:90
IP_306387	AltProt	Homo sapiens	32	3.66	11.93	ND2	ND2	CDS	0	0	0	PT:65.62
IP_306389	AltProt	Homo sapiens	33	3.7	9.51	COX1	COX1	CDS	0	0	0	
IP_306392	AltProt	Homo sapiens	39	4.56	10.98	ATP8	ATP8	CDS	0	0	0	PT:79.49
IP_306398	AltProt	Homo sapiens	35	3.82	6.5	ND4	ND4	CDS	0	0	0	PT:85.71
IP_306399	AltProt	Homo sapiens	59	6.91	12.81	ND4	ND4	CDS	0	0	0	PT:82.76
IP_306403	AltProt	Homo sapiens	49	5.22	4.14	CYTB	CYTB	CDS	0	0	0	PT:74.29
IP_306404	AltProt	Homo sapiens	31	3.43	10.22	RNR1	RNR1	rRNA	0	0	0	
IP_306406	AltProt	Homo sapiens	37	4.35	9.95	RNR1	RNR1	rRNA	0	0	0	PT:86.49
IP_306407	AltProt	Homo sapiens	32	3.27	6.5	RNR1	RNR1	rRNA	0	0	0	PT:93.75
IP_306408	AltProt	Homo sapiens	33	3.81	8.56	RNR1	RNR1	rRNA	0	0	0	
IP_306409	AltProt	Homo sapiens	32	3.71	9.44	RNR2	RNR2	rRNA	0	0	0	
IP_306410	AltProt	Homo sapiens	33	3.83	10.9	RNR2	RNR2	rRNA	0	0	0	
IP_306411	AltProt	Homo sapiens	29	3.33	4.31	RNR2	RNR2	rRNA	0	0	0	BT:93.1,PT:100,OA:93.1
YP_003024034.1	RefProt	Homo sapiens	98	10.74	6.2	ND4L	ND4L	CDS	0	0	12	DR:55.68,MM:66.33,RN:69.15,DM:36.67,BT:73.47,PT:98.98,OA:76.53

ANTIBODY PRODUCTION (antigen in red)

IP_306387	AltProt	Homo sapiens	32	3.66	11.93	ND2	ND2	CDS	0	0	0	PT:65.62	**MTKTSPHLNHMPNLSLTKRKPSPHSLNLIHHS**
IP_306389	AltProt	Homo sapiens	33	3.7	9.51	COX1	COX1	CDS	0	0	0		**MPNAPLRLIRPNHSSPTSPISPSPSCWHHYTTN**
IP_306398	AltProt	Homo sapiens	35	3.82	6.5	ND4	ND4	CDS	0	0	0	PT:85.71	**MSSSKPHLSPPWLSSPDEATSQNAWTQAHTSYSTP**
IP_306403	AltProt	Homo sapiens	49	5.22	4.14	CYTB	CYTB	CDS	0	0	0	PT:74.29	**MAESSATFTPMAPQYSLSASSYTSGEAYITDHFSTQKPETSALSSCLQL**

Table S3. Identification of mitochondrial smORFs and altORFs - MS approach and sequences selected for antibody production.

Legend
Tables: PepQuery outputs

Prot_Accession Accession number for the protein (unique ID)
Description Accession number for the protein (output by pepquery)
Prot_Seq Protein sequence
PepQuery_hits Number of peptide spectrum matches (PSMs) reported by PepQuery = number of PSMs with higher score than with any reference peptides **without** PTMs **Confident_hits** Number of confident PSMs reported by PepQuery = number of PSMs with higher score than with any reference peptides **with or without any PTMs** **Best_Score** Score of the best confident PSM
pvalue *p*-value of the best confident PSM
Peptide_Sequence Peptide sequence yielding the best PSM
Peptide_Check_Mass Theoretical mass of the peptide *(may vary from experimental mass based on precursor charge, PTMs, and error)*
Exp_Pep_Mass Observed mass of the peptide (experimental)
Error_mass_ppm Error of the peptide mass in ppm
Validation **#N/A** if no confident PSMs; **No** if no confident PSMs with an error less than |4,5| ppm; **Yes** if confident PSM with an error less than |4,5| ppm

Best PSM for each protein

Prot_Accession	Description	Prot_Seq	PepQuery_hits	Confident_hits	Best_Score	pvalue	Peptide_Sequence	Peptide_Check_Mass	Exp_Pep_Mass	Error_mass_ppm	Validation
SB_0001	SB_0001	IWYFRLGGMHAMALRDAGAGAPYVAVSVFDSCLILLFIAPTFNITGEHTY	37	4	18.28136369	0.001020408163	LGGMHAMALR	1055.536813	1215.632296	1.251744129	Yes
SB_0002	SB_0002	MMMTIECLHSHFPHRHHNKKFPPNPPSPASGHST	31	0	0	#N/A	#N/A	#N/A	#N/A	#N/A	#N/A
SB_0003	SB_0003	IIFPSHSHTTNLINTTPAHPTQHTHTAANPMPRTNQTPKTPPTVYYVAYLLKAMHWKCLDGLTSPHKQMGLVLAFLLALSKITHASIPVPVSSPSKSPRSKGTSIKHAAMQLKTLSLATPPRETAVINL	52	3	21.5925048	0.008743169399	TLSLATPPR	954.5498034	1098.655792	-3.548999013	Yes
SB_0004	SB_0004	MKLKLTWVVKSSWHKMDYESGNMSEHTMAKTQGIRYPTMLSPKPQQLNQQNCSPEHYPQLKTQRTWRCMSL	23	1	12.50724417	0.000999000999	MDYESGFNMSEHTMAKTQTGIR	2533.103525	2869.269804	7.891735204	No
SB_0005	SB_0005	MPPSSANPDEGYKVSASTHVKTLQGVAHEVARNGHFLPQKTTMALMKLKGRRWI	111	8	31.85069597	0.001998001998	TLGQGVAHEVAR	1236.657452	1380.764859	-3.848103668	Yes
SB_0006	SB_0006	IYMEETSRNMVSVLESALGRTRV	55	4	22.97199697	0.000999000999	IYMEETSRNMVSVLESALGR	2284.119098	2572.347638	-9.47025908	No
SB_0007	SB_0007	MVGRFMGRGDKPTEPGDSWLSKMES	54	12	27.52984605	0.001998001998	MVGRFMGRGDKPTEPGDSWLSK	2450.183426	2914.478541	0.3292530069	Yes
SB_0008	SB_0008	ILLRMSLRQIKTLNWQLTAQYLQSTNKSLLPSLSTQHRHAHKERLKKVKGTRQILPRLFTKNITSSITSIRGTACPVTHV	43	1	23.99545008	0.000999000999	LFTKNITSSITSIR	1579.893313	1868.103082	-2.998073063	Yes

SB_0009	SB_0009	MGTCMNGSTRVQLSLTFNQWNWPAREEAGMTQQDEKTLWSFNLLMQTVPNKPTGPKLPNLH	20	0	0	#N/A	#N/A	#N/A	#N/A	#N/A	#N/A
SB_0010	SB_0010	IDPMTWPTEQVTLGMTAQSYSRVHINNRVYDLDVGSGHPDGAAAIKGSFVQRLKSYVIWVQTGVIQVGFYLXSNSSLYERTREMRPTSQSAFPRKWYHLNLVLYPHPPKNRVC	53	1	20.4256365	0.000999000999	EMRPTSQSAFPR	1405.6772	1565.764758	6.03635534	No
SB_0011	SB_0011	IPLLNNMPMANLLLLIVPILIAMAFLMLTERKILGYMQLRKGPNVVGPYGLLQPFADAMKLFTKEPLKPATSTITLYITAPTLALTIALLLWTPLPMPNPLVNLNLGLLFILATSSLAVYSILWSGWASNSNYALIGALRAVAQTISYEVTLAIILLSTLLMSGSFNLSTLITTQEHLWLLLPSWPLAMMWFISTLAETNRTPFDLAEGESELVSGFNIEYAAGPFALFFMAEYTNIIMMNTLTTTIFLGTTYDALSPELYTTYFVTKTLLLTSLFLWIRTAYPRFRYDQLMHLLWKNFLPLTLALLMWYVSMPITISSIPPQT	97	75	90.43099497	0.000999000999	KGPNVVGPYGLLQPFADAMK	2101.102977	2533.421241	-4.745353471	No
SB_0012	SB_0012	MQNPPHSSPHSSPLPRYSYLSPLLY	8	0	0	#N/A	#N/A	#N/A	#N/A	#N/A	#N/A
SB_0013	SB_0013	IYSPMLHSAILPHPHWCSPTVDYSLQTTKTLEHYTYYSAHELES	1	0	0	#N/A	#N/A	#N/A	#N/A	#N/A	#N/A

SB_0014	SB_0014	MTLSKLNYRLNP MYLNGTCSASRS TRRYFPYHRRAY HLSWSRPHNHFP YLLPSPVCPFPNT HNKTN	38	0	0	#N/A	#N/A	#N/A	#N/A	#N/A	#N/A
SB_0015	SB_0015	MVLNLRVHRLRR TNLQLLHTSPIIPR TRRPATPWRWQS SSTPDWSPHSYN NYITRRLALMSC PHIRLKNRCNSRT SKPNHFHRYTTG GMLRSMLWNLW SKPQFHAHRPRIN SPKNLWNRARIY PMAPPLPPLEPTV KLT	49	2	19.26859725	0.008849557522	NLWNRAR	928.4991084	1072.603732	-2.365401482	Yes
SB_0016	SB_0016	IKRTNTSLQWNA PTKYYRMAHHN YPHTPYTIPHHPT KNIKHKLPPTSLT KAH KNKKL	29	0	0	#N/A	#N/A	#N/A	#N/A	#N/A	#N/A
SB_0017	SB_0017	MNENLFASFIAPT ILGLPAAVLIILFP PLLIPTSKYLINN RLITTQQWLIKLT SKQMMTMHNTK GRTWSLMLVSLII FIATTNLLGLLPH SFTPTTQLSMNL AMAIPLWAGTVI MGFRSKIKNALA HFLPQGTPTPLIP MLVIIETISLLIQP MALAVRLTAN ITAGHLLMHLIGS ATLAMSTINLPST LIIFTILILLTILEIA VALIQAYVFTLL VSLYLHDNT	130	89	51.46183017	0.001020408163	LITTQQWLIK	1242.733572	1530.93992	-1.428598097	Yes
SB_0018	SB_0018	ILPSYKPQSTSSLP SPFPTASTAQHFL	0	0	0	#N/A	#N/A	#N/A	#N/A	#N/A	#N/A
SB_0019	SB_0019	MWFDYFCMSPSI DEGLTLLV	2	0	0	#N/A	#N/A	#N/A	#N/A	#N/A	#N/A
SB_0020	SB_0020	ITTTDMTFQKTHN LNQHNHPQPNY	9	1	21.06164271	0.00515995872	ITTTDMTFQK	1184.574696	1488.764668	6.110094777	No

SB_0021	SB_0021	IHSHRTNHILYLL RNHTYPHLGYHH PMRQPARTPERR HMLPILHPSRLPS PTHRTNLHSQHP RLTKHSTTHSHCP RTIKLLSQQLNM TSLHNSFYSKDTS LRTPLMTP	37	0	0	#N/A	#N/A	#N/A	#N/A	#N/A	#N/A
SB_0022	SB_0022	MRHNYNKLHLPT TNRPKIAHCMLF NQPHSPRSNSHS HPNPLKLHRRSH SHNRPRAYILITIL PSKLKLRTHSQS HHNPLSRTSNSTP TNSFLM TSSKPR	62	11	19.17011656	0.002018163471	LHLPTTNRPK	1175.677457	1463.877578	2.758316996	Yes
SB_0023	SB_0023	IHTRKHPHVHTPI PHSPPIPQPRHHY RVFLL	42	1	14.03856455	0.002421307506	HHYRVFLL	1083.597754	1227.709476	-7.84342575	No

SB_0024	SB_0024	ILVQLQMKVMT<br>MHTTMTTLTLTS<br>LIPPILTTLVNPNK<br>KNSYPHYVKSIV<br>ASTFIISLFPTTMF<br>MCLDQEVIISNW<br>HWATTQTTQLSL<br>SFKLDYFSMMFIP<br>VALFVTWSIMEF<br>SLWYMNSDPNIN<br>QFFKYLLIFLITM<br>LILVTANNLFQLF<br>IGWEGVGIMSFLL<br>ISWWYARADAN<br>TAAIQAILYNRIG<br>DIGFILALAWFIL<br>HSNSWDPQQMA<br>LLNANPSLTPLLG<br>LLLAAAGKSAQL<br>GLHPWLPSAMEG<br>PTPVSALLHSSTM<br>VVAGIFLLIRFHP<br>LAENSPLIQTLTL<br>CLGAITTLFAAVC<br>ALTQNDIKKIVAF<br>STSSQLGLMMVT<br>IGINQPHLAFLHIC<br>THAFFKAMLFMC<br>SGSIIHNLNNEQD<br>IRKMGGLLKTMP<br>LTSTSLTIGSLAL<br>AGMPFLTGFYSK<br>DHIIETANMSYTN<br>AWALSITLIATSL<br>TSAYSTRMILLTL<br>TGQPRFPTLTNIN<br>ENNPTLLNPIKRL<br>AAGSLFAGFLITN<br>NISPASPFQTTIPL<br>YLKLTALAVTFL<br>GLLTALDLNYLT<br>NKLKMKSPLCTF<br>YFSNMLGFYPSIT<br>HRTIPYLGLLTSQ<br>NLPLLLLDLTWL<br>EKLLPKTISQHQI<br>STSIITSTQKGMIK<br>LYFLSFFFPLILTL<br>LLIT	113	63	76.17222727	0.000999000999	FPTLTNINENNPTL<br>LNPIKR	2308.253868	2596.46168	-1.396327951	Yes

SB_0025	SB_0025	IPPSNLNYNMYTN KQCSTSNYY	41	0	0	#N/A	#N/A	#N/A	#N/A	#N/A	#N/A
SB_0026	SB_0026	MIMQSPRTNRILP NQPWPLSFMNYS ASYTIKVYHNHH PIMLFHPQHQSY LHR	34	0	0	#N/A	#N/A	#N/A	#N/A	#N/A	#N/A
SB_0027	SB_0027	MTSPKIQNNNTP DHTANNQY	6	0	0	#N/A	#N/A	#N/A	#N/A	#N/A	#N/A
SB_0028	SB_0028	MHHYSRTDYNH DQWYEKPSLYFN YKNTNDPNTQN	2	0	0	#N/A	#N/A	#N/A	#N/A	#N/A	#N/A
SB_0029	SB_0029	IHRPPHPIQHLRM MKLRLTPWRLPD PPNHHRTIPSHAL LTRRLNRLFINRP HHSRRKLWLNHP LPSRQWRLNILY LPLPTHRARPML RIISLLRNLKHRH YPPACNYSNSLH RLCPPVRPNIILR GHSNYKLTIRHP MHWDRPSSMNL RRLLSRQSHPHTI LYLSLHLALHYC SPSNTPPPILARN GIKQPPRNHLPFR	121	2	13.82029738	0.004424778761	LRLTPWR	940.5606422	1084.660857	1.728571636	Yes
SB_0030	SB_0030	INTILTRPPRRPR QLYPSQPLKHPS PHQARMMFPIRL HNSPIRP	30	0	0	#N/A	#N/A	#N/A	#N/A	#N/A	#N/A
SB_0031	SB_0031	ITIHPHPSNNPHPP YIQTTKHNISPTK PITLLTPSRRPPHS NLNRRTTSKLPFY HHWTSSIRTMLH NNPNPNTNYLPN WKQNTQMGLSL	49	1	16.8933154	0.003144654088	RPPHSNLNR	1089.579146	1233.689229	-6.478610557	No
SB_0032	SB_0032	MHQSCKPEMKTF FQGQIREKVFNST ISTQS	20	2	13.83337106	0.003003003003	MHQSCKPEMK	1217.535493	1722.853313	2.770027249	Yes

SB_0033	SB_0033	MYFVHYCQPPW MLYGTMNTWPP VVHKNPIHIKTPS PCLQASTAINPQL SHINCNSKATPHP LGYQQTYPPLTV HST	22	0	0	#N/A	#N/A	#N/A	#N/A	#N/A	#N/A
SB_0034	SB_0034	MAHYSQIPSRPH GWPPSDRGPLTT ILREINIPHKSAT LLAPGP	10	0	0	#N/A	#N/A	#N/A	#N/A	#N/A	#N/A
SB_0035	SB_0035	MSQYLSLIPASSY YLSHLRSMLQAN MLTKVC	14	0	0	#N/A	#N/A	#N/A	#N/A	#N/A	#N/A
SB_0036	SB_0036	MTKNFHQTPPPP LLATALKHISAK PQKQRTLTPA	3	0	0	#N/A	#N/A	#N/A	#N/A	#N/A	#N/A
SB_0037	SB_0037	MLLISSMQPPPIL PSTHTPLLTPYPE PTKPQRHPPQFM	0	0	0	#N/A	#N/A	#N/A	#N/A	#N/A	#N/A
SB_0038	SB_0038	MQASPFQWVHP LNHHDQKEQAS STQQCSSKRLA	12	0	0	#N/A	#N/A	#N/A	#N/A	#N/A	#N/A
SB_0039	SB_0039	MLTPGLVNFVPA TAVTRLTQVNRS RRKECFRSPPPQ	10	0	0	#N/A	#N/A	#N/A	#N/A	#N/A	#N/A
SB_0040	SB_0040	MEIETWRNRYS TARERWKIMTK HNMARTNPYTF CMMN	33	0	0	#N/A	#N/A	#N/A	#N/A	#N/A	#N/A
SB_0041	SB_0041	MTLQGEPKLRPP KPDELPKNS	10	0	0	#N/A	#N/A	#N/A	#N/A	#N/A	#N/A
SB_0042	SB_0042	IKKAFKLNTHYL KNPKHMTELLTP NWTNLSPYRRTN VSMSNMKTFSSA	71	8	16.0339017	0.008743169399	TNVSMSNMK	1010.452479	1330.657952	-8.639537899	No
SB_0043	SB_0043	MAPRGFSCLLLL TSEIDLPVKRRA	4	0	0	#N/A	#N/A	#N/A	#N/A	#N/A	#N/A
SB_0044	SB_0044	IKNFGWGDLGAE PNLRAVHAKTSP VKANYYTQLIQ	20	1	23.17994279	0.000999000999	NFGWGDLGAEPN LR	1544.737155	1688.850186	-6.473704492	No
SB_0045	SB_0045	ILESMSTMGFTTS MLDQDIPMVQPL LKVRLFND	1	0	0	#N/A	#N/A	#N/A	#N/A	#N/A	#N/A

SB_0046	SB_0046	MAEPGNRMKLK TLQSEVQFLFLTT YPWPTSYSSLYPF	18	3	13.93237678	0.008869179601	MAEPGNR	773.3490056	917.4533302	-2.447302268	Yes
SB_0047	SB_0047	MSPYPLQSPAFPL KPKKYVW	0	0	0	#N/A	#N/A	#N/A	#N/A	#N/A	#N/A
SB_0048	SB_0048	ISRTMRIEPIPENP KFSVPPITPHPKV RSAK	10	0	0	#N/A	#N/A	#N/A	#N/A	#N/A	#N/A
SB_0049	SB_0049	IFYLSRPRNKHAS FYSSSNQKNKPSF HRSCHQVFPHAS NRIHNPSNSYPLQ QYTLRTMNHNQ YYQSMLIINNHNS YSNKTRNSPLSLL SPRGYPRHPSDIR PASSHMTKTSPH LNHMPNLSLTKR KPSPHSLNLIHHS RQLRWIKPNPAT QNLSMLLNYPHR MNNSSSTVQP	122	4	12.05265959	0.007658643326	IFYLSRPR	1050.59742	1338.79671	3.632896529	Yes
SB_0050	SB_0050	IPTTQLKLQHHDP TTISHLKQANMT NTLNSIHPPLPRR PAPANRLFAQMG HYRRIHKKQ	41	1	20.32219288	0.000999000999	QANMTNTLNSIHP PLPR	1902.973367	2047.067215	4.036097747	Yes
SB_0051	SB_0051	MTVWTYKTHPIP PHTHRPYHATPT YLPFYTNNLMEI	0	0	0	#N/A	#N/A	#N/A	#N/A	#N/A	#N/A
SB_0052	SB_0052	ISVTAKDCKTPLC INWTQISHFN	2	0	0	#N/A	#N/A	#N/A	#N/A	#N/A	#N/A
SB_0053	SB_0053	MKITSELVKRGL TPVFRFTVQCFTQ PFYLTPTDVRRPL TILYKPQRHWNT MPIIRRMSWSPRH SSKPPYSSRAGPA RQPSR	31	2	17.19087616	0.001001001001	HWNTMPIIRR	1322.702959	1466.810724	-3.863706822	Yes

SB_0054	SB_0054	ICNNLLHSNTHH NRRLWQLTSSPN NRCPRYGVSPHK QHKLLTLTSLSPT PARICYSGGRSRN RLNSLPSLSRELL PPWSLRRPNHLL LTPSRCLLYLRG HQFHHNNYQYK TPCHNPMPNAPL RLIRPNHSSPTSPI SPSPSCWHHYTT NRPQPQHHLLRP RRRRRPHSMPTPI LIFRSPWSLYSYP TRLRNNLPYCNL LLRKKRTIWMHR YGLSYDINWLPR VYRVSTPYIYSRN RRRHTSMFHLRY HNHRYPHRRQSI	156	8	27.54786054	0.001154734411	ELLPPWSLR	1109.623299	1253.713095	9.8081714	No
SB_0055	SB_0055	MICCSALSPRIHL SFHRRWPDWHCI SKLITRHRTTRHV LRCSPLPLCPINR SCICHHRRLHSLI SPILRLHPRPNLR QNPFHYHIHRRK SNFLPTTLSRPIR NAPTLLGLPRCM HHMKHPIICRLIH FSNSSNINNFHDL RSLRFEAKSPNSR RTLHKPGVTMW MPPTLPHIRRTR MHKI	99	8	26.39510071	0.000999000999	TTRHVLRCSPLPL CPINR	2075.124397	2333.278658	-3.949405601	Yes

SB_0056	SB_0056	MAHAAQVGLQD<br>ATSPIMEELITFH<br>DHALMIIFLICFL<br>VLYALFLTLTTK<br>LTNTNISDAQEM<br>ETVWTILPAIILV<br>LIALPSLRILYMT<br>DEVNDPSLTIKSI<br>GHQWYWTYEYT<br>DYGGLIFNSYML<br>PPLFLEPGDLRLL<br>DVDNRVVLPIEA<br>PIRMMITSQDVL<br>HSWAVPTLGLKT<br>DAIPGRLNQTTFT<br>ATRPGVYYGQCS<br>EICGANHSFMPIV<br>LELIPLKIFEMGP<br>VFTL	779	656	76.27685888	0.000999000999	MMITSQDVLHSW<br>AVPTLGLK	2226.154024	2514.353794	1.755039781	Yes
SB_0057	SB_0057	MPQLNTTVWPT<br>MITPMLLTLFLIT<br>QLKMLNTNYHLP<br>PSPKPMKMKNY<br>NKPWEPKWTKIC<br>SLHSLPPQS	328	277	64.10598268	0.000999000999	MLNTNYHLPPSPK<br>PMK	1866.948398	2299.260345	-2.484358335	Yes
SB_0058	SB_0058	MTPNRGPLSPPN<br>DLRPSHVISLPLH<br>NAPHTRPTNQHT<br>NHMPMMARCNT<br>RKHMPRPPHTTC<br>PKRPSMRDNPIY<br>YLRSFFLRRIFLS<br>LLPLQPSPYPPIR<br>RALAPNRHHPAK<br>SPRSPTPKHIRITR<br>IRSINHLSSP	35	2	24.59374409	0.000999000999	RPSMRDNPIYYLR	1679.856552	1968.066962	-3.172199407	Yes
SB_0059	SB_0059	IFCSHRLPRTSRH<br>YWLNFPHYLLHP<br>PTNISLYIQTSLW<br>LRSRRLMLAFCR<br>CGLTISVCLHLL<br>MRVLLF	10	0	0	#N/A	#N/A	#N/A	#N/A	#N/A	#N/A
SB_0060	SB_0060	ILTTTTQRLHRKI<br>HPLRVRLRPYIPR<br>PRPFLHKILLSSY<br>YLLIIWSRNCPPF<br>TPTM PTNN	18	2	21.63645375	0.001005025126	ILTTTTQRLHR	1338.773144	1482.86694	5.600029957	No
SB_0061	SB_0061	INHHPSPKSGLWV<br>TTKRIRLNRIGM	7	0	0	#N/A	#N/A	#N/A	#N/A	#N/A	#N/A

SB_0062	SB_0062	IYHLTSRNTSMSLTPHILPTMPRRNNTIAVHYSYSHNPQHPLPLSQYCAYCHTSLCRLRSSGGPSPTSLNLQHMWPRLRT	64	0	0	#N/A	#N/A	#N/A	#N/A	#N/A	#N/A
SB_0063	SB_0063	MLKLIVPTIMLLPLTWLSKKHMIWINTTTHSLIISIIPLLFFNQINNNLFSCSPTFSSDPLTTPLLMLTTWLLPLTIMASQRHLSSEPLSRKKLYLSMLISLQISLIMTFTATELIMFYIFFETTLIPTLAIITRWGNQPERLNAGTYFLFYTLVGSLPLLIALIYTHNTLGSLNILLLTLTAQELSNSWANNMWLAYTMAFMVKMPLYGLHLWLPKAHVEAPIAGSMVLAAVLLKLGGYGMMRLTLILNPLTKHMAYPFLVLSLWGMIMTSSICLRQTDLKSLIAYSSISHMALVVTAILIQTPWSFTGAVILMIAHGLTSSLLFCLANSNYERTHSRIMILSQGLQTLLPLMAFWWLLASLANLALPPTINLLGELSVLVTTFSWSNITLLLTGLNMLVTALYSLYMFTTTQWGSLTHHINNMKPSFTRENTLMFMHLSPILLLSLNPDIITGFSSCKYSLTKTSDCESDNRGLRPLIYRESSQELLTHAPMSNNMAFSTFKG	87	8	84.24539063	0.000999000999	AHVEAPIAGSMVLAAVLLK	1889.080786	2177.288583	-1.661575297	Yes
SB_0064	SB_0064	MPTQQPFKQSYTTVSAMSVSSSP	8	0	0	#N/A	#N/A	#N/A	#N/A	#N/A	#N/A

SB_0065	SB_0065	ITMYTPTNNVQPV<br>TTTNQRP	0	0	0	#N/A	#N/A	#N/A	#N/A	#N/A	#N/A
SB_0066	SB_0066	IIQLPTLLKFTTTT<br>TPSYSFTHSTNPT<br>SIANPTKTLTKTS<br>TPDPHASGYSSM<br>AIAVVYPKTTIIPP<br>K	16	0	0	#N/A	#N/A	#N/A	#N/A	#N/A	#N/A
SB_0067	SB_0067	MGEGLEENPTNPI<br>TKPTLNRNKAYII<br>ILARTTTTTNDM<br>KNHRCISTTRTP<br>MTPMRKTNPLM<br>KLINHSFIDLPTPS<br>NISAWWNFGSLL<br>GACLILQITTGLF<br>LAMHYSPDASTA<br>FSSIAHITRDVNY<br>GWIIRYLHANGA<br>SMFFICLFLHIGR<br>GLYYGSFLYSET<br>WNIGIILLLATMA<br>TAFMGYVLPWG<br>QMSFWGATVITN<br>LLSAIPYIGTDLV<br>QWIWGGYSVDS<br>PTLTRFFTFHFILP<br>FIIAALATLHLLF<br>LHETGSNNPLGIT<br>SHSDKITFHPYYT<br>IKDALGLLLFLLS<br>LMTLTLFSPDLLG<br>DPDNYTLANPLN<br>TPPHIKPEWYFLF<br>AYTILRSVPNKLG<br>GVLALLLSILILA<br>MIPILHMSKQQS<br>MMFRPLSQSLYW<br>LLAADLLILTWIG<br>GQPVSYPFTIIGQ<br>VASVLYFTTILIL<br>MPTISLIENKMLK<br>WACPCSMN	63	7	21.38188719	0.002212389381	KTNPLMK	830.4683856	1262.772811	1.41407854	Yes
SB_0068	SB_0068	ILCSFMGKQIWV<br>PPKYWLTHQQPL<br>CISYITASHHEYC<br>TVP	13	4	16.55336317	0.001092896175	ILCSFMGK	897.4452082	1242.673333	-2.022001366	Yes
SB_0069	SB_0069	MKTQSTSKPPPH<br>AYKQVQQSTLNY<br>HTSTATPKPPLTH	5	0	0	#N/A	#N/A	#N/A	#N/A	#N/A	#N/A

SB_0070	SB_0070	ITVKSLLVPMDDP PQMGVPWPPSSV KSMSRTRVLLSS LRAHNTWG	3	0	0	#N/A	#N/A	#N/A	#N/A	#N/A	#N/A
SB_0071	SB_0071	INHSRELSMHLV FSSGGYARDSIA RRWSRSTLCRSI CLWFLPHPIIYRT YVQYYRRTYLL KCVN	60	0	0	#N/A	#N/A	#N/A	#N/A	#N/A	#N/A
SB_0072	SB_0072	MLVGHNNNNW MSAQPLSTQTS	0	0	0	#N/A	#N/A	#N/A	#N/A	#N/A	#N/A
SB_0073	SB_0073	ISTKPPLPRFWPQ HLNTSLPNPKNK EP	4	0	0	#N/A	#N/A	#N/A	#N/A	#N/A	#N/A
SB_0074	SB_0074	ISNFIFWRYALLT VTPQLTHYFPLPL PYY	2	0	0	#N/A	#N/A	#N/A	#N/A	#N/A	#N/A
SB_0075	SB_0075	MEAGVKSVLDHP LPNKAKTHLSCK KLQLTQNRLRKW L	5	0	0	#N/A	#N/A	#N/A	#N/A	#N/A	#N/A
SB_0076	SB_0076	MNPDQPHHLLLS LYTAIFSKPWWR LQSKRKYPRKDV RSRCSPWGGKK WATFSTPENYDS PYET	18	0	0	#N/A	#N/A	#N/A	#N/A	#N/A	#N/A
SB_0077	SB_0077	ICPQNPLNPLVNL TVSPKRNSSLDTR KKPCRESKKFNT HSRPKSSHQLRK RSSSTPTT	17	0	0	#N/A	#N/A	#N/A	#N/A	#N/A	#N/A
SB_0078	SB_0078	INSPMSTINQQVII TLTVNPTQACS	0	0	0	#N/A	#N/A	#N/A	#N/A	#N/A	#N/A
SB_0079	SB_0079	MITCSLNRDLYE WLHEGSAVSYF	10	1	12.12041551	0.009836065574	MITCSLNR	936.4520848	1153.56304	6.508987843	No
SB_0080	SB_0080	ISVGATSEQNPTS EQYMLRLHQSKR TTMLNWSNNLTN G TSYPRDNSAILF	47	0	0	#N/A	#N/A	#N/A	#N/A	#N/A	#N/A
SB_0081	SB_0081	IKVLRDLSSDRS NPGRFLSXFKFLP VRKDKRNKAYF TKRLP P	33	1	22.79775127	0.003058103976	RNKAYFTKR	#N/A	1614.978081	-6.018171773	No
SB_0082	SB_0082	MMSSQLSIMPTP TQEQGLLRWQS PVIA	19	0	0	#N/A	#N/A	#N/A	#N/A	#N/A	#N/A

SB_0083	SB_0083	IPNAYRTKNSRLYTTTQRPQRCRPLRATTTLRWRHKTLHQRAPKTRHIYHHPLHHRPDLSSHHRSSTMNPPPHTQPPGQPQPRPPIYSSHL	34	0	0	#N/A	#N/A	#N/A	#N/A	#N/A	#N/A
SB_0084	SB_0084	ITPAIMTLGHNMIYLHTSRDQPNPLRPCRRGVRTSLRLQHRMRRRPLRPILHSRMHKHYYNKHPHHYNLPRNNMWRTLPWTLHNMFCHQDPTSNLPVLMNSNSMPPIPLRPTHTPPMKKLPTTHPSITYMMCLHTHYNLQHSPSNLRNMSDKRVTLME	101	4	24.34214933	0.000999000999	NMSDKRVTLME	1322.632225	1626.841966	-6.55855142	No
SB_0085	SB_0085	INPLAQPVIYSTIFAGTLITALSSHWFFTWVGLEMNMLAFIPVLTKKMNPRSTEAAIKYFLTQATASMILLMAILFNNMLSGQWTMTNTTNQYSSLMIMMAMAMKLGMAPFHFWVPEVTQGTPLTSGLLLLTWQKLAPISIMYQISPSLNVSLLLTLSILSIMAGSWGGLNQTQLRKILAYSSITHMGWMMAVLPYNPNMTILNLTIYIILTTTAFLLLNLNSSTTTLLLSRTWNKLTWLTPLIPSTLLSLGGLPPLTGFLPKWAIIEEFTKNNSLIIPTIMATITLLNLYFYLRLIYSTSITLLPMSNNVKMKWQFEHTKPTPFLPTLIALTTLLLPISPFMLMI L	68	17	30.98510606	0.001048218029	WAIIEEFTK	1135.591332	1423.796681	-0.8391981488	Yes

SB_0086	SB_0086	MFADRWLFSTNH KDIGTLYLLFGA WAGVLGTALSLL IRAELGQPGNLLG NDHIYNVIVTAH AFVMIFFMVMPI MIGGFGNWLVPL MIGAPDMAFPRM NNMSFWLLPPSL LLLLASAMVEAG AGTGWTVYPPLA GNYSHPGASVDL TIFSLHLAGVSSIL GAINFITTIINMKP PAMTQYQTPLFV WSVLITAVLLLLS LPVLAAGITMLLT DRNLNTTFFDPA GGGDPILYQHLF WFFGHPEVYILI LPGFGMISHIVTY YSGKKEPFGYMG MVWAMMSIGFL GFIVWAHHMFTV GMDVDTRAYFTS ATMIIAIPTGVKV FSWLATLHGSNM KWSAAVLWALG FIFLFTVGGLTGI VLANSSLDIVLHD TYYVVAHFHYVL SMGAVFAIMGGF IHWFPLFSGYTLD QTYAKIHFTIMFI GVNLTFFPQHFL GLSGMPRRYSDY PDAYTTWNILSS VGSFISLTAVM LMIFMIWEAFAS KRKVLMVEEPSM NLEWLYGCPPPY HTFEEPVYMKSR QKRKESNPPKLV SSQPHGLHDFFK KVLEKPFHNFVK VKL	151	91	90.04728257	0.000999000999	VLMVEEPSMNLE WLYGCPPPYHTF EEPVYMK	3727.711022	4088.940668	-2.215073813	Yes

SB_0087	SB_0087	IMTNPENQNERKSVRFIHCPHNPRPTRRSTDHSISPSIDPHLQMSHQQPTNHHPTMTNQTNLKTNDNHTQH	31	0	0	#N/A	#N/A	#N/A	#N/A	#N/A	#N/A
SB_0088	SB_0088	IYTNHPTIYKPSHGHPLMSGHSDYRLSL	3	0	0	#N/A	#N/A	#N/A	#N/A	#N/A	#N/A

SB_0089	SB_0089	MMTHQSHAYHM VKPSPWPLTGAL SALLMTSGLAM WFHFHSMTLLML GLLTNTLTMYQ WWRDVTRESTY QGHHTPPVQKGL RYGMILFITSEVF FFAGFFWAFYHS SLAPTPQLGGHW PPTGITPLNPLEV PLLNTSVLLASG VSITWAHHSLME NNRNQMIQALLI TILLGLYFTLLQA SEYFESPFTISDGI YGSTFFVATGFH GLHVIIGSTFLTIC FIRQLMFHFTSKH HFGFEAAAWYW HFVDVVWLFLYV SIYWWGSYSFSM NSTVNFQLTSFD NIQKRVMNFALIL MINTLLALLLMII TFWLPQLNGYME KSTPYECGFDPM SPARVPFSMKFFL VAITFLLFDLEIAL LLPLPWALQTTN LPLMVMSSLLLIII LALSLAYEWLQK GLDWTELVYSLN KTNDFDSLNYDN HIYQMPLIYMNI MLAFTISLLGML VYRSHLMSSLLC LEGMMLSLFIMA TLMTLNTHSLLA NIVPIAMLVFAAC EAAVGLALLVSIS NTYGLDYVHNLN LLQC	111	69	55.11127489	0.000999000999	ESTYQGHHTPPVQ K	1607.769182	1895.97657	-1.701906999	Yes
SB_0090	SB_0090	MSSSKPHLSPPWL SSPDEATSQNAW TQAHTSYSTP	0	0	0	#N/A	#N/A	#N/A	#N/A	#N/A	#N/A
SB_0091	SB_0091	IVNLTTEAYDPLF TEKAHKNC	2	0	0	#N/A	#N/A	#N/A	#N/A	#N/A	#N/A

SB_0092	SB_0092	MPPCLTTWLSQL LKDNSYPLVLGP KNFGATPNKSNN HAHYYNHPNPDF PNSPHPYHPR	28	4	20.47544064	0.001998001998	DNSYPLVLGPK	1201.634257	1489.846087	-5.150590851	No
SB_0093	SB_0093	MPPLCKIHCRIHL YYQSLPHNNIHVP RPRSYYLELTLSH NPNNPALPKLQT RLLLHNIHPCSIV RYMVHHRILTVM YKLRPKH	33	0	0	#N/A	#N/A	#N/A	#N/A	#N/A	#N/A
SB_0094	SB_0094	IPTVHRLRGRRN YILLAHQLMMRP SRCQHSSHSSNP MQPYRRYRFHPR LSMIYPTLQLMRP TTNSPSKR	40	0	0	#N/A	#N/A	#N/A	#N/A	#N/A	#N/A
SB_0095	SB_0095	IRSPPLTPLSHRRP HPSLSPTPLKHYS CSRNLLTHPLPPP SRK	14	0	0	#N/A	#N/A	#N/A	#N/A	#N/A	#N/A
SB_0096	SB_0096	MLRRYHHSVRSS LRPYTKWHQKNR SLLHFKSTRTHNS YNRHQPTTPSIPA HLYPRLLQSHTIY VLRVHHPQP	14	0	0	#N/A	#N/A	#N/A	#N/A	#N/A	#N/A
SB_0097	SB_0097	ISRNTFPHRFLLQ RPHHRNRKHIMH KRLSPIYYSHRYL PDKRL	24	1	17.01163818	0.004842615012	NRKHIMHK	1062.586873	1494.885741	4.915416916	No
SB_0098	SB_0098	MTYSPEQSQLQY MHQQTMFNQ	4	0	0	#N/A	#N/A	#N/A	#N/A	#N/A	#N/A
SB_0099	SB_0099	MMWKTIVVFQL QEHQWPQYAKL TP	2	0	0	#N/A	#N/A	#N/A	#N/A	#N/A	#N/A
SB_0100	SB_0100	IMAESSATFTPM APQYSLSASSYTS GEAYITDHFSTQ KPETSALSSCLQL	0	0	0	#N/A	#N/A	#N/A	#N/A	#N/A	#N/A
SB_0101	SB_0101	IPMKSPSTLTTQS KTPSAYFSSFSP	2	0	0	#N/A	#N/A	#N/A	#N/A	#N/A	#N/A
SB_0102	SB_0102	IDSPINNRYVFRT LLPATMNIVRYH KYLTTCST	11	0	0	#N/A	#N/A	#N/A	#N/A	#N/A	#N/A

SB_0103	SB_0103	MLTSKYSNQPST ITHQLQLQSHPSP TRMPTNLPTLNS T	125	3	11.44115982	0.002997002997	MPTNLPTLNST	1187.585595	1347.676777	4.321420165	Yes
SB_0104	SB_0104	MKPFTVHSTLQS NPFSSPWMTPLR	3	0	0	#N/A	#N/A	#N/A	#N/A	#N/A	#N/A
SB_0105	SB_0105	MYYVLLRVGRFV GILVGEGWLWSC SWCVMVEGWLL YLLVSMGRGFW CGLGFYVLQVVK YLWYRTMFMVA GSNVRNT	151	5	21.9103728	0.000999000999	TMFMVAGSNVR	1211.57907	1355.671422	7.18439158	No
SB_0106	SB_0106	MGESMLGWYPN LLPHERTENSLN	4	0	0	#N/A	#N/A	#N/A	#N/A	#N/A	#N/A
SB_0107	SB_0107	MAYEGCCYSCK QEDNADVSGFW VEKWSVM	5	0	0	#N/A	#N/A	#N/A	#N/A	#N/A	#N/A
SB_0108	SB_0108	IGVKVADDSAMI YVSSDVGDWWK GGWGVWWVVH G	3	0	0	#N/A	#N/A	#N/A	#N/A	#N/A	#N/A
SB_0109	SB_0109	MVFHIIGRGCSPC ENNDVCFVSVEC GFSNGVCGVFF	2	1	13.14643154	0.006053268765	MVFHIIGR	971.5374648	1131.640437	-5.273572206	No
SB_0110	SB_0110	MYYSDGYWGVS WGMGVRGWGL GECFSGVSDGGR IGAVGERVWWG GGCGKL	54	4	28.02515	0.000999000999	IGAVGERVWWGG GCGK	1630.803791	1976.033302	-1.964915008	Yes
SB_0111	SB_0111	ISSSYWLNIVCWC MYCNWDCSGE	0	0	0	#N/A	#N/A	#N/A	#N/A	#N/A	#N/A
SB_0112	SB_0112	MGLRRLCMMCL RFRWCGLWSRNL WGKVFLLMLGC QWWGRLKWEV WFWVVLLFFEYL VHC	23	0	0	#N/A	#N/A	#N/A	#N/A	#N/A	#N/A
SB_0113	SB_0113	MVWLWRRRGYR CAGMLGVVGWC RL	28	0	0	#N/A	#N/A	#N/A	#N/A	#N/A	#N/A
SB_0114	SB_0114	MVLEFGLVGYFL LGGGSGWVRRFL LQL	8	0	0	#N/A	#N/A	#N/A	#N/A	#N/A	#N/A
SB_0115	SB_0115	IGLICLLLLGGGL VVGWGLD	2	0	0	#N/A	#N/A	#N/A	#N/A	#N/A	#N/A

SB_0116	SB_0116	MLRRGWNRYRR YGCMGLLEWLL CWHLLGRIINWW AR RM	32	1	19.24635622	0.008474576271	IINWWAR	957.5184452	1101.610498	9.109554067	No
SB_0117	SB_0117	MLQGWMLWRSS LVWSLGRAGLFG LWLSVSSR	21	1	12.74455536	0.003996003996	AGLFGLWLSVSSR	1391.756098	1535.870039	-7.710232259	No
SB_0118	SB_0118	IETSRARPTAAS QAAKTSMAMGT MLAKREWVLRV MRVAMMNSDSII PSRHSREDMRCE RYTSIPRSEMVN ASMMFM	146	2	20.03051746	0.000999000999	VAMMNSDSIIPSR HSR	1799.877029	1959.982881	-4.506118554	No
SB_0119	SB_0119	MMINKRDDMTIS GRLVVCRAHGR GKRRAISRSNNK KVMATKKNFME KGTRAGDMGSK PHS	64	4	19.56720182	0.002424242424	DDMTISGR	893.3912624	1053.484827	3.257502508	Yes
SB_0120	SB_0120	MIISSKARRVLIIK IKAKFITLFWMLS KLVNWKLTVLF MLKE	24	0	0	#N/A	#N/A	#N/A	#N/A	#N/A	#N/A
SB_0121	SB_0121	METYRNSQTTST KCQYQAAASKPK WCLDVKWNISW RMKQMVRKVEP MMTWSPWKPVA TKNVEP	24	0	0	#N/A	#N/A	#N/A	#N/A	#N/A	#N/A
SB_0122	SB_0122	MPSEMVKGDSKY SEACRRVK	32	1	13.0533318	0.006651884701	YSEACRR	883.3970162	1084.531222	-9.830153414	No
SB_0123	SB_0123	IVMSSAWIIWFRL FSIRLWWAQVID TPDASNTDVFRS GTSRGFSGVMPV GGQCPPNWGVG ARLEW	10	0	0	#N/A	#N/A	#N/A	#N/A	#N/A	#N/A
SB_0124	SB_0124	MNRIIPYRRPFWT GGVWWPWYVLS RVTSRHHWYMV SVLVSRPSMRSV MEWKWNHMAR PEVIRRAERAPVR GHGLGFTMW	113	4	19.15712093	0.000999000999	HHWYMVSVLVSR PSMR	1983.992327	2128.084213	4.805330945	No

SB_0125	SB_0125	IMCCRAGRGLLE<br>VWKRRLGLRRQ<br>RFLG	15	1	25.62950155	0.000999000999	IMCCRAGRGLLEV<br>WK	1733.889112	2152.138023	-3.205659403	Yes
SB_0126	SB_0126	IVGWWLVGCWW<br>DIWRWGSMEGE<br>MEWSVLRRVGL<br>GLWGQWMKRT<br>DFRSFWFSGFVM<br>IFYFYGLWWGR	33	0	0	#N/A	#N/A	#N/A	#N/A	#N/A	#N/A
SB_0127	SB_0127	MEGSSTIRTFRFE<br>AKASQIMKIINIT<br>AVREMNEPTDDR<br>MF HVVYASG	89	4	17.13238898	0.009523809524	IINITAVR	898.5599734	1042.671773	-9.311726812	No
SB_0128	SB_0128	IPDRPRKCCGKKV<br>RFTPMNMMVKW<br>ILA	8	0	0	#N/A	#N/A	#N/A	#N/A	#N/A	#N/A
SB_0129	SB_0129	MSSDEFANTMPV<br>RPPTVKRKMNPR<br>AQSTAADHFMLL<br>PWSVASQLNTLT<br>PVGMAMIMVAE<br>VKYARVSTSIPTV<br>NMWCAHTMNPR<br>KPIDIMAQTMPM<br>YPNGSFFPE	14	0	0	#N/A	#N/A	#N/A	#N/A	#N/A	#N/A
SB_0130	SB_0130	MGSPPPAGSKKV<br>VLRLRSVSSMVM<br>PAARTGRDRRSR<br>TAVIRTDQTKRG<br>VWYWVMAGGF<br>MLMIVVMKLMA<br>PKMEETPARCKE<br>KMVRSTEAPGW<br>E	401	9	31.99770905	0.000999000999	TDQTKRGVWYW<br>VMAGGFMLMIVV<br>MK	2946.478001	3410.753217	6.120421135	No
SB_0131	SB_0131	MADASRSRREGG<br>KSQKLMLFMRG<br>NAMSGAPIIRGTS<br>QLPKPPIMMGIT<br>MKKIITNAWAVT<br>MTL	53	3	11.91927806	0.009900990099	MADASRSR	892.418479	1036.514518	5.830421843	No
SB_0132	SB_0132	MWSLPRRLPGWP<br>SSARMRRLRAVP<br>RTPAHAPNNRYS<br>VPMSLWFVENSQ<br>RSANISGGEVKW<br>LSEALDCKSKDR<br>G	107	5	24.68961083	0.002040816327	WLSEALDCK	1063.500807	1408.724693	1.226049523	Yes

SB_0133	SB_0133	IELQIRRSSFKPA GASPAFFPGGGR SRLKPVD	4	0	0	#N/A	#N/A	#N/A	#N/A	#N/A	#N/A
SB_0134	SB_0134	IKVADLRSVDAE WGFAVLSCYRN	9	0	0	#N/A	#N/A	#N/A	#N/A	#N/A	#N/A
SB_0135	SB_0135	IISMKGEMGRSSV VRAMSVGRNGV GFVCSNCHFIFTL LDMGSSVIEVE	38	1	10.14925663	0.007063572149	IISMKGEMGR	1120.573256	1408.789118	-8.310300416	No
SB_0136	SB_0136	MVAMMVGMMR LLFFVNSSMMAH LGKKPVSGGRPP RERRVDGIKGVS HVSLFQVRDSSR VVVLEFKLSSRN AVVVRMM	90	2	22.19791862	0.002688172043	MVAMMVGMMR	1155.509478	1347.595171	0.8468832668	Yes
SB_0137	SB_0137	MVKLRMVMLGL YGRTAIIHPMWVI EEYAKILRSWVW FNPPQLPAMMDK IERVRRRLTFSEG EIWYMIEMGASF CHVRRSRPDVRG VPWVTSGTQKW KGAIPSFIAMAIM IINDEYWLVVLV MVHCPESMLLKR MAIRRIMDAVAC VRKYLMAASVER GFIFLVRTGMKA SMFISRPTQVKNQ CELSAVMSVPAK MVE	231	23	24.95221347	0.000999000999	SWVWFNPPQLPA MMDK	1945.921851	2250.102287	8.288127825	No
SB_0138	SB_0138	MGCDRWHGEFW ILRDGFDSHSPRN KGV	20	5	18.53962327	0.003144654088	DGFDSHSPR	1016.431152	1160.534518	-1.106494702	Yes
SB_0139	SB_0139	IIYSIKVTLLSDMF LRFEGECWRL	13	0	0	#N/A	#N/A	#N/A	#N/A	#N/A	#N/A
SB_0140	SB_0140	MLGWVVGSFFM GGVWVGRSGIG GMLFEFMRTGRL EVGSWWQNMLC RVQGRVRHMLF LGRL	23	0	0	#N/A	#N/A	#N/A	#N/A	#N/A	#N/A

SB_0141	SB_0141	IRLWRMGRRGLR RIRCWSLRLVRT PLRQGRRGFGWS LL VWR	34	5	24.18824918	0.008869179601	TPLRQGR	826.4773112	970.5764446	3.041597953	Yes
SB_0142	SB_0142	MLMVEWWLGW LHMRLFGLLLA VRRSGRSLSLML TLIRGLSKRLG	10	0	0	#N/A	#N/A	#N/A	#N/A	#N/A	#N/A
SB_0143	SB_0143	MGGLGWGWPG GWVWGGGFMV EERWWELRSGR WCRGWW	12	0	0	#N/A	#N/A	#N/A	#N/A	#N/A	#N/A
SB_0144	SB_0144	MWRVLGALWW RVLWRQRRVVV ARRGLQRWGLC VVVYSLEFFVR	24	1	13.52718761	0.003816793893	QRRVVVAR	982.6148008	1126.70881	7.174189307	No
SB_0145	SB_0145	MPLRLEWVQWG VGGWPWVCC	0	0	0	#N/A	#N/A	#N/A	#N/A	#N/A	#N/A
SB_0146	SB_0146	ITGLCHLNKPCS WVGVGMMLSW DDIIYGGRRFVK	3	0	0	#N/A	#N/A	#N/A	#N/A	#N/A	#N/A
SB_0147	SB_0147	MVVRFDWWSLS MYCSEVGFCSEV APTEIFNAGLVV	0	0	0	#N/A	#N/A	#N/A	#N/A	#N/A	#N/A
SB_0148	SB_0148	MGSSRLAVLCPP LHGQVNFTG	1	0	0	#N/A	#N/A	#N/A	#N/A	#N/A	#N/A
SB_0149	SB_0149	IHYAEGMGVSPC YIMLGYNFSSFPC GTMSIAPGFNFY RLYFIWVNGLAK VVW	6	0	0	#N/A	#N/A	#N/A	#N/A	#N/A	#N/A
SB_0150	SB_0150	MAVYRLSKRW WGWSGFIDYRT GSSRGMWSTAR SFEF	30	0	0	#N/A	#N/A	#N/A	#N/A	#N/A	#N/A
SB_0151	SB_0151	MERLGPNLFVYG VMWARLNIFSVL LWGGKLHKLWG VSLGFGWFGVW G	24	1	11.74932761	0.000999000999	MERLGPNLFVYG VMWAR	2038.028042	2198.13785	-5.814048991	No
SB_0152	SB_0152	IFCYDVCVESGC ADIQLLLLCPTSI N	0	0	0	#N/A	#N/A	#N/A	#N/A	#N/A	#N/A
SB_0153	SB_0153	MMGWGRNQRQ MLRHRVLRLQRL AMLSRAYPPDEN TKCMESSREWL MGW	61	4	16.473808	0.004197271773	MMGWGRNQR	1134.517476	1310.619018	-7.343252657	No

SB_0154	SB_0154	MSDTVHFSYPQVLWARSEESSTLVRDIDFTEDGGQGTPIWGGSSMGTRRDLTVMCYVR	75	3	25.31221444	0.001998001998	DIDFTEDGGQGTPIWGGSSMGTR	2383.038604	2527.126774	5.516739491	No
SB_0155	SB_0155	MYYRWSSIYGTVQYSWWLAVMYEMHSGCWWVSQYLGGTQICFPMKEQRMV	1	0	0	#N/A	#N/A	#N/A	#N/A	#N/A	#N/A
SB_0156	SB_0156	IRILALGANGGVKDFFSDLSLEKGFHLRFTRLVY	22	1	24.23521817	0.001020408163	DFFSDLSLEK	1199.570992	1487.783158	-5.386567532	No
SB_0157	SB_0157	MLQGQAHLSILFSIREMVGIRIRIVVKYSTDATCPMMVKG	99	1	16.12499284	0.003631961259	EMVGIRIR	972.5538426	1132.64155	8.208387675	No
SB_0158	SB_0158	IMLCCLDMWRMGIIARMRMDSNRARTPPSLLGTDRRIV	81	12	20.26730827	0.001048218029	MRMDSNRAR	1135.533854	1295.622706	6.291403817	No
SB_0159	SB_0159	ILSEWEVIPRGLFDPVSCKNRRWSVARAAMMKGKMKWKVKNRVRVGLSTE	33	0	0	#N/A	#N/A	#N/A	#N/A	#N/A	#N/A
SB_0160	SB_0160	IHWTRSVPMYGMADSKFVITVAPQNDIWPHGRT	35	10	18.13464371	0.002997002997	SVPMYGMADSK	1184.520555	1488.711045	5.760069176	No
SB_0161	SB_0161	MKAVAMVASRRMMPMFQVSE	21	2	20.80739396	0.008083140878	AVAMVASRR	959.5334428	1103.624632	9.876398944	No

SB_0162	SB_0162	MARNSPVVIWRI RQAPRSEPKFHH AEMLDGVGRSM NEWLINFIRGLVL RIGVIGVLVVEM QRWFFMSLVVV VVRARMMMYAL FLLSVGLVMGFV GFSSKPSPIYGGL VLIVSGVVGCVII LNFGGGYMGLM VFLIYLGGMMVV FGYTTAMAIEEY PEAWGSGVEVLV SVLVGLAMEVGL VLWVKEYDGV VVVVNFNSVGS WMIYEGEGSGLI REDPIGAGALYD YGRWLVVVTGW TLFVGVYIVIEIA RGNRLCD	88	2	17.30034468	0.000999000999	FHHAEMLDGVGR	1367.640422	1511.729762	8.436715496	No
SB_0163	SB_0163	MGDCAVCDARV ESEYVGEMKCA	15	1	16.17840283	0.005076142132	VESEYVGEMK	1169.527414	1617.826131	1.490421419	Yes
SB_0164	SB_0164	MFEELINVWVW VYMSQWEFYDG PCNEQCYRDEYY GEVV	0	0	0	#N/A	#N/A	#N/A	#N/A	#N/A	#N/A
SB_0165	SB_0165	MMGVWVFFVRV NEGGKDGGN	50	0	0	#N/A	#N/A	#N/A	#N/A	#N/A	#N/A
SB_0166	SB_0166	MDSCYPLKVEK AMLLDMGAWV SSSCELSR	11	0	0	#N/A	#N/A	#N/A	#N/A	#N/A	#N/A
SB_0167	SB_0167	MRGRKPLLSDSQ SDVLVKLYLQEE NPVMMSGLRDRR RMGDRCMNMRV FSRVNEGFMLLM WWVSEPHCVVV NM	45	2	14.61955031	0.002997002997	MGDRCMNMRVF SR	1601.704692	1802.814079	7.862381391	No
SB_0168	SB_0168	MLSPVSRRVMF DQENVVTSTESS PSRLMVGGKAR LARLARSHQKAI SGSRVWSPWERI MMRLWVRS	63	2	19.0610428	0.008484848485	IMMRLWVR	1103.60958	1263.71823	-9.212354895	No

SB_0169	SB_0169	IMRMTAPVKLQGVWMRMAVTTRAMWLIEEYAMSDFRSVCRRQMELVMIMPHRDSTRKG	408	14	24.68111573	0.000999000999	RQMELVMIMPHR	1539.78359	1683.892152	-3.836329152	Yes
SB_0170	SB_0170	IKLLAQEFDSSWAVRVSSRMFSEPRVLWV	18	0	0	#N/A	#N/A	#N/A	#N/A	#N/A	#N/A
SB_0171	SB_0171	MISSVAVNVMIKEICREISMER	12	0	0	#N/A	#N/A	#N/A	#N/A	#N/A	#N/A
SB_0172	SB_0172	MIVRGRSQVVSIRRGVVRGSEEKVGEQLNRLLLIWLKNSRGMMLMIRLWVVVLIQIMCFLESHVSGSNMIVGTISFSIGVGLGYVRSLGHMCWRLRLVGLGPPLLRRRQRLVWQ	155	1	12.06194623	0.009433962264	GRSQVVSIR	1000.577749	1144.676438	2.971509154	Yes
SB_0173	SB_0173	MVLFLLGMVGRMWGVSDMLVFLEVRW	12	0	0	#N/A	#N/A	#N/A	#N/A	#N/A	#N/A
SB_0174	SB_0174	MSRNHSFCLNYMPIRFSLILFVVTHRPDLGLGWWLMRGMT	22	0	0	#N/A	#N/A	#N/A	#N/A	#N/A	#N/A
SB_0175	SB_0175	MVKPHLQNASIRRRLRSQSDVWM	6	1	14.2310863	0.000999000999	MVKPHLQNASIRR	1704.968163	2009.156981	5.11088456	No
SB_0176	SB_0176	MLSRRCRRKWWRETRSTLRLVGG	1	0	0	#N/A	#N/A	#N/A	#N/A	#N/A	#N/A
SB_0177	SB_0177	MLLMRVMRMCLGVGLLGDLAGWCLLGASALLIGG	9	0	0	#N/A	#N/A	#N/A	#N/A	#N/A	#N/A
SB_0178	SB_0178	MGLSRIEGLFGQVVCGGLGMCFLVLHRAIIGMWLVCWLVGLVWGALWSGSEITWLGRRSLGGLRGPLLGVMGWVLLYDRHVIGGSLCVVVQVEAY	48	10	26.94818027	0.008474576271	RSLGGLR	757.4558484	901.5583248	-0.4346533091	Yes
SB_0179	SB_0179	IFSWVMRNSVRSMGVIMVGHTVVFSWGISL	11	0	0	#N/A	#N/A	#N/A	#N/A	#N/A	#N/A

SB_0180	SB_0180	ISKIFRGINSRTMGMKLWFAPQISEHWP	51	3	22.00463893	0.003636363636	IFRGINSR	961.5457216	1105.650919	-2.811209079	Yes
SB_0181	SB_0181	IASVFKPNVGTAHECKTSCDVIIMRMGASIGSTTRLSTSRSRRSPGSRNNGGSM	26	1	17.90667062	0.001002004008	RSPGSRNNGGSM	1218.552341	1362.66105	-4.858555349	No
SB_0182	SB_0182	MRRDGRAMRTRMMAGRMVQTVSISWASEMLVLVSFVVSVRKRAYRTRKQMRKMIMRAWSWKVMSSSMMGEVASCRPTCAACAIKMYRI	73	7	17.2891006	0.008849557522	TRMMAGR	821.3999924	981.4877215	9.442820089	No
SB_0183	SB_0183	MVFLMPFWKSHGGHGVGLKPALGGSIPSFFV	4	0	0	#N/A	#N/A	#N/A	#N/A	#N/A	#N/A
SB_0184	SB_0184	ILCMRVLRMCGRVGGIHMVTPGLWRVLLLLGLFASKRRLLKSWKLLMLLLLEKWMSLQMMGCFMWCMHRGSPSNVGAFRMGRESVVGRKLDLRRWMW	55	1	16.68926836	0.000999000999	ESVVGRKLDLR	1270.735698	1558.932275	4.864339307	No
SB_0185	SB_0185	MGEISEWSLLWWQMQLLLMGHSGSGLQRSTCRVVRCLVMSLLMQCQSGHLRWKERWILGLRALQQIISYCFRGVWRVS	42	0	0	#N/A	#N/A	#N/A	#N/A	#N/A	#N/A
SB_0186	SB_0186	MVLFFRSSKLQYGRLFRSLVG	13	0	0	#N/A	#N/A	#N/A	#N/A	#N/A	#N/A
SB_0187	SB_0187	MGVGMEWGLLLRRGRRRWCWGCGLLVV	20	1	20.65512067	0.001	MGVGMEWGLLLR	1360.699513	1520.792566	2.603800845	Yes
SB_0188	SB_0188	MGEVGLLWLGRIRRRGAFGIGLWQGVLYW	5	0	0	#N/A	#N/A	#N/A	#N/A	#N/A	#N/A

SB_0189	SB_0189	MRAGVGEREVRVRSLCCLCGETPYRGHRLLGELVSCQSLRLWWVLLWRRLLQMHGLWR	43	2	25.21398491	0.006564551422	AGVGEREVR	971.5148174	1115.609647	6.50557754	No
SB_0190	SB_0190	IFMLNCKFEEAASNLPGLLPPFFPAAGEVDWSQLIRVLSC	2	1	12.7887681	0.006651884701	IFMLNCK	867.434644	1212.650713	7.869148105	No
SB_0191	SB_0191	ICVQLMQSGVLQSLAVTEIKYCNLLRALKALGLYLT	8	0	0	#N/A	#N/A	#N/A	#N/A	#N/A	#N/A
SB_0192	SB_0192	IWAKSRLAGAGLLGRGGWMELRVLVMLACFRCEMVVGSWCWSLSWVVGMR	24	3	30.49572606	0.001004016064	SRLAGAGLLGR	1069.635595	1213.734458	2.661272284	Yes
SB_0193	SB_0193	MLRFCVAGFGLIHLNCLLWWMRLREWGEGLRLVRERFGMWLRWGLVFVMWEEAGRMSEGCLG	65	1	19.80232168	0.000999000999	LVRERFGMWLR	1461.802668	1605.90347	0.8099306964	Yes
SB_0194	SB_0194	MRLLAWGNTWWQLLWNEGLFFWLELE	0	0	0	#N/A	#N/A	#N/A	#N/A	#N/A	#N/A
SB_0195	SB_0195	MGPMAYLADLTLGWGVMGGTENFGFSGMGSILMVLEMRGFKLLLFTLSK	5	0	0	#N/A	#N/A	#N/A	#N/A	#N/A	#N/A
SB_0196	SB_0196	MWDCLGYCSQCADQGVVWVWCSPWSEDWVNG	0	0	0	#N/A	#N/A	#N/A	#N/A	#N/A	#N/A
SB_0197	SB_0197	MMSFTGEGALWSRPYFSCPFVQGGIWX	1	0	0	#N/A	#N/A	#N/A	#N/A	#N/A	#N/A
SB_0198	SB_0198	MSWSNIEVVNPIVDMDSRMGLRCYP	15	0	0	#N/A	#N/A	#N/A	#N/A	#N/A	#N/A
SB_0199	SB_0199	ISLVKSKRQLNPRGAIHTGPYLRNKWLCYLCTVRVPRPLNMCHWAGGASNTGDARGDVFGKQAG	28	0	0	#N/A	#N/A	#N/A	#N/A	#N/A	#N/A

SB_0200	SB_0200	MLCLVMIFHLSLAVLYLLRQVSISIAYTLFG	1	0	0	#N/A	#N/A	#N/A	#N/A	#N/A	#N/A
SB_0201	SB_0201	MLEEGDGRCVRASGPCSTKHSTLSLLLNPPSTLKFHKGYRSFLG	28	3	12.21386031	0.004243281471	MLEEGDGR	905.3912624	1065.481706	6.149467885	No
SB_0202	SB_0202	MGWARGGEVDRGLSITEQAPLEGYEAPPGPLSFKLWLVVFWRAVLLI	13	1	17.17685495	0.000999000999	MGWARGGEVDR	1232.572012	1376.671919	1.585656856	Yes
SB_0203	SB_0203	MNCGGCLWGLVGSGYGVSSGVCVLGRMGGGCIDEISSMGVGGENNVLVGGWLLKVHTAKR	7	0	0	#N/A	#N/A	#N/A	#N/A	#N/A	#N/A
SB_0204	SB_0204	MCLSAVARSGGGGVWWKFFVMMSVWKVAVQTFNCYYYVLQALIN	18	0	0	#N/A	#N/A	#N/A	#N/A	#N/A	#N/A
SB_0205	SB_0205	MGCSGSSVSQCYRVHTPQTKMPNAWRAPVSG	21	6	11.77834789	0.001998001998	MGCSGSSVSQCYR	1363.531865	1621.660993	9.797445322	No
SB_0206	SB_0206	MSYLRGTCGLFRLYDPEVGTRCRMQFTLATPKCYGPGARRVALLCGMLISRRMVVKGPLSEGGHPWGREGIWL	119	6	33.32383618	0.000999000999	VALLCGMLISR	1174.656587	1391.768589	4.651040481	No
SB_0207	SB_0207	MCDSWGLIAVLACKHGEGVLMWIGFLCTTGGQVFMVPYNIHGGWQ	13	0	0	#N/A	#N/A	#N/A	#N/A	#N/A	#N/A
SB_0208	SB_0208	MAVVDGWVNTWVVPKSASPWKNRE	11	0	0	#N/A	#N/A	#N/A	#N/A	#N/A	#N/A
SB_0209	SB_0209	MVELKTFSLICPWKKVFISGLQDWCISLYYKDRPIWVFCFQLGR	30	0	0	#N/A	#N/A	#N/A	#N/A	#N/A	#N/A
SB_0210	SB_0210	IGLVGEMLCFVVWMYGGWGLLLGWGWMVMGQGRLLVC	0	0	0	#N/A	#N/A	#N/A	#N/A	#N/A	#N/A

SB_0211	SB_0211	MGNIIRAWCGEG CLRGWLGYNCL GRLGGLVRMVL MSLRREGREVSR GRLWLCSKGGR WFYRNGRWFLG GCLIPFRARMGG GVLLGLQ	182	5	19.94206225	0.000999000999	MVLMSLRREGR	1346.727458	1506.831371	-4.578775128	No
SB_0212	SB_0212	MLRRHWREGSG WFSHNLRLEWC GRLMKRRLRRL VSSAWLGMVLW WFGGSGRRQGV SRSFIMRRCWMG WGGRWMSG	89	2	12.08128351	0.008083140878	RCWMGWGGR	1107.485449	1308.600806	6.261558495	No
SB_0213	SB_0213	MLQRWLLRSILR HGGQGLRSWWV F	2	0	0	#N/A	#N/A	#N/A	#N/A	#N/A	#N/A
SB_0214	SB_0214	MKERGQGWFGR ILLVRGLCMIMG V D	16	0	0	#N/A	#N/A	#N/A	#N/A	#N/A	#N/A
SB_0215	SB_0215	IMPFWVEVMME VEIWCCEIVLGNS FSSQVRSRRSRGR FWLVRRPR	12	0	0	#N/A	#N/A	#N/A	#N/A	#N/A	#N/A
SB_0216	SB_0216	IVVWKGDAGEM LLVMRNPANRLP AARRLMGFSRVG LFSLMLVRVGKR GWPVRVRRIIRV L	77	1	18.13639941	0.000999000999	GDAGEMLLVMR	1190.578736	1334.687841	-5.255125499	No
SB_0217	SB_0217	MRVMDRAQAFV YDMFAVSMMWS LE	23	0	0	#N/A	#N/A	#N/A	#N/A	#N/A	#N/A

SB_0218	SB_0218	IPANARLPMVRE VEVRGMVLSSPPI FRMSCSLLRLWM MDPEHMNSMAL KKAWVQMCRNA RCGWLMPIVTIM SPSWLEVEKATIF LMSFCVRAQTAA NRVVMAPKHSV RVWISGLFSARG WKRMSKKIPATT MVLEWSRAETG VGPSMAEGSQG WRPNWADLPAA ARRRPSSGVRLG LAFRRAICCGSHE LECRMNHAKAR MKPMSPMRLYRI AWMAAVLASAR AYHQLMSKKDMI PTPSQPMNSWNR LLAVTKISMVIRK MSRYLKNWLMF GSEFMYHSENSM MDHVTNNATGM NIMEK	254	11	41.69602147	0.000999000999	EVEVRGMVLSSPP IFR	1814.971241	1975.083261	-7.59190579	No
SB_0219	SB_0219	MMTSWSRHMNI VVGKRLMMKVD ATMDFT	31	5	16.35315213	0.001210653753	HMNIVVGK	896.4901828	1200.683129	5.095019283	No
SB_0220	SB_0220	MGGIREVRVRVV MVVCMVITFIWS CTKIFGA	10	0	0	#N/A	#N/A	#N/A	#N/A	#N/A	#N/A
SB_0221	SB_0221	MGGEWGMGVW TWGCFLVWMRV LCC	13	0	0	#N/A	#N/A	#N/A	#N/A	#N/A	#N/A
SB_0222	SB_0222	MGMVQGRGRLC VLSGGWEWVW GVLYHSRLVLRV LRQVLLTQRWG LRHGL	15	3	17.1813702	0.006928406467	MGMVQGRGR	990.485115	1150.574672	6.469627156	No
SB_0223	SB_0223	MLSCWLRSLMVL GQWEWVVECLV SLGCCECKLVRW VGEGSLLGCRMG SMCLRSGVLAGC LIGWW	52	0	0	#N/A	#N/A	#N/A	#N/A	#N/A	#N/A
SB_0224	SB_0224	MVVHWMSGVGL PWLWGVGVR	9	0	0	#N/A	#N/A	#N/A	#N/A	#N/A	#N/A

SB_0225	SB_0225	MPVSGGGFEAKVMFGCKVKY	35	1	17.54348204	0.000999000999	MPVSGGGFEAK	1078.511705	1382.713954	-2.302300023	Yes
SB_0226	SB_0226	IKATAISRMVSRIRIVKMMSVEGRLMVDIARVALPIRCMSRWPAVMLAVRRTARAIGWMSRLMVSMMTSMGMRGVGVPCGKKWARAFLILERKPMITVPAHKGMAMARFMDSWVVGVNEWGRSPRRLVVAMKMIKDTSMRDQVRPLVLCMVIICFEVSLISHCWVVISRLLMRYLEVGINRGGNRMISTAAGRPRIVGAMNEANRFSFILVLRVCYNFLFLWALVREVGGSLCLMFLVGWWGMV	515	22	34.85655731	0.000999000999	AIGWMSRLMVSMMTSMGMRGVGVPCGK	2872.353417	3233.551855	6.845155335	No
SB_0227	SB_0227	MRALFQRFLGELILGRWAWNCGLLHRFQSIDRSMPPVV	12	0	0	#N/A	#N/A	#N/A	#N/A	#N/A	#N/A
SB_0228	SB_0228	MMGEVCRSWRLVRRSRCTRRFSTIGGQLIWW	11	0	0	#N/A	#N/A	#N/A	#N/A	#N/A	#N/A
SB_0229	SB_0229	MEAMGLAWNQLWGVRFLPFLSRFYVYGFFECVVGWGASM	8	0	0	#N/A	#N/A	#N/A	#N/A	#N/A	#N/A
SB_0230	SB_0230	IASVECGESAKYFDAGGDSDDYGSGGEMCSCVYVYSYCKYMVCSHDKP	8	0	0	#N/A	#N/A	#N/A	#N/A	#N/A	#N/A
SB_0231	SB_0231	IANSKKQLQTCRGFSRLFSRRREK	36	12	21.06855371	0.00218579235	GFSRLFSRR	1124.62028	1268.717786	3.615045629	Yes
SB_0232	SB_0232	IGSIGYGSLSGEYIVEEDSY	1	0	0	#N/A	#N/A	#N/A	#N/A	#N/A	#N/A
SB_0233	SB_0233	MLEIVMGMETYHMSNARVSGRKFFHRRCMSWS	53	0	0	#N/A	#N/A	#N/A	#N/A	#N/A	#N/A

SB_0234	SB_0234	IVVVRVFIMMMFVYSAMKNRAKGPAAYSMLKPETSSDSPSARSKGVRLVSASVEMNHIMAKGHDGRSNQRCSCVVMRVERLKEPLISNVDSRMMARVTSYEIVWATARSAPI RA	165	4	21.622387	0.000999000999	LVSASVEMNHIMAK	1528.774131	1816.968939	5.149099863	No
SB_0235	SB_0235	MNRRPRLRLTRGLGMGRGVHSRRAMVRAKVGAVM	31	2	14.75986827	0.005020080321	GVHSRRAMVR	1167.640697	1311.744847	-1.567282974	Yes
SB_0236	SB_0236	MVDVAGFRGSLVKSFMASAKGCSSP	17	1	16.69596443	0.008484848485	MVDVAGFR	893.4429008	1053.531901	7.592758242	No
SB_0237	SB_0237	IFRSVSIRNAIAIRMGTMRSRRLAMGMLLRRGIEPLTVKF	65	3	20.62049424	0.005773672055	RLAMGMLLR	1059.604495	1235.707948	-9.330817665	No
SB_0238	SB_0238	INKLKLHRVFSSCCVMPASSRAGQFHWLKVRDSWTLVEPFMQVPI	47	3	15.62436483	0.00218579235	AGQFHWLK	985.5133602	1273.722598	-3.994077948	Yes
SB_0239	SB_0239	MLVMLEVMFLVNRRGKICRVPFTFFNLSLWACLCWVDSEGNNDLLVDCRYWAVNCQFSVLIWRRLMRRRMFSCYLY	47	0	0	#N/A	#N/A	#N/A	#N/A	#N/A	#N/A
SB_0240	SB_0240	MDWSNWVWGVQLYVWDFLGSGCWAWTLS	0	0	0	#N/A	#N/A	#N/A	#N/A	#N/A	#N/A
SB_0241	SB_0241	MGVKFFTLSTRFFPSVQRAVPLWTNS	4	1	13.41600805	0.008403361345	FFTLSTR	870.4599302	1014.555688	6.236398252	No
SB_0242	SB_0242	MSFEVYLRRVTGGVYALQGPVQLSTLLLVYC	5	0	0	#N/A	#N/A	#N/A	#N/A	#N/A	#N/A
SB_0243	SB_0243	MRAIVVFWGRKCSPFLATSWATPWPNVFTWVLALTL	2	0	0	#N/A	#N/A	#N/A	#N/A	#N/A	#N/A
SB_0244	SB_0244	MVGYLIPVWVLAIVCSDMLKPLS	0	0	0	#N/A	#N/A	#N/A	#N/A	#N/A	#N/A
SB_0245	SB_0245	ILCQLEFFTTQVSFSFIGEGVI	3	0	0	#N/A	#N/A	#N/A	#N/A	#N/A	#N/A

SB_0246	SB_0246	IAACLMLVPFDR GDLEGELTGTGM LACVILLRANRK ARTKPICLWGDV SPSKHFQCIALRR	32	0	0	#N/A	#N/A	#N/A	#N/A	#N/A	#N/A
SB_0247	SB_0247	MGLAAVCVCW VGWAGVVLMR LVVWEWEGKM MC	21	0	0	#N/A	#N/A	#N/A	#N/A	#N/A	#N/A
SB_0248	SB_0248	MPPKDKIWNLVR LVLGFFVFGVWQ RCV	22	0	0	#N/A	#N/A	#N/A	#N/A	#N/A	#N/A
SB_0249	SB_0249	MLNVGAMNNR MRQESKTDTAT	22	1	17.63291836	0.002030456853	MLNVGAMNNR	1118.532457	1262.64688	-9.768674111	No

Filtered list based on validation (only "Yes")

Prot_Accession	Description	Prot_Seq	PepQuery_hits	Confident_hits	Best_Score	pvalue	Peptide_Sequence	Peptide_Check_Mass	Exp_Pep_Mass	Error_mass_ppm	Validation	mt gene or region	LEN(prot)
SB_0086	SB_0086	MFADRWLFSTNHKDIGTLYLLFGAWAGVLGTALSLLIRAELGQPGNLLGNDHIYNVIVTAHAFVMIFFMVMPIMIGGFGNWLVPLMIGAPDMAFPRMNNMSFWLLPPSLLLLLASAMVEAGAGTGWTVYPPLAGNYSHPGASVDLTIFSLHLAGVSSILGAINFITTIINMKPPAMTQYQTPLFVWSVLITAVLLLLSLPVLAAGITMLLTDRNLNTTFFDPAGGGDPILYQHLFWFFGHPEVYILILPGFGMISHIVTYYSGKKEPFGYMGMVWAMMSIGFLGFIVWAHHMFTVGMDVDTRAYFTSATMIIAIPTGVKVFSWLATLHGSNMKWSAAVLWALGFIFLFTVGGLTGIVLANSSLDIVLHDTYYVVAHFHYVLSMGAVFAIMGGFIHWFPLFSGYTLDQTYAKIHFTIMFIGVNLTFFPQHFLGLSGMPRRYSDYPDAYTTWNILSSVGSFISLTAVMLMIFMIWEAFASKRKVLMVEEPSMNLEWLYGCPPPYHTFEEPVYMKSRQKRKESNPPKLVSSQPHGLHDFFKKVLEKPFHNFVKVKL	151	91	90.04728257	0.000999000999	VLMVEEPSMNLEWLYGCPPPYHTFEEPVYMK	3727.711022	4088.940668	-2.215073813	Yes	RefProt COX1	553
SB_0063	SB_0063	MLKLIVPTIMLLPLTWLSKKHMIWINTTTHSLIISIIPLLFFNQINNNLFSCSPTFSSDPLTTPLLMLTTWLLPLTIMASQRHLSSEPLSRKKLYLSMLISLQISLIMTFTATELIMFYIFFETTLIPTLAIITRWGNQPERLNAGTYFLFYTLVGSLPLLIALIYTHNTLGSLNILLLTLTAQELSNSWANNLMWLAYTMAFMVKMPLYGLHLWLPKAHVEAPIAGSMVLAAVLLKLGGYGMMRLTLILNPLTKHMAYPFLVLSLWGMIMTSSICLRQTDLKSLIAYSSISHMALVVTAILIQTPWSFTGAVILMIAHGLTSSLLFCLANSNYERTHSRIMILSQGLQTLLPLMAFWWLLASLANLALPPTINLLGELSVLVTTFSWSNITLLLTGLNMLVTALYSLYMFTTTQWGSLTHHINNMKPSFTRENTLMFMHLSPILLLSLNPDIITGFSSCKYSLTKTSDCESDNRGLRPLIYRESSQELLTHAPMSNNMAFSTFKG	87	8	84.24539063	0.000999000999	AHVEAPIAGSMVLAAVLLK	1889.080786	2177.288583	-1.661575297	Yes	RefProt ND4	506
SB_0056	SB_0056	MAHAAQVGLQDATSPIMEELITFHDHALMIIFLICFLVLYALFLTLTTKLTNTNISDAQEMETVWTILPAIILVLIALPSLRILYMTDEVNDPSLTIKSIGHQWYWTYEYTDYGGLIFNSYMLPPLFLEPGDLRLLDVDNRVVLPIEAPIRMMITSQDVLHSWAVPTLGLKTDAIPGRLNQTTFTATRPGVYYGQCSEICGANHSFMPIVL	779	656	76.27685888	0.000999000999	MMITSQDVLHSWAVPTLGLK	2226.154024	2514.353794	1.755039781	Yes	RefProt COX2	227

		ELIPLKIFEMGPVFTL											

SB_0024	SB_0024	ILVQLQMKVMTMHTTMTTLTLT SLIPPILTTLVNPNKKNSYPHYVK SIVASTFIISLFPTTMFMCLDQEVII SNWHWATTQTTQLSLSFKLDY FSMMFIPVALFVTWSIMEFSLWY MNSDPNINQFFKYLLIFLITMLILV TANNLFQLFIGWEGVGIMSFLLIS WWYARADANTAAIQAILYNRI GDIGFILALAWFILHSNSWDPQQ MALLNANPSLTPLLGLLLAAAGK SAQLGLHPWLPSAMEGPTPVSAL LHSSTMVVAGIFLLIRFHPLAENS PLIQTLTLCLGAITTLFAAVCALT QNDIKKIVAFSTSSQLGLMMVTIG INQPHLAFLHICTHAFFKAMLFM CSGSIIHNLNNEQDIRKMGGLLKT MPLTSTSLTIGSLALAGMPFLTGF YSKDHIIETANMSYTNAWALSITL IATSLTSAYSTRMILLTLTGQPRFP TLTNINENNPTLLNPIKRLAAGSL FAGFLITNNISPASPFQTTIPLYLK LTALAVTFLGLLTALDLNYLTNK LKMKSPLCTFYFSNMLGFYPSITH RTIPYLGLLTSQNLPLLLLDLTWL EKLLPKTISQHQISTSIITSTQKGMI KLYFLSFFFPLILTLLLIT	113	63	76.17222727	0.00099900099 9	FPTLTNI NE NNPTLL NPIKR	2308.253868	2596.46168	-1.396327951	Yes	RefProt ND5	612
SB_0057	SB_0057	MPQLNTTVWPTMITPMLLTLFLIT QLKMLNTNYHLPPSPKPMKMKN YNKPWEPKWTKICSLHSLPPQS	328	277	64.10598268	0.00099900099 9	MLNTNY HL PPSPKP MK	1866.948398	2299.260345	-2.484358335	Yes	RefProt ATP8	68
SB_0089	SB_0089	MMTHQSHAYHMVKPSPWPLTGA LSALLMTSGLAMWFHFHSMTLL MLGLLTNTLTMYQWWRDVTRES TYQGHHTPPVQKGLRYGMILFIT SEVFFFAGFFWAFYHSSLAPTPQL GGHWPPTGITPLNPLEVPLLNTSV LLASGVSITWAHHSLMENNRNQ MIQALLITILLGLYFTLLQASEYFE SPFTISDGIYGSTFFVATGFHGLH VIIGSTFLTICFIRQLMFHFTSKHH FGFEAAAWYWHFVDVVWLFLY VSIYWWGSYSFSMNSTVNFQLTS FDNIQKRVMNFALILMINTLLALL LMIITFWLPQLNGYMEKSTPYEC GFDPMSPARVPFSMKFFLVAITFL LFDLEIALLLPLPWALQTTNLPLM VMSSLLLIIILALSLAYEWLQKGL DWTELVYSLNKTNDFDSLNYDN HIYQMPLIYMNIMLAFTISLLGML VYRSHLMSSLLCLEGMMLSLFIM ATLMTLNTHSLLANIVPIAMLV FAACEAAVGLALLVSISNTYGLD YVHNLNLLQC	111	69	55.11127489	0.00099900099 9	ESTYQG HH TPPVQ K	1607.769182	1895.97657	-1.701906999	Yes	RefProt COX3	520

SB_0017	SB_0017	MNENLFASFIAPTILGLPAAVLIIL FPPLLIPTSKYLINNRLITTQQWLI KLTSKQMMTMHNTKGRTWSLM LVSLIIFIATTNLLGLLPHSFTPTT QLSMNLAMAIPLWAGTVIMGFRS KIKNALAHFLPQGTPTPLIPMLVII ETISLLIQPMALAVRLTANITAGH LLMHLIGSATLAMSTINLPSTL IIFTILILLTILEIAVALIQAYVFTLL VSLYLHDNT	130	89	51.46183017	0.001020408163	LITTQQ WLI K	1242.733572	1530.93992	-1.428598097	Yes	RefProt ATP6	226
SB_0005	SB_0005	MPPSSANPDEGYKVSASTHVKTL GQGVAHEVARNGLHFLPQKTT MALMKLKGRRWI	111	8	31.85069597	0.001998001998	TLGQGV AH EVAR	1236.657452	1380.764859	-3.848103668	Yes	12S sense (overlaps MOTS-c)	56

SB_0085	SB_0085	INPLAQPVIYSTIFAGTLITALSSHWFFTWVGLEMNMLAFIPVLTKKMNPRSTEAAIKYFLTQATASMILLMAILFNNMLSGQWTMTNTTNQYSSLMIMMAMAMKLGMAPFHFWVPEVTQGTPLTSGLLLLTWQKLAPISIMYQISPSLNVSLLLTLSILSIMAGSWGGLNQTQLRKILAYSSITHMGWMMAVLPYNPNMTILNLTIYIILTTTAFLLLNLNSSTTTLLLSRTWNKLTWLTPLIPSTLLSLGGLPPLTGFLPKWAIIEEFTKNNSLIIPTIMATITLLNLYFYLRLIYSTSITLLPMSNNVKMKWQFEHTKPTPFLPTLIALTTLLLPISPFMLMIL	68	17	30.98510606	0.001048218029	WAIIEEFTK	1135.591332	1423.796681	-0.8391981488	Yes	RefProt ND2	347
SB_0192	SB_0192	IWAKSRLAGAGLLGRGGWMELRVLVMLACFRCEMVVGSWCWSLSWVVGMR	24	3	30.49572606	0.001004016064	SRLAGAGLLGR	1069.635595	1213.734458	2.661272284	Yes	ND2, antisense	50
SB_0110	SB_0110	MYYSDGYWGVSWGMGVRGWGLGECFSGVSDGGRIGAVGERVWWGGGCGKL	54	4	28.02515	0.000999000999	IGAVGERVWWGGGCGK	1630.803791	1976.033302	-1.964915008	Yes	ND6 sense (ND6 is on other strand), frameShift	50
SB_0007	SB_0007	MVGRFMGRGDKPTEPGDSWLSKMES	54	12	27.52984605	0.001998001998	MVGRFMGRGDKPTEPGDSWLSK	2450.183426	2914.478541	0.3292530069	Yes	16S sense	25
SB_0178	SB_0178	**MGLSRIEGLFGQVVCGGLGMCFLVLHRAIIGMWLVCWLVGLVWGALWSGSEITWLGRRSLGGLRGPLLGVMGWVLLYDRHVIGGSLCVVVQVEAY**	48	10	26.94818027	0.008474576271	RSLGGLR	757.4558484	901.5583248	-0.4346533091	Yes	COX3, antisense	95
SB_0055	SB_0055	MICCSALSPRIHLSFHRRWPDWHCISKLITRHRTTRHVLRCSPLPLCPINRSCICHHRRLHSLISPILRLHPRPNLRQNPFHYHIHRRKSNFLPTTLSRPIRNAPTLLGLPRCMHHMKHPIICRLIHFSNSSNINNFHDLRSLRFEAKSPNSRRTLHKPGVTMWMPPTLPHIRRTRMHKI	99	8	26.39510071	0.000999000999	TTRHVLRCSPLPLCPINR	2075.124397	2333.278658	-3.949405601	Yes	COX1 sense, frameShift	180
SB_0125	SB_0125	IMCCRAGRGLLEVWKRRLGLRRQRFLG	15	1	25.62950155	0.000999000999	IMCCRAGRGLLEVWK	1733.889112	2152.138023	-3.205659403	Yes	ATP6, antisense	27
SB_0132	SB_0132	**MWSLPRRLPGWPSSARMRRLRAVPRTPAHAPNNRYSVPMSLWFVENSQRSANISGGEVKWLSEALDCKSKDRG**	107	5	24.68961083	0.002040816327	WLSEALDCK	1063.500807	1408.724693	1.226049523	Yes	Non coding (antisense between nd2 and cox1)	73
SB_0169	SB_0169	**IMRMTAPVKLQGVWMRMAVTTRAMWLIEEYAMSDFRSVCRRQMELVMIMPHRDSTRKG**	408	14	24.68111573	0.000999000999	RQMELVMIMPHR	1539.78359	1683.892152	-3.836329152	Yes	ND4, antisense	58
SB_0058	SB_0058	MTPNRGPLSPPNDLRPSHVISLPLHNAPHTRPTNQHTNHMPMMARCNTRKHMPRPPHTTCPKRPSMRDNPIYYLRSFFLRRIFLSLLPLQPSPYPPIRRALAPNRHHPAKSPRSPTP	35	2	24.59374409	0.000999000999	RPSMRDNPIYYLR	1679.856552	1968.066962	-3.172199407	Yes	COX3 sense, frameShift	134

		KHIRITRIRSINHLSSP											
SB_0141	SB_0141	IRLWRMGRRGLRRIRCWSLRLVR TPLRQGRRGFGWSLLVWR	34	5	24.18824918	0.008869179601	TPLRQGR	826.4773112	970.5764446	3.041597953	Yes	ND1, antisense	41
SB_0008	SB_0008	ILLRMSLRQIKTLNWQLTAQYLQ STNKSLLPSLSTQHRHAHKERLK KVKGTRQILPRLFTKNITSSITSIR GTACPVTHV	43	1	23.99545008	0.000999000999	LFTKNITSSI TSIR	1579.893313	1868.103082	-2.998073063	Yes	16S sense, complementary to SHLP1 and SHLP4	80
SB_0136	SB_0136	MVAMMVGMMRLLFFVNSSMMA HLGKKPVSGGRPPRERRVDGIKG VSHVSLFQVRDSSRVVVLEFKLSS RNAVVVRMM	90	2	22.19791862	0.002688172043	MVAMMV GMMR	1155.509478	1347.595171	0.8468832668	Yes	ND2, antisense	77

SB_0180	SB_0180	ISKIFRGINSRTMGMKLWFAPQIS EHWP	51	3	22.00463893	0.00363636363 6	IFRGINS R	961.5457216	1105.650919	-2.811209079	Yes	COX2, antisense	28
SB_0003	SB_0003	IIFPSHSHTTNLINTTPAHPTQHTH TAANPMPRTNQTPKTPPTVYVA YLLKAMHWKCLDGLTSPHKQM GLVLAFLLALSKITHASIPVPVSS PSKSPRSKGTSIKHAAMQLKTLS LATPPRETAVINL	52	3	21.5925048	0.00874316939 9	TLSLATP PR	954.5498034	1098.655792	-3.548999013	Yes	12S sense	128
SB_0067	SB_0067	MGEGLEENPTNPITKPTLNRNKA YIIILARTTTTTNDMKNHRCISTTR TPMTPMRKTNPLMKLINHSFIDLP TPSNISAWWNFGSLLGACLILQIT TGLFLAMHYSPDASTAFSSIAHIT RDVNYGWIIRYLHANGASMFFIC LFLHIGRGLYYGSFLYSETWNIGII LLLATMATAFMGYVLPWGQMSF WGATVITNLLSAIPYIGTDLVQWI WGGYSVDSPTLTRFFTFHFILPFII AALATLHLLFLHETGSNNPLGITS HSDKITFHPYYTIKDALGLLLFLL SLMTLTLFSPDLLGDPDNYTLANP LNTPPHIKPEWYFLFAYTILRSVP NKLGGVLALLLSILILAMIPILHMS KQQSMMFRPLSQSLYWLLAADL LILTWIGGQPVSYPFTIIGQVASVL YFTTILILMPTISLIENKMLKWACP CSMN	63	7	21.38188719	0.00221238938 1	KTNPLM K	830.4683856	1262.772811	1.41407854	Yes	RefProt CYTB	436
SB_0231	SB_0231	IANSKKQLQTCRGFSRLFSRRREK	36	12	21.06855371	0.00218579235	GFSRLFS RR	1124.62028	1268.717786	3.615045629	Yes	Non coding (antisense between nd2 and cox1)	24
SB_0187	SB_0187	MGVGMEWGLLLRRGRRRWCWG CGLLVV	20	1	20.65512067	0.001	MGVG ME WGLLL R	1360.699513	1520.792566	2.603800845	Yes	COX1, antisense - frameshift of Gau	27
SB_0050	SB_0050	IPTTQLKLQHHDPTTISHLKQANM TNTLNSIHPPLPRRPAPANRLFAQ MGHYRRIHKKQ	41	1	20.32219288	0.00099900099 9	QANMTN T LNSIHPP LPR	1902.973367	2047.067215	4.036097747	Yes	ND2 sense, frameShif t	59
SB_0193	SB_0193	MLRFCVAGFGLIHLNCLLWWMR LREWGEGLRLVRERFGMWLRWG LVFVMWEEAGRMSEGCLG	65	1	19.80232168	0.00099900099 9	LVRERF GM WLR	1461.802668	1605.90347	0.809930696 4	Yes	ND2, antisense	62
SB_0119	SB_0119	MMINKRDDMTISGRLVVCRAHG RGKRRAISRSNNKKVMATKKNF MEKGTRAGDMGSKPHS	64	4	19.56720182	0.00242424242 4	DDMTIS GR	893.3912624	1053.484827	3.257502508	Yes	ND3, antisense	60
SB_0015	SB_0015	MVLNLRVHRLRRTNLQLLHTSPI IPRTRRPATPWRWQSSSTPDWSP HSYNNYITRRLALMSCPHIRLKN RCNSRTSKPNHFHRYTTGGML RSMLWNLWSKPQFHAHRPRINS PKNLWNRARIYPMAPPLPPLEPT VKLT	49	2	19.26859725	0.00884955752 2	NLWNR AR	928.4991084	1072.603732	-2.365401482	Yes	COX2 sense, frameShif t	139
SB_0022	SB_0022	**MRHNYNKLHLPTTNRPKIAHC MLFNQPHSPRSNSHSHPNPLKL HRRSHSHNRPRAYILITILPSKL KLRTHSQSHHNPLSRTSNSTPT**	62	11	19.17011656	0.00201816347 1	LHLPTTN RPK	1175.677457	1463.877578	2.758316996	Yes	ND4 sense, frameShif	99

		NS<br>FLMTSSKPR										t	
SB_0138	SB_0138	MGCDRWHGEFWILRDGFDSHSP<br>RNKGV	20	5	18.53962327	0.00314465408<br>8	DGFDSH<br>SPR	1016.431152	1160.534518	-1.106494702	Yes	Non coding<br>(sense<br>between<br>ND1 and<br>ND2)	27
SB_0001	SB_0001	IWYFRLGGMHAMALRDAGAGAP<br>YVAVSVFDSCLILLFIAPTFNITGE<br>HTY	37	4	18.28136369	0.00102040816<br>3	LGGMHA<br>M<br>ALR	1055.536813	1215.632296	1.251744129	Yes	D-loop, sense	50
SB_0225	SB_0225	MPVSGGGFEAKVMFGCKVKY	35	1	17.54348204	0.00099900099<br>9	MPVSGG<br>GF<br>EAK	1078.511705	1382.713954	-2.302300023	Yes	COX3,<br>antisense	20

SB_0053	SB_0053	MKITSELVKRGLTPVFRFTVQCFT<br>QPFYLTPTDVRRPLTILYKPQRHW<br>NTMPIIRRMSWSPRHSSKPPYSSR<br>AGPARQPSR	31	2	17.19087616	0.00100100100<br>1	HWNTM<br>PIIRR	1322.702959	1466.810724	-3.863706822	Yes	COX1<br>sense,<br>frameShif<br>t	81
SB_0202	SB_0202	MGWARGGEVDRGLSITEQAPLEG<br>YEAPPGPLSFKLWLVVFWRAVLLI	13	1	17.17685495	0.00099900099<br>9	MGWAR<br>G<br>GEVDR	1232.572012	1376.671919	1.585656856	Yes	12S,<br>antisense	47
SB_0068	SB_0068	ILCSFMGKQIWVPPKYWLTHQQP<br>LCISYITASHHEYCTVP	13	4	16.55336317	0.00109289617<br>5	ILCSFMG<br>K	897.4452082	1242.673333	-2.022001366	Yes	D-loop,<br>sense	40
SB_0163	SB_0163	MGDCAVCDARVESEYVGEMKCA	15	1	16.17840283	0.00507614213<br>2	VESEYV<br>GE<br>MK	1169.527414	1617.826131	1.490421419	Yes	ND5,<br>antisense	22
SB_0238	SB_0238	INKLKLHRVFSSCCVMPASSRAGQ<br>FHWLKV RDSWTLVEPFMQVPI	47	3	15.62436483	0.00218579235	AGQFH<br>WLK	985.5133602	1273.722598	-3.994077948	Yes	16S<br>antisense<br>(overlaps<br>humanin)	45
SB_0235	SB_0235	MNRRPRLRLTRGLGMGRGVHSR<br>RAMVRAKVGAVM	31	2	14.75986827	0.00502008032<br>1	GVHSRR<br>A<br>MVR	1167.640697	1311.744847	-1.567282974	Yes	ND1,<br>antisense	34
SB_0046	SB_0046	MAEPGNRMKLKTLQSEVQFLFLT<br>TYPWPTS YSSLYPF	18	3	13.93237678	0.00886917960<br>1	MAEPGN<br>R	773.3490056	917.4533302	-2.447302268	Yes	Non<br>coding<br>(antisense<br>between<br>16S and<br>ND1)	37
SB_0032	SB_0032	MHQSCKPEMKTFFQGQIREKVFN<br>STISTQS	20	2	13.83337106	0.00300300300<br>3	MHQSCK<br>PEMK	1217.535493	1722.853313	2.770027249	Yes	Non<br>coding<br>(sense<br>between<br>CYTB and<br>d-loop)	30
SB_0029	SB_0029	IHRPPHPIQHLRMMKLRLTPWRL<br>PDPPNHHRTIPSHALLTRRLNRLFI<br>NRPHHSRRKLWLNHPLPSRQWR<br>LNILYLPLPTHRARPMLRIISLLRN<br>LKHRHYPPACNYSNSLHRLCPPV<br>RPNIILRGHSNYKLTIRHPMHWD<br>RPSSMNLRRLLSRQSHPHTILYLS<br>LHLALHYCSPSNTPPPILARNGIK<br>QPPRNHLPFR	121	2	13.82029738	0.00442477876<br>1	LRLTPW<br>R	940.5606422	1084.660857	1.728571636	Yes	RefProt<br>CYTB	199
SB_0172	SB_0172	MIVRGRSQVVSIRRGVVRGSEEK<br>VGEQLNRLLLIWLKNSRGMMLM<br>IRLWVVVVLIQIMCFLESHVSGSN<br>MIVGTISFSIGVGLGYVRSLGHM<br>CWRLRLVGLGPPLLRRRQRLVWQ	155	1	12.06194623	0.00943396226<br>4	GRSQVV<br>SIR	1000.577749	1144.676438	2.971509154	Yes	ND4,<br>antisense	114
SB_0049	SB_0049	IFYLSRPRNKHASFYSSSNQKNK<br>PSFHRSCHQVFPHASNRIHNPSNS<br>YPLQQYTLRTMNHNQYYQSMLII<br>NNHNSYSNKTRNSPLSLLSPR<br>GYPRHPSDIRPASSHMTKTSPHLN<br>HMPNLSLTKRKPSPHSLNLIHHSR<br>QLRWIKPNPATQNLSMLLNYPHR<br>MNNSSSTVQP	122	4	12.05265959	0.00765864332<br>6	IFYLSRP<br>R	1050.59742	1338.79671	3.632896529	Yes		172
SB_0103	SB_0103	MLTSKYSNQPSTITHQLQLQSHPS<br>PTRMPTNLPTLNST	125	3	11.44115982	0.00299700299<br>7	MPTNLPT<br>L<br>NST	1187.585595	1347.676777	4.321420165	Yes	D-loop,<br>sense	38

ANTIBODY PRODUCTION (antigen in red)

MGLSRIEGLFGQVVCGGLGMCFLVLHRAIIGMWLVCWLVGLVWGALWSGSEITWLGRRSLGGLRGPLLGVMGWVLLYDRHVIGGSLCVVVQVEAY

MWSLPRRLPGWPSSARMRRLRAVPRTPAHAPNNRYSVPMSLWFVENSQRSANISGGEVKWLSEALDCKSKDRG

IMRMTAPVKLQGVWMRMAVTTRAMWLIEEYAMSDFRSVCRRQMELVMIMPHRDSTRKG

MRHNYNKLHLPTTNRPKIAHCMLFNQPHSPRSNSHSHPNPLKLHRRSHSHNRPRAYILITILPSKLKLRTHSQSHHNPLSRTSNSTPTNSFLMTS
SKPR

Table S4. List of proteins found by mass spectrometry following immunoprecipitation.

Immunoprecipitation (n = 2)			
PROTEIN NAME	Accession Number	Molecular Weight	Unique peptides count (Total spectrum count)
Protein [synthetic construct, MTALTND4]	AEX63613.1	16 kDa	IP1 = 2 (5)<br>IP2 = 2 (2)

Co-Immunoprecipitation *(Total spectrum count in the co-IP minus in the control; in bold are the peptides found in at least 2 out of 3 co-IPs)*					
PROTEIN NAME	Accession Number	Molecular Weight	Presence in Co-IP 1 Total spectrum count*	Presence in Co-IP 2 Total spectrum count*	Presence in Co-IP 3 Total spectrum count*
Echinoderm microtubule-associated protein-like 4 OS=Homo sapiens GN=EML4 PE=1 SV=3	Q9HC35	109 kDa	57	12	-
Protein scribble homolog OS=Homo sapiens GN=SCRIB PE=1 SV=4	Q14160	175 kDa	55	19	-
Dihydrolipoyllysine-residue succinyltransferase component of 2-oxoglutarate dehydrogenase complex, mitochondrial OS=Homo sapiens GN=DLST PE=1 SV=4	P36957	49 kDa	27	3	1
Cleavage stimulation factor subunit 2 tau variant OS=Homo sapiens GN=CSTF2T PE=1 SV=1	Q9H0L4	64 kDa	14	6	-
Elongation factor 1-delta OS=Homo sapiens GN=EEF1D PE=1 SV=5	EEF1D	31 kDa	3	4	4
Complement component 1 Q subcomponent-binding protein, mitochondrial OS=Homo sapiens GN=C1QBP PE=1 SV=1	Q07021	31 kDa	1	1	
Complement C1q subcomponent subunit C OS=Homo sapiens GN=C1QC PE=1 SV=3	P02747	26 kDa	1	1	
Complement C3 alpha chain (Fragment) OS=Oryctolagus cuniculus OX=9986 GN=C3 PE=2 SV=1	P12247	82 kDa	-	1	8
Complement C4-A OS=Homo sapiens GN=C4A PE=1 SV=2	P0C0L4	193 kDa	-	1	5
Elongation factor 1-beta OS=Homo sapiens GN=EEF1B2 PE=1 SV=3	P24534	25 kDa	-	3	1
60S ribosomal protein L31 OS=Homo sapiens GN=RPL31 PE=1 SV=1	P62899	14 kDa	-	2	1
Very long-chain specific acyl-CoA dehydrogenase, mitochondrial OS=Homo sapiens GN=ACADVL PE=1 SV=1	P49748	70 kDa	1	-	1
2-oxoglutarate dehydrogenase, mitochondrial OS=Homo sapiens GN=OGDH PE=1 SV=3	Q02218	116 kDa	48	-	-
Arf-GAP with coiled-coil, ANK repeat and PH domain-containing protein 2 OS=Homo sapiens GN=ACAP2 PE=1 SV=3	Q15057	88 kDa	29	-	-

Probable ATP-dependent RNA helicase DDX23 OS=Homo sapiens GN=DDX23 PE=1 SV=3	Q9BUQ8	96 kDa	3	-	-
Kinesin-like protein KIF2A OS=Homo sapiens GN=KIF2A PE=1 SV=3	O00139	80 kDa	6	-	-
Dihydrolipoyl dehydrogenase, mitochondrial OS=Homo sapiens GN=DLD PE=1 SV=2	P09622	54 kDa	17	-	-
TAR DNA-binding protein 43 OS=Homo sapiens GN=TARDBP PE=1 SV=1	Q13148	45 kDa	2	-	-
7-dehydrocholesterol reductase OS=Homo sapiens GN=DHCR7 PE=1 SV=1	Q9UBM7	54 kDa	2	-	-
ATP-dependent RNA helicase DDX19B OS=Homo sapiens GN=DDX19B PE=1 SV=1	Q9UMR2	54 kDa	2	-	-
Sideroflexin-3 OS=Homo sapiens GN=SFXN3 PE=1 SV=3	Q9BWM7	36 kDa	2	-	-
Nucleolar protein 16 OS=Homo sapiens GN=NOP16 PE=1 SV=2	Q9Y3C1	21 kDa	2	-	-
Actin-like protein 6A OS=Homo sapiens GN=ACTL6A PE=1 SV=1	O96019	47 kDa	2	-	-
GTP-binding protein SAR1a OS=Homo sapiens GN=SAR1A PE=1 SV=1	Q9NR31	22 kDa	2	-	-
MICOS complex subunit MIC19 OS=Homo sapiens GN=CHCHD3 PE=1 SV=1	Q9NX63	26 kDa	3	-	-
Cleavage stimulation factor subunit 3 OS=Homo sapiens GN=CSTF3 PE=1 SV=1	Q12996	83 kDa	3	-	-
WD repeat and HMG-box DNA-binding protein 1 OS=Homo sapiens GN=WDHD1 PE=1 SV=1	O75717	126 kDa	5	-	-
Mitochondrial chaperone BCS1 OS=Homo sapiens GN=BCS1L PE=1 SV=1	Q9Y276	48 kDa	9	-	-
RNA-binding protein 34 OS=Homo sapiens GN=RBM34 PE=1 SV=2	P42696	49 kDa	2	-	-
ATPase WRNIP1 OS=Homo sapiens GN=WRNIP1 PE=1 SV=2	Q96S55	72 kDa	3	-	-
Serine/threonine-protein phosphatase 2A 55 kDa regulatory subunit B alpha isoform OS=Homo sapiens GN=PPP2R2A PE=1 SV=1	P63151	52 kDa	2	-	-
Rho guanine nucleotide exchange factor 7 OS=Homo sapiens GN=ARHGEF7 PE=1 SV=2	Q14155	90 kDa	3	-	-
Cytochrome c1, heme protein, mitochondrial OS=Homo sapiens GN=CYC1 PE=1 SV=3	P08574	35 kDa	1	-	-
Protein KRI1 homolog OS=Homo sapiens GN=KRI1 PE=1 SV=3	Q8N9T8	83 kDa	1	-	-
Cleavage stimulation factor subunit 1 OS=Homo sapiens GN=CSTF1 PE=1 SV=1	Q05048	48 kDa	3	-	-
CD2-associated protein OS=Homo sapiens	Q9Y5K6	71 kDa			

GN=CD2AP PE=1 SV=1			4	-	-
Chromatin target of PRMT1 protein OS=Homo sapiens GN=CHTOP PE=1 SV=2	Q9Y3Y2	26 kDa	1	-	-
NADH dehydrogenase [ubiquinone] 1 alpha subcomplex subunit 10, mitochondrial OS=Homo sapiens GN=NDUFA10 PE=1 SV=1	O95299	41 kDa	2	-	-
Casein kinase I isoform alpha OS=Homo sapiens GN=CSNK1A1 PE=1 SV=2	P48729	39 kDa	2	-	-
Elongin-A OS=Homo sapiens GN=ELOA PE=1 SV=2	Q14241	90 kDa	1	-	-
Basic leucine zipper and W2 domain-containing protein 2 OS=Homo sapiens GN=BZW2 PE=1 SV=1	Q9Y6E2	48 kDa	1	-	-
Ribosome production factor 1 OS=Homo sapiens GN=RPF1 PE=1 SV=2	Q9H9Y2	40 kDa	1	-	-
Importin-8 OS=Homo sapiens GN=IPO8 PE=1 SV=2	O15397	120 kDa	2	-	-
ARF GTPase-activating protein GIT1 OS=Homo sapiens GN=GIT1 PE=1 SV=2	Q9Y2X7	84 kDa	2	-	-
NADH dehydrogenase [ubiquinone] 1 beta subcomplex subunit 10 OS=Homo sapiens GN=NDUFB10 PE=1 SV=3	O96000	21 kDa	1	-	-
Nuclear ubiquitous casein and cyclin-dependent kinase substrate 1 OS=Homo sapiens GN=NUCKS1 PE=1 SV=1	Q9H1E3	27 kDa	1	-	-
THO complex subunit 5 homolog OS=Homo sapiens GN=THOC5 PE=1 SV=2	Q13769	79 kDa	1	-	-
Fatty aldehyde dehydrogenase OS=Homo sapiens GN=ALDH3A2 PE=1 SV=1	P51648	55 kDa	1	-	-
High mobility group protein B1 OS=Homo sapiens GN=HMGB1 PE=1 SV=3	P09429	25 kDa	1	-	-
Arginine and glutamate-rich protein 1 OS=Homo sapiens GN=ARGLU1 PE=1 SV=1	Q9NWB6	33 kDa	1	-	-
Non-histone chromosomal protein HMG-14 OS=Homo sapiens GN=HMGN1 PE=1 SV=3	P05114	11 kDa	1	-	-
Protein S100-P OS=Homo sapiens GN=S100P PE=1 SV=2	P25815	10 kDa	1	-	-
Protein TFG OS=Homo sapiens GN=TFG PE=1 SV=2	Q92734	43 kDa	3	-	-
Polyglutamine-binding protein 1 OS=Homo sapiens GN=PQBP1 PE=1 SV=1	O60828	30 kDa	1	-	-
SWI/SNF-related matrix-associated actin-dependent regulator of chromatin subfamily B member 1 OS=Homo sapiens GN=SMARCB1 PE=1 SV=2	Q12824	44 kDa			

			1	-	-
Ubiquitin-conjugating enzyme E2 N OS=Homo sapiens GN=UBE2N PE=1	P61088	17 kDa	1	-	-
Protein FRG1 OS=Homo sapiens GN=FRG1 PE=1 SV=1	Q14331	29 kDa	1	-	-
Cilia- and flagella-associated protein 20 OS=Homo sapiens GN=CFAP20 PE=1 SV=1	Q9Y6A4	23 kDa	1	-	-
Leucine-rich repeat and WD repeat-containing protein 1 OS=Homo sapiens GN=LRWD1 PE=1 SV=2	Q9UFC0	71 kDa	1	-	-
Uncharacterized protein C11orf98 OS=Homo sapiens GN=C11orf98 PE=4 SV=1	E9PRG8	14 kDa	1	-	-
Uncharacterized protein C10orf88 OS=Homo sapiens GN=C10orf88 PE=1 SV=2	Q9H8K7	49 kDa	2	-	-
TREMBL:Q1RMK2 (Bos taurus) IGHM protein	Q1RMK2	65 kDa	-	10	-
Glyceraldehyde-3-phosphate dehydrogenase OS=Homo sapiens GN=GAPDH PE=1 SV=3	P04406	36 kDa	-	2	-
Desmoplakin OS=Homo sapiens GN=DSP PE=1 SV=3	P15924	332 kDa	-	4	-
MICOS complex subunit MIC60 OS=Homo sapiens GN=IMMT PE=1 SV=1	Q16891	84 kDa	-	2	-
Hornerin OS=Homo sapiens GN=HRNR PE=1 SV=2	Q86YZ3	282 kDa	-	3	-
T-complex protein 1 subunit zeta OS=Homo sapiens GN=CCT6A PE=1 SV=3	P40227	58 kDa	-	2	-
Delta-1-pyrroline-5-carboxylate dehydrogenase, mitochondrial OS=Homo sapiens GN=ALDH4A1 PE=1 SV=3	P30038	62 kDa	-	3	-
L-lactate dehydrogenase A chain OS=Homo sapiens GN=LDHA PE=1 SV=2	P00338	37 kDa	-	2	-
40S ribosomal protein S28 OS=Homo sapiens GN=RPS28 PE=1 SV=1	P62857	8 kDa	-	4	-
Peptidyl-prolyl cis-trans isomerase A OS=Homo sapiens GN=PPIA PE=1 SV=2	P62937	18 kDa	-	1	-
Alpha-actinin-4 OS=Homo sapiens GN=ACTN4 PE=1 SV=2	O43707 (+1)	105 kDa	-	2	-
Activated RNA polymerase II transcriptional coactivator p15 OS=Homo sapiens GN=SUB1 PE=1 SV=3	P53999	14 kDa	-	2	-
SWISS-PROT:P02662 Alpha-S1-casein - Bos taurus (Bovine).	P02662	23 kDa	-	2	-
Histidine-rich glycoprotein (Fragment) OS=Oryctolagus cuniculus OX=9986 GN=HRG PE=1 SV=1	Q28640	59 kDa	-	-	13
Transthyretin OS=Oryctolagus cuniculus OX=9986 GN=TTR PE=1 SV=1	P07489	14 kDa			

			-	-	8
Serotransferrin OS=Oryctolagus cuniculus OX=9986 GN=TF PE=1 SV=4	P19134	77 kDa	-	-	3
Cluster of Tubulin beta chain OS=Homo sapiens OX=9606 GN=TUBB PE=1 SV=2 (P07437)	P07437 [18]	50 kDa	-	-	11
Cluster of 60S acidic ribosomal protein P0 OS=Homo sapiens OX=9606 GN=RPLP0 PE=1 SV=1 (P05388)	P05388 [2]	34 kDa	-	-	6
Elongation factor 1-gamma OS=Homo sapiens OX=9606 GN=EEF1G PE=1 SV=3	P26641	50 kDa	-	-	4
Nucleophosmin OS=Homo sapiens OX=9606 GN=NPM1 PE=1 SV=2	P06748	33 kDa	-	-	6
Extracellular superoxide dismutase [Cu-Zn] OS=Oryctolagus cuniculus OX=9986 GN=SOD3 PE=2 SV=2	P41975	26 kDa	-	-	2

*Only results with peptide thresholds 95,0% minimum and protein thresholds 99,0% minimum and 2 peptides minimum are presented.

Table S5. List of proteins found by mass spectrometry following pull down assay.

PROTEIN NAME	Accession Number	Molecular Weight	Pull down 1 (HeLa) Unique spectrum count	Pull down 2 (HeLa) Unique spectrum count	Pull down (HEK-293T) Unique spectrum count
Cluster of Tubulin beta chain OS=Homo sapiens OX=9606 GN=TUBB PE=1 SV=2 (P07437)	P07437	50 kDa	48	24	21
Glutathione S-transferase LANCL1 OS=Homo sapiens OX=9606 GN=LANCL1 PE=1 SV=1	O43813	45 kDa	22	5	12
Nucleophosmin OS=Homo sapiens OX=9606 GN=NPM1 PE=1 SV=2	P06748	33 kDa	14	6	6
Elongation factor 1-gamma OS=Homo sapiens OX=9606 GN=EEF1G PE=1 SV=3	P26641	50 kDa	13	2	8
**Complement component 1 Q subcomponent-binding protein, mitochondrial OS=Homo sapiens OX=9606 GN=C1QBP PE=1 SV=1**	**Q07021**	**31 kDa**	**12**	**15**	**16**
Nucleosome assembly protein 1-like 1 OS=Homo sapiens OX=9606 GN=NAP1L1 PE=1 SV=1	P55209	45 kDa	10	8	11
Protein phosphatase 1G OS=Homo sapiens OX=9606 GN=PPM1G PE=1 SV=1	O15355	59 kDa	9	5	7
Protein SET OS=Homo sapiens OX=9606 GN=SET PE=1 SV=3	Q01105	33 kDa	9	3	3
Nucleosome assembly protein 1-like 4 OS=Homo sapiens OX=9606 GN=NAP1L4 PE=1 SV=1	Q99733	43 kDa	8	2	3
Proliferating cell nuclear antigen OS=Homo sapiens OX=9606 GN=PCNA PE=1 SV=1	P12004	29 kDa	7	2	3
Acidic leucine-rich nuclear phosphoprotein 32 family member B OS=Homo sapiens OX=9606 GN=ANP32B PE=1 SV=1	Q92688	29 kDa	6	2	6
40S ribosomal protein S18 OS=Homo sapiens OX=9606 GN=RPS18 PE=1 SV=3	P62269	18 kDa	6	6	8

40S ribosomal protein S13 OS=Homo sapiens OX=9606 GN=RPS13 PE=1 SV=2	P62277	17 kDa	5	7	5
Cluster of Heterogeneous nuclear ribonucleoprotein H OS=Homo sapiens OX=9606 GN=HNRNPH1 PE=1 SV=4 (P31943)	P31943	49 kDa	5	6	8
ATP synthase subunit beta, mitochondrial OS=Homo sapiens OX=9606 GN=ATP5F1B PE=1 SV=3	P06576	57 kDa	5	1	4
40S ribosomal protein S19 OS=Homo sapiens OX=9606 GN=RPS19 PE=1 SV=2	P39019	16 kDa	5	5	2
Cluster of Tubulin alpha-1B chain OS=Homo sapiens OX=9606 GN=TUBA1B PE=1 SV=1 (P68363)	P68363	50 kDa	5	11	11
Elongation factor 1-beta OS=Homo sapiens OX=9606 GN=EEF1B2 PE=1 SV=3	P24534	25 kDa	4	2	4
Casein kinase II subunit alpha OS=Homo sapiens OX=9606 GN=CSNK2A1 PE=1 SV=1	P68400	45 kDa	4	1	-
40S ribosomal protein S3 OS=Homo sapiens OX=9606 GN=RPS3 PE=1 SV=2	P23396	27 kDa	4	2	7
Endoplasmic reticulum chaperone BiP OS=Homo sapiens OX=9606 GN=HSPA5 PE=1 SV=2	P11021	72 kDa	4	11	15
ATP synthase subunit alpha, mitochondrial OS=Homo sapiens OX=9606 GN=ATP5F1A PE=1 SV=1	P25705	60 kDa	3	3	4
60S ribosomal protein L5 OS=Homo sapiens OX=9606 GN=RPL5 PE=1 SV=3	P46777	34 kDa	3	3	6
40S ribosomal protein S10 OS=Homo sapiens OX=9606 GN=RPS10 PE=1 SV=1	P46783	19 kDa	3	3	3
Y-box-binding protein 1 OS=Homo sapiens OX=9606 GN=YBX1 PE=1 SV=3	P67809	36 kDa	3	7	12
Elongation factor Tu, mitochondrial OS=Homo sapiens OX=9606 GN=TUFM PE=1 SV=2	P49411	50 kDa	3	2	2
Cluster of 40S ribosomal protein S4, X isoform OS=Homo sapiens OX=9606 GN=RPS4X PE=1 SV=2 (P62701)	P62701	30 kDa	2	7	10
40S ribosomal protein S11 OS=Homo sapiens OX=9606 GN=RPS11 PE=1 SV=3	P62280	18 kDa	2	4	6
40S ribosomal protein S16 OS=Homo sapiens OX=9606 GN=RPS16 PE=1 SV=2	P62249	16 kDa	2	4	7
40S ribosomal protein S5 OS=Homo sapiens OX=9606 GN=RPS5 PE=1 SV=4	P46782	23 kDa	2	8	11
60S ribosomal protein L23 OS=Homo sapiens OX=9606 GN=RPL23 PE=1 SV=1	P62829	15 kDa	2	3	2
Signal recognition particle 14 kDa protein OS=Homo sapiens OX=9606 GN=SRP14 PE=1 SV=2	P37108	15 kDa	2	1	-
60S ribosomal protein L27a OS=Homo sapiens OX=9606 GN=RPL27A PE=1 SV=2	P46776	17 kDa	2	2	4
Alpha-S1-casein OS=Bos taurus OX=9913 GN=CSN1S1 PE=1 SV=2	P02662	25 kDa	2	4	3
40S ribosomal protein S20 OS=Homo sapiens OX=9606 GN=RPS20 PE=1 SV=1	P60866	13 kDa	2	2	-
40S ribosomal protein S14 OS=Homo sapiens OX=9606 GN=RPS14 PE=1 SV=3	P62263	16 kDa	2	2	3

*Only results with peptide thresholds 95,0% minimum and protein thresholds 99,0% minimum and 2 peptides minimum are presented

Table S6. Dose-dependent impact of MTALTND4 on routine respiration of intact HeLa and HEK-293T cells.

0.05 > *p* ≤ 0.09; **p* ≤ 0.05; ***p* ≤ 0.01; ****p* ≤ 0.001. Data are reported separately and also as mean ± standard error of the mean (sem). Data refer to Fig. 3A,B.

Table S6			MTALTND4 concentration										
**cell type**	**ID**	**treatment**	**0.1 µM**	**1 µM**	**5 µM**	**10 µM**	**15 µM**	**20 µM**	**25 µM**	**30 µM**	**35 µM**	**40 µM**	**45 µM**
hek	*1hk*	*ctrl*	1	1	1	1	1	1	1	1	1	1	1
hek	*1hk*	*pept*	0.979	0.961	0.971	0.939	0.902	0.892	0.754	0.663	0.630	0.581	0.511
hek	*2hk*	*pept*	0.989	1.036	0.927	0.937	0.901	0.740	0.739	0.720	0.597	0.599	0.562
hek	*2hk*	*ctrl*	1	1	1	1	1	1	1	1	1	1	1
hek	*3hk*	*pept*	0.994	0.959	1.001	0.990	0.948	0.697	0.663	0.689	0.652	0.548	0.559
hek	*3hk*	*ctrl*	1	1	1	1	1	1	1	1	1	1	1
hela	*1hl*	*ctrl*	1	1	1	1	1	1	1	1	*na*	*na*	*na*
hela	*1hl*	*pept*	0.998	0.970	0.742	0.500	0.380	0.350	0.301	0.148			
hela	*0hl*	*ctrl*	1	1	1	1	1	1	1	1			
hela	*0hl*	*pept*	0.946	0.899	0.604	0.384	0.270	0.171	0.101	0.097			
hela	*2hl*	*ctrl*	1	1	1	1	1	1	1	1			
hela	*2hl*	*pept*	1.004	0.899	0.677	0.551	0.475	0.431	0.299	0.215			
hela	*3hl*	*ctrl*	1	1	1	1	1	1	1	1			
hela	*3hl*	*pept*	1.039	1.016	0.848	0.661	0.560	0.506	0.466	0.271			
HEK-293T	*Stats*	*ctrl (mean ± sem)*	1 ± 0	1 ± 0	1 ± 0	1 ± 0	1 ± 0	1 ± 0	1 ± 0	1 ± 0	1 ± 0	1 ± 0	1 ± 0
		pept (mean ± sem)	0.987 ± 0.004	0.985 ± 0.025	0.967 ± 0.021	0.955 ± 0.017	0.917 ± 0.016	0.776 ± 0.059	0.719 ± 0.028	0.691 ± 0.016	0.626 ± 0.016	0.576 ± 0.015	0.544 ± 0.017
		transformation	*none*	*none*	*none*	*none*	*none*	*none*	*none*	*none*	*none*	*none*	*none*
		:treatment	*t*=-2.95, *P*=0.098	*t*=-0.58, *P*=0.62	*t*=-1.56, *P*=0.259	*t*=-2.59, *P*=0.122	***t*=-5.31, *P*=0.0336***	*t*=-3.79, *P*=0.063·	***t*=-9.99, *P*=9.856e-03****	***t*=-18.84, *P*=2.8e-03****	***t*=-23.33, *P*=1.8e-03****	***t*=-28.75, *P*=1.2e-03****	***t*=-27.56, *P*=1.3e-03****
		p adjusted	0.39272	0.6205	0.5184	0.39272	0.20196	0.3154	0.068992·	**0.022448***	**0.016479***	**0.013277***	**0.013277***
HeLa	*Stats*	*ctrl (mean ± sem)*	1 ± 0	1 ± 0	1 ± 0	1 ± 0	1 ± 0	1 ± 0	1 ± 0	1 ± 0	*na*	*na*	*na*
		pept (mean ± sem)	0.997 ± 0.019	0.946 ± 0.029	0.718 ± 0.052	0.524 ± 0.058	0.421 ± 0.062	0.364 ± 0.072	0.292 ± 0.075	0.183 ± 0.038			
		transformation	*none*	*none*	*none*	*none*	*none*	*none*	*none*	*none*			
		:treatment	*t*=-0.16, *P*= 0.879	*t*=-1.88, *P*=0.156	***t*=-5.45, *P*=1.2e-02***	***t*=-8.27, *P*=3.6e-03****	***t*=-9.27, *P*=2.6e-03****	***t*=-8.84, *P*=3e-03****	***t*=-9.5, *P*=2.4e-03****	***t*=-21.5, *P*=2.2e-04*****			
		p adjusted	0.8798	0.3134	**0.03642***	**0.017269***	**0.017269***	**0.017269***	**0.017269***	**0.0017608****			

Table S7. Impact of 10 µM MTALTND4 on mitochondrial respiration of intact HeLa and HEK-293T cells.
0.05 > $p \leq 0.09$; *$p \leq 0.05$; **$p \leq 0.01$; ***$p \leq 0.001$. Data are reported separately and also as mean ± standard error of the mean (sem). Data refer to Fig. 3C,D,E.

cell type	ID	treatment	ce_R	ce-pept_R	ce-pept_L	ce-pept_E		SRC (E-R)	
hek	*1hk*	*ctrl*	1	0.908	0.249	3.687			2.687
hek	*1hk*	*pept*	1	0.962	0.175	2.988			1.988
hek	*2hk*	*ctrl*	1	0.972	0.219	3.007			2.007
hek	*2hk*	*pept*	1	0.950	0.210	2.258			1.258
hek	*3hk*	*ctrl*	1	0.971	0.219	3.826			2.826
hek	*3hk*	*pept*	1	0.894	0.195	1.924			0.924
hek	*4hk*	*ctrl*	1	0.941	0.241	3.658			2.658
hek	*4hk*	*pept*	1	0.808	0.220	2.255			1.255
hek	*5hk*	*ctrl*	1	0.987	0.295	3.520			2.520
hek	*5hk*	*pept*	1	0.825	0.230	2.224			1.224
hek	*6hk*	*pept*	1	0.886	0.184	3.264			2.264
hek	*6hk*	*ctrl*	1	0.956	0.219	4.109			3.109
hela	*1hl*	*ctrl*	1	0.976	0.241	2.705			1.705
hela	*1hl*	*pept*	1	0.464	0.138	0.592			-0.408
hela	*2hl*	*ctrl*	1	0.867	0.140	2.130			1.130
hela	*2hl*	*pept*	1	0.786	0.112	1.967			0.967
hela	*3hl*	*pept*	1	0.283	0.066	0.339			-0.661
hela	*3hl*	*ctrl*	1	0.566	0.062	1.647			0.647
hela	*4hl*	*ctrl*	1	0.951	0.126	2.480			1.480
hela	*4hl*	*pept*	1	0.692	0.102	1.582			0.582
hela	*5hl*	*ctrl*	1	0.730	0.114	1.854			0.854
hela	*5hl*	*pept*	1	0.635	0.099	1.254			0.254
hela	*6hl*	*pept*	1	0.557	0.125	0.877			-0.123
hela	*6hl*	*ctrl*	1	0.803	0.221	1.490			0.490
hela	*x1hl*	*ctrl*	1	0.878					
hela	*x1hl*	*pept*	1	0.460					
hela	*x2hl*	*ctrl*	1	0.922	*na*	*na*			*na*
hela	*x2hl*	*pept*	1	0.459					
hela	*x3hl*	*ctrl*	1	0.946					
hela	*x3hl*	*pept*	1	0.389					
		ctrl (mean ± sem)	1 ± 0	0.956 ± 0.012	0.241 ± 0.012	3.634 ± 0.15		*HEK293*	2.634 ± 0.15
		pept (mean ± sem)	1 ± 0	0.888 ± 0.026	0.202 ± 0.009	2.486 ± 0.212			1.486 ± 0.212
HEK-	*Stats*	*transformation*	*none*	*none*	*none*	*none*			1.051 ± 0.195

293T		*:treatment*		t=2.15, P=0.084·	**t=3.56, P=0.016***	**t=5.97, P=0.0018****
		p adjusted		0.08412	**0.03232***	**0.005628****
HELA	*Stats*	*ctrl (mean ± sem)*	1 ± 0	0.849 ± 0.044	0.151 ± 0.028	2.051 ± 0.195
		pept (mean ± sem)	1 ± 0	0.525 ± 0.053	0.107 ± 0.01	1.102 ± 0.251
		transformation	*none*	*none*	*none*	*none*
		:treatment		**t=5.58, P=5.1e-04*****	t=2.38, P=0.062·	**t=3.4, P=0.019***
		p adjusted		**0.0015507****	0.06281·	**0.03848***

HELA	0.102 ± 0.251
LMM	
transformation	*none*
:cell type	**F1,12=47.68, P=1.639e-05 *****
:treatment	**F1,12=45.99, P=1.952e-05 *****
:interaction	F1,12=0.4165, P=0.5308

Table S8. Impact of 10 µM MTALTND4 on mitochondrial respiration of permeabilized HEK-293T cells.

$0.05 > p \le 0.09$; *$p \le 0.05$; **$p \le 0.01$; ***$p \le 0.001$. Data are reported separately and also as mean ± standard error of the mean (sem). Data refer to Fig. 3F.

cell type	ID	treatment	ce_R	CI_L	CI_P	c_P	CI+II_P	CI+II-pept_P	CI+II-pept_E	Rot	CIV-pept_E
hek	*1hk*	*pept*	0.386	0.167	0.595	0.637	1	0.303	0.399	0.007	0.643
hek	*1hk*	*ctrl*	1.470	0.521	1.374	1.411	1	0.822	1.995	0.023	9.993
hek	*2hk*	*pept*	0.505	0.174	0.678	0.696	1	0.072	0.046	0.027	1.016
hek	*2hk*	*ctrl*	0.806	0.302	0.891	0.932	1	0.773	1.017	0.026	4.656
hek	*3hk*	*pept*	0.682	0.267	0.643	0.737	1	0.538	0.661	0.001	2.014
hek	*3hk*	*ctrl*	1.034	0.364	0.889	0.930	1	0.855	1.259	0.042	7.802
hek	*4hk*	*ctrl*	0.536	0.234	0.636	0.649	1	0.978	1.923	-0.003	2.174
hek	*4hk*	*pept*	0.495	0.180	0.716	0.706	1	0.640	0.992	0.004	1.576
hek	*5hk*	*ctrl*	0.612	0.324	0.575	0.597	1	0.940	1.453	0.006	2.452
hek	*5hk*	*pept*	0.926	0.435	0.699	0.730	1	0.320	0.449	0.013	1.969
hek	*6hk*	*pept*	0.418	0.124	0.811	0.762	1	0.536	0.700	0.001	0.883
hek	*6hk*	*ctrl*	0.387	0.094	0.796	0.827	1	1.021	1.149	0.026	1.219
HEK-293T	*Stats*	*ctrl (mean ± sem)*	0.807 ± 0.161	0.307 ± 0.058	0.86 ± 0.116	0.891 ± 0.119	1 ± 0	0.898 ± 0.039	1.466 ± 0.167	0.02 ± 0.007	4.716 ± 1.428
		pept (mean ± sem)	0.569 ± 0.083	0.225 ± 0.046	0.69 ± 0.03	0.711 ± 0.018	1 ± 0	0.402 ± 0.085	0.541 ± 0.131	0.009 ± 0.004	1.35 ± 0.238
		transformation	*none*	*none*	*none*	*none*	*none*	*none*	*none*	*none*	*none*
		:treatment	t=1.21, P=0.27	t=1.25, P=0.26	t=1.24, P=0.27	t=1.36, P=0.23		**t=8.01, P=4.8e-04*****	**t=5.69, P=0.0023****	t=1.39, P=0.22	t=2.25, P=0.073·
		p adjusted	1	1	1	1		**0.003912****	**0.016331***	1	0.44346

Table S9. Impact of MTALTND4 on intact HEK-293T cell's lactic fermentation, antioxidant capacity, ATP content and hydrogen peroxide efflux rate. $\cdot 0.05 > p \le 0.09$; $*p \le 0.05$; $**p \le 0.01$; $***p \le 0.001$. Data are reported separately and also as mean ± standard error of the mean (sem). Data refer to Fig. 3G,H,I,L.

Table S9			U·mg−1		ol H2O2·mg−1·min−1	nmol ATP·mg−1
**Cell-type**	**ID**	**Treatment**	**LDH**	**CAT**	**H2O2 efflux pmol**	**ATP content**
Hek	*1hk*	*ctrl*	0.427	3.249	9.948	1.359
Hek	*2hk*	*ctrl*	0.318	2.729	8.258	1.296
Hek	*3hk*	*ctrl*	0.384	2.951	8.807	1.459
Hek	*4hk*	*ctrl*	0.310	2.728	7.109	1.200
Hek	*5hk*	*ctrl*	0.321	2.761	7.835	1.549
Hek	*1hk*	*pept-30uM*	0.305	3.154	14.375	1.827
Hek	*2hk*	*pept-30uM*	0.289	1.876	12.150	1.845
Hek	*3hk*	*pept-30uM*	0.346	3.023	15.021	1.940
Hek	*4hk*	*pept-30uM*	0.292	2.385	9.361	1.593
Hek	*5hk*	*pept-30uM*	0.391	2.873	9.861	2.271
HEK-293T	*Stats*	*ctrl (mean ± sem)*	0.352 ± 0.023	2.884 ± 0.1	8.392 ± 0.478	1.373 ± 0.061
		pept (mean ± sem)	0.325 ± 0.02	2.662 ± 0.236	12.154 ± 1.145	1.895 ± 0.11
		transformation	*none*	*none*	*none*	*none*
		:treatment	*v*=11, *P*=0.437	*t*=1.25, *P*=0.279	***t*=-4.9, *P*=0.008****	***t*=-9.39, *P*=0.00071*****
		p adjusted	0.558	0.558	**0.024***	**0.00284****

Table S10. Dose- and time-dependent impact of MTALTND4 on HeLa cells proliferation and viability.
0.05 > $p \leq 0.09$; *$p \leq 0.05$; **$p \leq 0.01$; ***$p \leq 0.001$. Data are reported separately and also as mean ± standard error of the mean (sem). Data refer to Fig. 3M,N.

cell type	ID	treatment	time	proliferation (Mx cells)	viability (%)
Hela	*1hl*	*ctrl*	24h	27500	83.33
Hela	*2hl*	*ctrl*	24h	19500	85.71
Hela	*3hl*	*ctrl*	24h	45500	93.33
Hela	*4hl*	*ctrl*	24h	34500	*na*
Hela	*5hl*	*ctrl*	24h	31000	*na*
Hela	*1hl*	*ctrl*	48h	176000	100.00
Hela	*2hl*	*ctrl*	48h	130000	100.00
Hela	*3hl*	*ctrl*	48h	115000	95.83
Hela	*4hl*	*ctrl*	48h	109000	*na*
Hela	*5hl*	*ctrl*	48h	106000	*na*
Hela	*1hl*	*ctrl*	72h	495000	90.00
Hela	*2hl*	*ctrl*	72h	172500	84.15
Hela	*3hl*	ctrl	72h	310000	97.64
Hela	*4hl*	ctrl	72h	153500	*na*
Hela	*5hl*	ctrl	72h	280000	*na*
Hela	*1hl*	0,1µM	24h	27500	71.43
Hela	*2hl*	0,1µM	24h	61750	95.00
Hela	*3hl*	0,1µM	24h	19500	100.00
Hela	*4hl*	0,1µM	24h	32500	*na*
Hela	*5hl*	0,1µM	24h	30000	*na*
Hela	*1hl*	0,1µM	48h	143000	89.66
Hela	*2hl*	0,1µM	48h	87500	100.00
Hela	*3hl*	0,1µM	48h	62500	96.15
Hela	*4hl*	0,1µM	48h	106500	*na*
Hela	*5hl*	0,1µM	48h	94000	*na*
Hela	*1hl*	0,1µM	72h	412500	92.78
Hela	*2hl*	0,1µM	72h	252500	99.02

Hela	*3hl*	0,1µM	72h	362500	98.64
Hela	*4hl*	0,1µM	72h	210500	*na*
Hela	*5hl*	0,1µM	72h	288500	*na*
Hela	*1hl*	10µM	24h	60500	100.00
Hela	*2hl*	10µM	24h	42250	92.86
Hela	*3hl*	10µM	24h	16500	100.00
Hela	*4hl*	10µM	24h	43000	*na*
Hela	*5hl*	10µM	24h	38500	*na*
Hela	*1hl*	10µM	48h	143000	89.66
Hela	*2hl*	10µM	48h	82500	94.29
Hela	*3hl*	10µM	48h	55000	91.67
Hela	*4hl*	10µM	48h	61500	*na*
Hela	*5hl*	10µM	48h	70500	*na*
Hela	*1hl*	10µM	72h	291500	97.40
Hela	*2hl*	10µM	72h	280000	97.39
Hela	*3hl*	10µM	72h	260000	98.11
Hela	*4hl*	10µM	72h	226500	*na*
Hela	*5hl*	10µM	72h	214000	*na*
Hela	*1hl*	30µM	24h	27500	83.33
Hela	*2hl*	30µM	24h	39000	92.31
Hela	*3hl*	30µM	24h	9750	42.86
Hela	*4hl*	30µM	24h	43500	*na*
Hela	*5hl*	30µM	24h	21500	*na*
Hela	*1hl*	30µM	48h	77000	93.33
Hela	*2hl*	30µM	48h	45000	90.00
Hela	*3hl*	30µM	48h	47500	82.61
Hela	*4hl*	30µM	48h	77500	*na*
Hela	*5hl*	30µM	48h	62000	*na*
Hela	*1hl*	30µM	72h	346500	94.64
Hela	*2hl*	30µM	72h	75000	85.71
Hela	*3hl*	30µM	72h	87500	89.74
Hela	*4hl*	30µM	72h	288000	*na*
Hela	*5hl*	30µM	72h	101000	*na*
		ctrl	24h	31600 ± 4273.172	87.457 ± 3.016
		0,1µM		34250 ± 7212.836	88.81 ± 8.809
		10µM		40150 ± 7031.003	97.62 ± 2.38
		30µM		28250 ± 6068.361	72.833 ± 15.209

Stats	*LMM*	*transformation*		*none*	*none*
		:treatment		$F_{3,15}=0.82$, $P=0.5025$	$F_{3,12}=1.95$, $P=0.1745$
		ctrl	48h	127200 ± 12881.77	98.61 ± 1.39
		0,1µM		98700 ± 13193.37	95.27 ± 3.017
		10µM		82500 ± 15813.76	91.873 ± 1.34
		30µM		61800 ± 6943.702	88.647 ± 3.168
		transformation		*none*	*none*
		:treatment		**$F_{3,15}=18.83$, $P=2.39e-05$*****	**$F_{3,9}=5.22$, $P=0.023$***
		0,1µM - ctrl		**0.004587****	0.60412
		10µM - ctrl		**3.34e-06*****	0.05639·
		30µM - ctrl		**2.12e-12*****	**0.00107****
		10µM - 0,1µM		0.071642·	0.60412
		30µM - 0,1µM		**0.000163*****	0.05639·
		30µM - 10µM		**0.042696***	0.60412
		ctrl	72h	282200 ± 61097.995	90.597 ± 3.906
		0,1µM		305300 ± 36620.213	96.813 ± 2.02
		10µM		254400 ± 14956.102	97.633 ± 0.238
		30µM		179600 ± 57099.781	90.03 ± 2.582
		transformation		*none*	*none*
		:treatment		$F_{3,15}=2.86$, $P=0.071$·	$F_{3,9}=3.72$, $P=0.054$·
