# Supplementary material for: A small protein coded within the mitochondrial canonical gene nd4 regulates mitochondrial bioenergetics
Source: BMC Biol. 2023 May 18;21:111. doi: 10.1186/s12915-023-01609-y (PMC10193809; doi:10.1186/s12915-023-01609-y)
Supplement: Supplementary file 2 — Additional file 2: Figure S1. Using the PepQuery tool to interrogate spectrums from published proteome datasets. Figure S2. Putative post-translational modifications of MTALTND4. O-Glyc: putative O-glycosylation; P: putative phosphorylation. PTM prediction were done using webservers reviewed by [75], only scores with 80% probabilities were retained. Figure S3. Specificity of the anti-MTALTND4 antibody and detection of putative post-translational modificationsof MTALTND4. A. Complete western blot of the antibody on a cell lysate. B. Specificity of anti-MTALTND4 and PTMs. Deglyc: deglycosylationnon-treated cells,treated cells and fetuin positive control are shown); Dephosphos: dephosphorylationnon-treated cells andtreated cells are shown). C. Apparent molecular weight of MTALTND4 exposed to different concentrations of denaturing agent β-Mercaptoéthanol and different heating times at 95˚C. A = 5 minutes, B = 30 minutes and C = 3 hours. 1 = 0% β-Mercaptoethanol, 2 = 5% β-Mercaptoethanol and 3 = 10% β-Mercaptoethanol. D. Self-association of MTALTND4: 3 µg of synthetic peptide were incubated at 30 °C for 10 min in the presence of 0%, 0.5% and 1% paraformaldehyde, quenched with LSB, separated by tricine-SDS-PAGE and visualized with Coomassie Imperial Protein stain. Arrowheads indicate main cross-linked products. Figure S4. Comparison of MTALTND4 peptide encoded in mitochondrial DNAand hypothetically in nuclear DNA that have been transferred from mtDNAthrough evolution. Only sequences with >50% identities and complete without stop codons are shown. Figure S5. Multiple MTALTND4 peptide sequence alignments. A. In 15 mammal species: human, chimpanzee, bonobo, gorilla, orangutan, mouse, rat, naked mole rat, dog, cow, zebrafish, lion, bear, horse, dolphin. B. In the genus Homo: Homo heidelbergensis; Homo sapiens neanderthalensis; Homo sapiens neanderthalensis; Homo sapiens neanderthalensis; Homo sapiens neanderthalensis; Homo sapiens neanderthalensis; modern human, chimpanzee. * [file 12915_2023_1609_MOESM2_ESM.pdf]

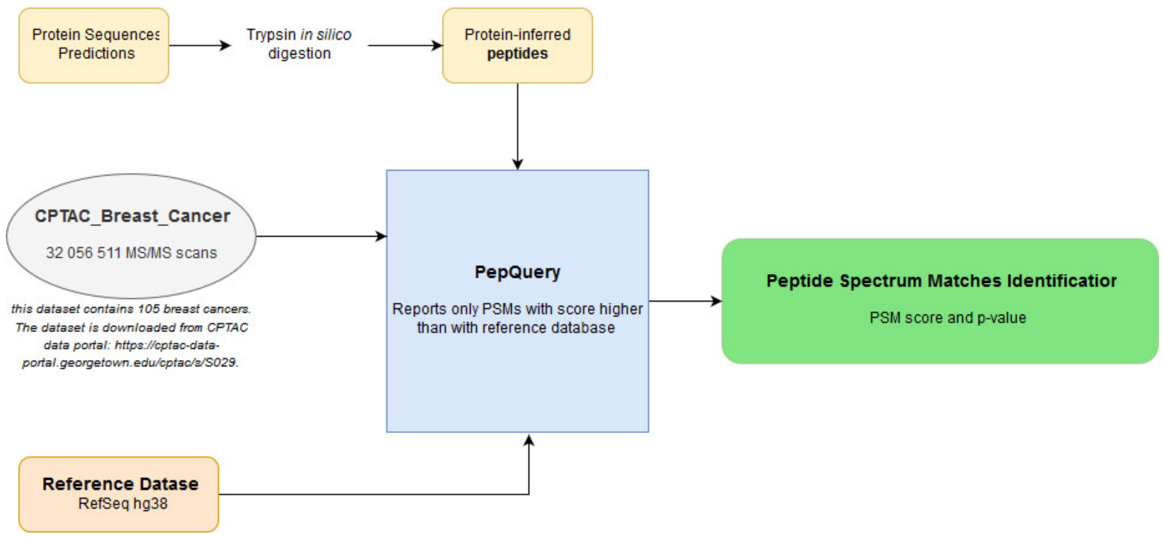

Figure S1. Using the PepQuery tool to interrogate spectrums from published proteome datasets.

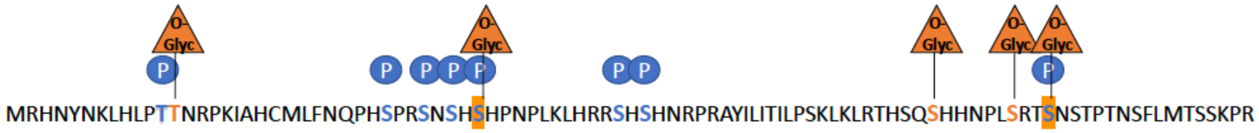

Figure S2. Putative post-translational modifications of MTALTND4. O-Glyc: putative O-glycosylation; P: putative phosphorylation. PTM prediction were done using webservers reviewed by [75], only scores with 80% probabilities were retained.

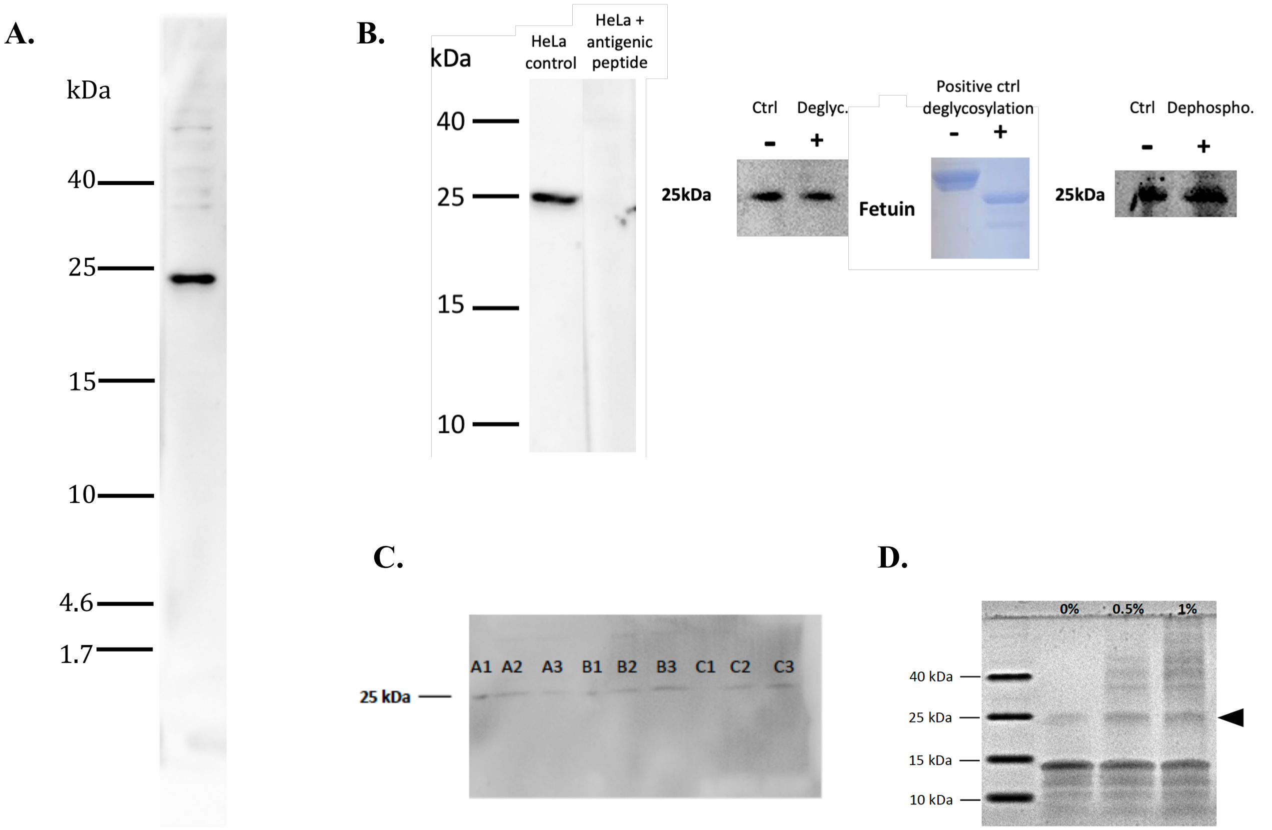

Figure S3. Specificity of the anti-MTALTND4 antibody and detection of putative post-translational modifications (PTMs) of MTALTND4. **A.** Complete western blot of the antibody on a cell lysate (Fig. 2B). **B.** Specificity of anti-MTALTND4 and PTMs. Deglyc: deglycosylation (whole cell lysates were treated with a complete protein deglycosylation mix II kit [NEB, Cat# P6044] following the manufacturer's protocol: (-) non-treated cells, (+) treated cells and fetuin positive control are shown); Dephosphos: dephosphorylation (whole cell lysates were treated with a lambda protein phosphatase [NEB, P0753S] following the manufacturer's protocol: (-) non-treated cells and (+) treated cells are shown). **C.** Apparent molecular weight of MTALTND4 exposed to different concentrations of denaturing agent ***β***-Mercaptoéthanol and different heating times at 95˚C. A = 5 minutes, B = 30 minutes and C = 3 hours. 1 = 0% ***β***-Mercaptoethanol, 2 = 5% ***β***-Mercaptoethanol and 3 = 10% ***β***-Mercaptoethanol. **D.** Self-association of MTALTND4: 3 µg of synthetic peptide were incubated at 30 °C for 10 min in the presence of 0%, 0.5% and 1% paraformaldehyde, quenched with LSB, separated by tricine-SDS-PAGE and visualized with Coomassie Imperial Protein stain. Arrowheads indicate main cross-linked products.

```
Query: None Query ID: lcl|Query_3741 Length: 99

>Homo sapiens mitochondrion, complete genome
Sequence ID: NC_012920.1 Length: 16569
Range 1: 11557 to 11853

Score:152 bits(383), Expect:3e-42,
Method:Compositional matrix adjust.,
Identities:98/99(99%), Positives:99/99(100%), Gaps:0/99(0%)

Query  1      MRHNYNKLHLPTTNRPKIAHCMLFnqphsprsnshshpnpLKLHRRSHSHNRPRAYILIT  60
              MRHNYNKLHLPTTNRPKIAHC+LFNQPHSPRSNSHSHPNPLKLHRRSHSHNRPRAYILIT
Sbjct  11557  MRHNYNKLHLPTTNRPKIAHCILFNQPHSPRSNSHSHPNPLKLHRRSHSHNRPRAYILIT  11736

Query  61     ILPSKLKLRTHSQSHHNPLSRTSNSTPTNSFLMTSSKPR  99
              ILPSKLKLRTHSQSHHNPLSRTSNSTPTNSFLMTSSKPR
Sbjct  11737  ILPSKLKLRTHSQSHHNPLSRTSNSTPTNSFLMTSSKPR  11853



>Homo sapiens chromosome 5, GRCh38.p13 Primary Assembly
Sequence ID: NC_000005.10 Length: 181538259
Range 1: 134926946 to 134927239

Score:132 bits(331), Expect:3e-35,
Method:Compositional matrix adjust.,
Identities:87/98(89%), Positives:91/98(92%), Gaps:0/98(0%)

Query  1          MRHNYNKLHLPTTNRPKIAHCMLFnqphsprsnshshpnpLKLHRRSHSHNRPRAYILIT  60
                  +RHNYNKLHLP TNRPKIAHC+LFNQPHSPRSNSH HPNPLKLHRRSHSH+RPR YILIT
Sbjct  134927239  VRHNYNKLHLPATNRPKIAHCILFNQPHSPRSNSHPHPNPLKLHRRSHSHSRPRTYILIT  134927060

Query  61         ILPSKLKLRTHSQSHHNPLSRTSNSTPTNSFLMTSSKP  98
                  ILPSKLKLRT+ QSHHN LSRTSN TPTNS LMTSSKP
Sbjct  134927059  ILPSKLKLRTYPQSHHNSLSRTSNPTPTNSLLMTSSKP  134926946



>Homo sapiens chromosome 2, GRCh38.p13 Primary Assembly
Sequence ID: NC_000002.12 Length: 242193529
Range 1: 120214413 to 120214703

Score:74.7 bits(182), Expect:3e-15,
Method:Compositional matrix adjust.,
Identities:49/97(51%), Positives:68/97(70%), Gaps:0/97(0%)

Query  1          MRHNYNKLHLPTTNRPKIAHCMLFnqphsprsnshshpnpLKLHRRSHSHNRPRAYILIT  60
                  MR+ Y+KL+L TTN+ KI +C+L ++P+S   +S+ HP PLKL+R SH +N PR Y ++
Sbjct  120214413  MRNGYDKLYLSTTNQSKITYCLLLHKPYSTCYHSYPHPKPLKLYRCSHPYNCPRTYFILI  120214592

Query  61         ILPSKLKLRTHSQSHHNPLSRTSNSTPTNSFLMTSSK  97
                  ILPSK +LR +   +H    R SN+  TNS LMTSSK
Sbjct  120214593  ILPSKFELRANPYPNHITYPRPSNTASTNSLLMTSSK  120214703
```

Figure S4. Comparison of MTALTND4 peptide encoded in mitochondrial DNA (mtDNA) and hypothetically in nuclear DNA that have been transferred from mtDNA (NUMT) through evolution. Only sequences with >50% identities and complete without stop codons are shown.

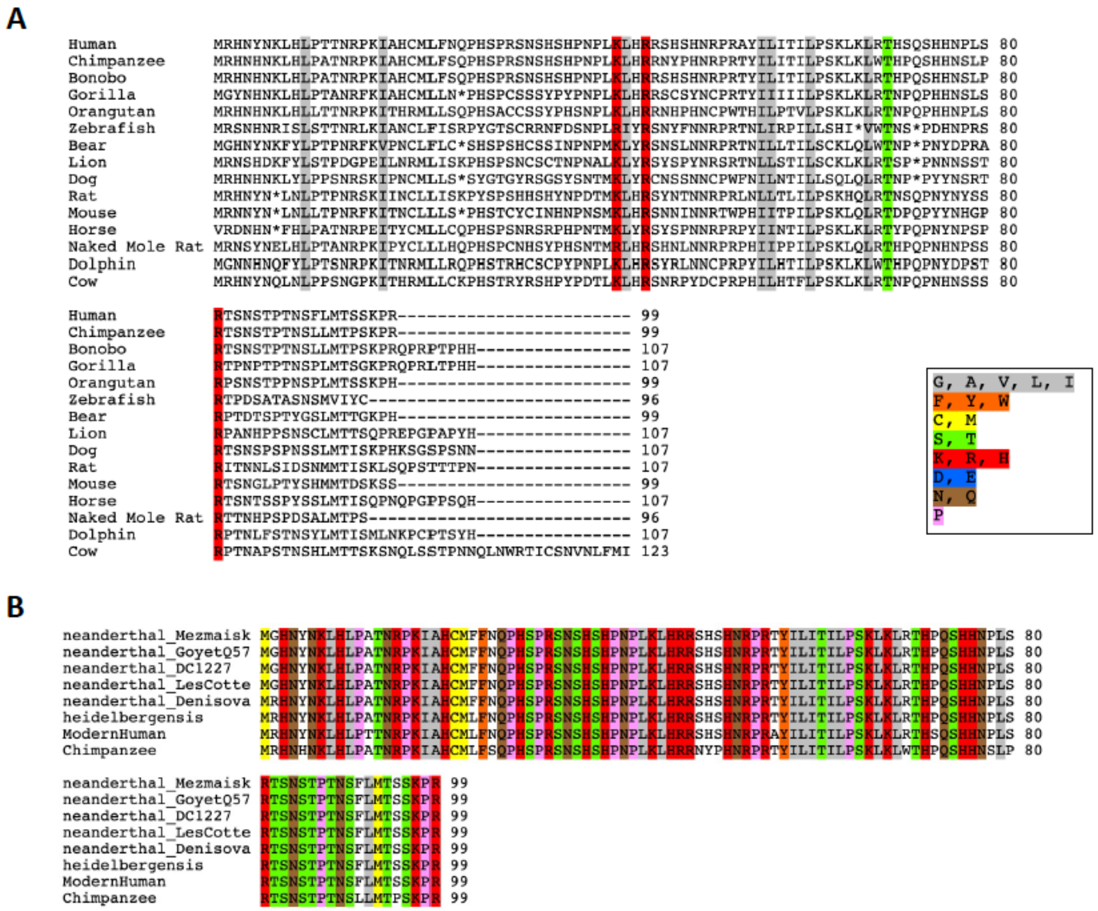

Figure S5. Multiple MTALTND4 peptide sequence alignments. A. In 15 mammal species: human (*Homo sapiens*), chimpanzee (*Pan troglodytes*), bonobo (*Pan paniscus*), gorilla (*Gorilla gorilla*), orangutan (*Pongo pygmaeus*), mouse (*Mus musculus*), rat (*Rattus norvegicus*), naked mole rat (*Heterocephalus glaber*), dog (*Canis lupus familiaris*), cow (*Bos taurus*), zebrafish (*Danio rerio*), lion (*Panthera leo*), bear (*Ursus arctos*), horse (*Equus caballus*), dolphin (*Tursiops truncatus*). **B.** In the genus *Homo* (including chimpanzee): *Homo heidelbergensis* (Sima de los Huesos; KF683087.1); *Homo sapiens neanderthalensis* (Denisova 15; MK033602.1); *Homo sapiens neanderthalensis* (Les cottes; MG025536.1); *Homo sapiens neanderthalensis* (Mezmaiskaya 2; MG025537.1); *Homo sapiens neanderthalensis* (DC1227; KU131206.2); *Homo sapiens neanderthalensis* (GoyetQ57-2; KX198088.1); modern human (*Homo sapiens*), chimpanzee (*Pan troglodytes*). *Indicates a stop codon.

MTALTND4: MRHNYNKLHLPTTNRPKIAHCMLFNQPHSPRSNSHSHPNPLKLHRRSH
SHNRPR<u>**AYILITILPSK**</u>LKLRTHSQSHHNPLSR<u>**TSNSTPTNSFLMTSSKPR**</u>

<u>**AYILITILPSK**</u>

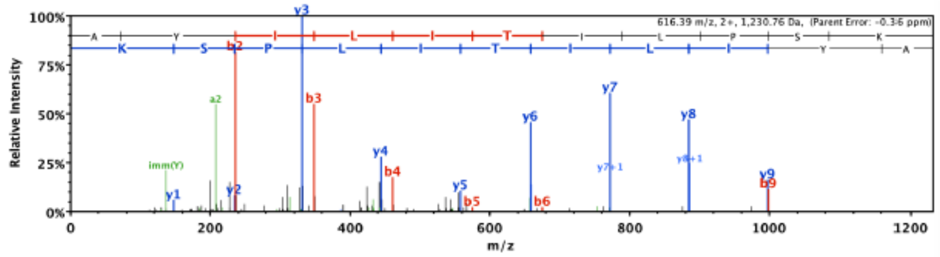

<u>**TSNSTPTNSFLMTSSKPR**</u>

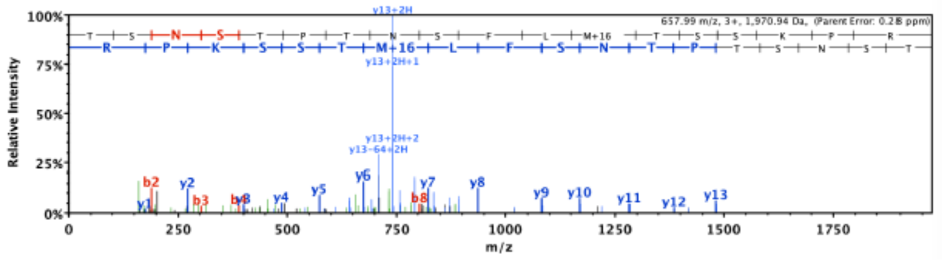

Figure S6. Endogenous MTALTND4 detected in HeLa cells by mass spectrometry. Unique mass spectrometry MTALTND4-derived peptides detected in HeLa cells lysates. Probabilities = 100%.

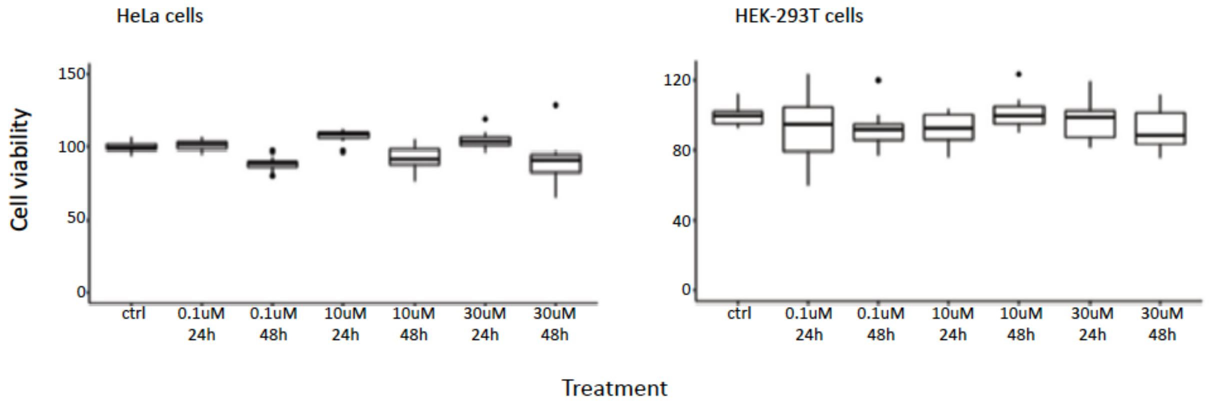

Figure S7. Effect of MTALTND4 on cell viability. HeLa and HEK-293T cells were cultured in low glucose DMEM with 0.1 µM, 10 µM or 30 µM MTALTND4 peptide or water (control) for 24h or 48h and assessed for cell viability using the alamarBlue assay (n=3 for each cell line).

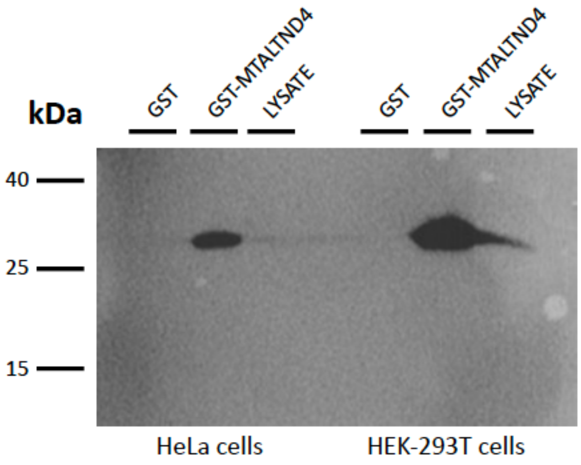

Figure S8. GST pull-down assay indicating that MTALTND4 and Complement component 1 Q interact. Western blot probed with anti-C1qbp antibodies. Lane 1: pull-down products from GST bound glutathione beads (HeLa cells). Lane 2: pull-down products from GST-MTALTND4–bound glutathione beads (HeLa cells). Lane 3: HeLa cells lysate. Lane 4: pull-down products from GST bound glutathione beads (HEK-293T cells). Lane 2: pull-down products from GST-MTALTND4–bound glutathione beads (HEK-293T cells). Lane 3: HEK-293T cells lysate.

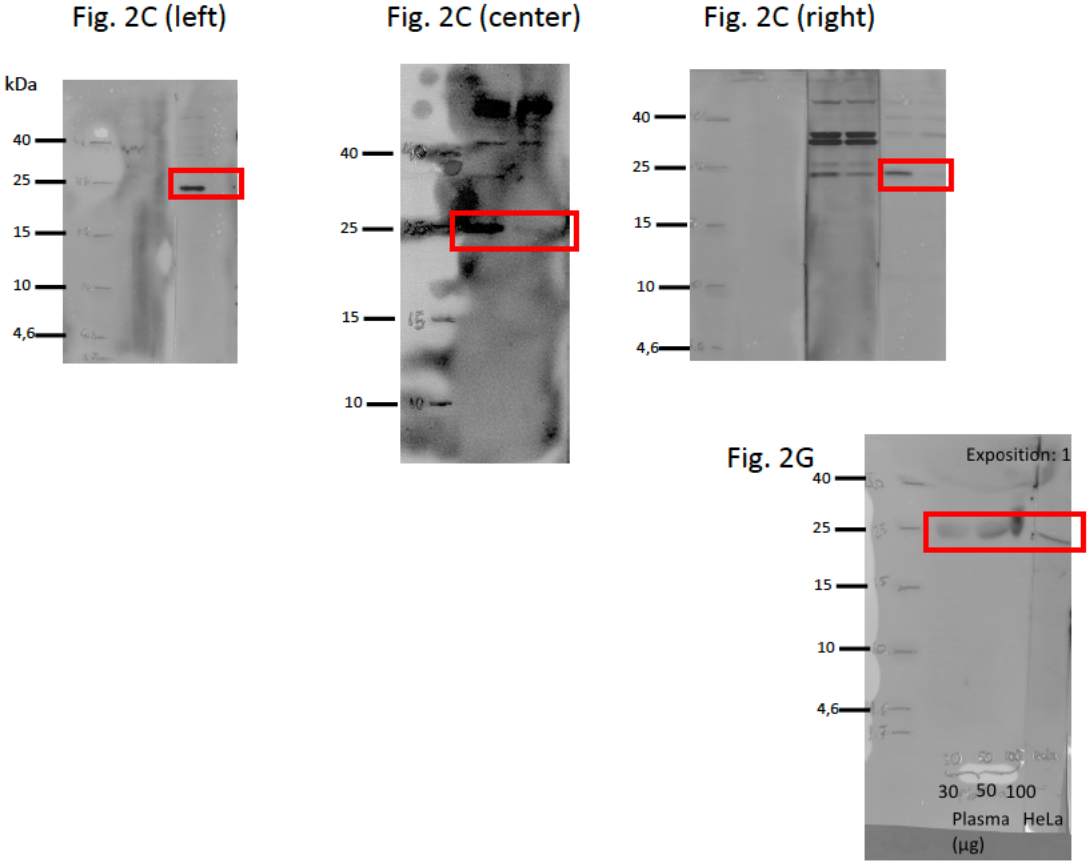

Figure S9. Original uncropped blots for MTALTND4.
