# Supplementary material for: A small protein coded within the mitochondrial canonical gene nd4 regulates mitochondrial bioenergetics
Source: BMC Biol. 2023 May 18;21:111. doi: 10.1186/s12915-023-01609-y (PMC10193809; doi:10.1186/s12915-023-01609-y)
Supplement: Supplementary file 3 — Additional file 3: Supplementary Text 1. Mitochondrial genetic code. Supplementary Text 2. Observed and expected molecular weights on SDS-PAGE. Supplementary Text 3. Conservation of MTALTND4. Supplementary Text 4. Transcription and translation of MTALTND4. [file 12915_2023_1609_MOESM3_ESM.pdf]

Supplementary Text 1

Mitochondrial genetic code

The codons AGA and AGG, which conventionally code for arginine, were thought to be recognized as termination codons in vertebrate mitochondria because of the absence of a mtDNA-encoded $tRNA^{ARG}$ for these triplet codons, but it has now been shown that this is not the case, at least in humans [27,28]. The only two human mitochondrial genes with AGA and AGG as putative terminators are *cox1* and *nad6* and it has been demonstrated that their two mRNAs would rather terminate at a UAG stop codon created because of a −1 frameshift of the mitoribosome [27,28]. If this was also the case for *mtaltnd4*, the translated peptide would be 59 amino-acids long with an expected size of 6.9 kDa. Alternatively, with AGA and AGG coding and a translation occurring either within the cytoplasm, as suggested for MOTS-c because of its tandem ATGAGG start and "putative stop" codons using the typical mitochondrial genetic code [16], or in mitochondria, as suggested for some of the SHLPs that contain putative AGA and AGG coding triplets [17], MTALTND4 consists of 99 amino acids with an expected size of 11.5 kDa and no predicted transmembrane helix. In accordance with some of the SHLPs [17], our results imply a possible import of the cytosolic $tRNA^{AGR}$ into the mitochondria, an innate ability of mammalian mitochondria [29,30]. This scenario would also parsimoniously explain the previously reported intra-mitochondrial localization of endogenous MOTS-c [16,20], suggesting that the peptide is translated in the mitochondria rather than imported after the translation in the cytoplasm of its exported mRNA. Further experiments, such as a treatment of cultured cells with chloramphenicol, will be needed to test this hypothesis for MOTS-c.

Supplementary Text 2

Observed and expected molecular weights on SDS-PAGE

It is not uncommon to observe anomalous mobility during denaturing and reducing electrophoresis. For proteins that appear bigger than expected on SDS-PAGE, such as MTALND4 (expected size of ~12 kDa and observed size of ~24 kDa), this can be the result of post-transcriptional or post-translational modifications, dimerization or multimerization.

Post-transcriptional modifications such as RNA editing can cause a transcript to have a different sequence to that of the DNA template and give rise to a gene encoding a longer protein. However, since *mtaltnd4* is hidden in the *nd4* gene, mitochondrial RNA editing seems improbable unless *mtaltnd4* would possess its own mRNA transcript (or the edition would change only *mtaltnd4* and not *nd4*). Yet, to our knowledge, ultra-deep RNA-sequencing revealed that only three sites harbor RNA–DNA differences in the human mtDNA and they are situated in the 16S rRNA, the D-loop and the *nd5* gene [31].

Post-translational modifications such as glycosylation and phosphorylation can also cause a protein to appear bigger on SDS-PAGE. We tried different techniques (i.e. using a deglycosylation kit and a lambda protein phosphatase) to verify if these predicted modifications (Additional file 2: Fig. S2) were responsible for the observed MTALTND4 apparent mass, and they were not. However, other modifications that we did not verify such as acetylation, methylation, myristoylation, sumoylation and ubiquitination can all increase the molecular weight of a protein. We checked for known motifs for these post-translational modifications and did not detect any with probabilities >75%. Two methylation sites, one acetylation site and one

ubiquitination site with 50-65% probabilities were detected (data not shown). It could be worth exploring the possibility that these modifications occur on MTALTND4.

Another possibility causing a protein to appear bigger than expected on SDS-PAGE is the lack of sufficient SDS binding resulting in insufficient 'electromotive force' and consequently lowered electrophoretic mobility [32]. This can occur because of excess negative charges on the protein, or clusters of negative charges [32]. Highly basic proteins with an excess of positive charges can also have a higher apparent molecular weight due to a reduced charge-to-mass ratio, resulting in a slower migration in SDS-PAGE [33]. Anomalous mobility of proteins on SDS-PAGE are usually seen if the ratio of the numbers of negatively-charged and positively-charged residues is equal to or greater than the value of 1.50, and this can sometimes almost double the expected molecular weight of a protein [32]. This is interesting since MTALTND4 contains 28 basic residues and no acidic residues (isoelectric point = 12.3).

Since the apparent mobility of mtaltnd4 is exactly twice its predicted MM, we considered the possibility of dimerization. To address this issue, we first used strong reducing conditions known to dissociate unusually stable mercaptoethanol-resistant dimers [80]. Specifically, we exposed our samples to different concentrations of ß-mercaptoethanol (0%, 5% and 10%) and different heating times (5min, 30min and 3 hours) (Additional file 2: Fig. S3C). The absence of 'metamorphosis' of MTALTND4-24kDa into 12kDa suggested that MTALTND4-24kDa was not a dimer of 12kDa-MTALTND4 monomers. However, some dimers may still remain 'undisruptable' by heat, SDS, strong denaturants, and/or reducing agents [34]. We thus tried another approach [35] to assess if MTALTND4 dimerizes and showed that purified synthetic MTALTND4 of 12kDa can be cross-linked into a higher molecular complex of 24kDa (Additional file 2: Fig. S3D - using fully denaturing SDS/PAGE Western conditions), suggesting that MTALTND4 can self-associate to form homodimers.

Supplementary Text 3

Conservation of MTALTND4

MTALTND4 is better conserved in primates (Fig. 2E) than in mammals in general (Additional file 2: Fig. S5). One could expect that an altORF of great importance to mitochondrial/cellular function would be highly conserved in animals. However, mitochondria-derived micropeptides such as Humanin, MOTS-c and SHLPs that appear to modulate mitochondrial and cellular biology, as well as global physiology, are not well conserved among mammals or vertebrates [16,37,38]. For example, pseudogenization of the Humanin gene is common in the mtDNA of many vertebrates [37]. Also, several subunits of the OXPHOS complexes encoded by the nucleus, although having key roles in the assembly or activity of the complex or in mitochondrial respiration, are not found in all animal species [39,40].

The process leading to the creation of new proteins by mutations within a coding sequence that lead to the expression of a novel protein in another reading frame is called "overprinting" [41,42]. For mitochondria, with their reduced genome size and constraints on protein birth by duplication or fusion of existing coding sequences, overprinting may be more frequent [41,42].

Supplementary Text 4

Transcription and translation of MTALTND4

The discovery of a new protein ideally includes observations at both the transcription and protein levels. However, the detection of full-length mRNA transcripts or proteins is not an easy task. The vast majority of studies do not report full length mRNA or full length proteins. To our knowledge, none of the previous studies on mitochondria-derived micropeptides have provided full-length mRNA transcripts and only one micropeptide has been partially detected using mass spectrometry (i.e. SHMOOSE) [16,17,19]. This is probably because full-length mitochondrial mRNA transcripts are difficult to study since mRNA species differ from one another. For example, Temperley et al. [27] wrote: *"the 'generic' mitochondrial mRNA is perceived as having (i) an arrangement within the polycistronic unit that permits its liberation following mt-tRNA processing, (ii) no 5′ cap structure or introns, (iii) essentially no untranslated regions, and (iv) a poly(A) tail of approximately fifty nucleotides that is required in part to complete the termination codon. Closer inspection reveals that only two molecules fit this pattern, MT-ND2 and MT-ND3. Although mt-mRNAs as a group do share particular features that mark them as different from cytosolic mRNAs, they also exhibit significant variability as a class".*

As suggested in previous studies, this heterogeneity may be explained by the efficiency of mt-RNA processing and polyadenylation patterns that differ according to cell and tissue types [27,44,45]. 'Unexpected RNA species', such as transcripts with inappropriate lengths (i.e. with non-canonical 5' and 3' ends, making them shorter or longer than expected), transcripts with internal polyadenylation and transcripts without polyA tails, have been reported in many studies [27,45,46,47,48,49], and it remains uncertain whether some of these RNA species represent true precursors or products of imperfect cleavage or degradation. According to these studies, it seems that every transcript originating from the human mitochondrial genome, including the intergenic regions of the polycistronic transcripts, can be polyadenylated by stable and/or internal poly(A) tails, or be non-polyadenylated, making it very difficult to identify 'true' coding transcripts [45,46,47,48,49]. It is worth mentioning that correctly processed mitochondrial transcripts missing polyadenylation can generate fully functional mature polypeptides [49].

In vertebrates, *nd4*, together with *nd4L*, are transcribed as one bicistronic mRNA, and are therefore localized together. This bicistronic mRNA initially codes for ND4L and the reading frames for the two proteins overlap (the AUG start codon of *nd4* is 1 nt upstream of the stop codon for *nd4L*). Regarding the *nd4* mRNA, all scenarios have been observed, i.e. an 'expected' transcript possessing a 5'UTR (i.e. the out of frame sequence encoding the upstream ORF *nd4L*), followed by the complete *nd4* ORF and a polyA tail of about 50 nt [27], but also 'unexpected' shorter or longer transcripts, with internal polyadenylation or without polyA tails [46,47,48,49]. Mitoribosome stalling events on the *nd4*-encoded mRNA have also been reported, suggesting the possibility of intra-*nd4* translation initiation [50]. Altogether, these observations suggest the presence of putative mRNA species corresponding to specific regions of the *nd4* transcript that could encode shorter alternative proteins.

How two overlapping mitochondrial ORFs are translated from the same transcript? As mentioned above, the AUG start codon of *nd4* is 1 nt upstream of the stop codon for *nd4L*, and hence, there is a 5'UTR preceding the ORF encoding ND4, which is the out of frame sequence encoding the upstream *nd4L* ORF, and in reciprocal fashion, the downstream *nd4* ORF constitutes the 3′UTR of the *nd4L* ORF of the bicistronic RNA unit [27]. The second mitochondrial bicistronic RNA, which initially codes for ATP8 and then ATP6, is different of the *nd4L/nd4* mRNA in that the reading frames for these proteins overlap by 40 nt. This is why Jones et al. [51] speculated that

the initiation of translation at the *atp6* start site would occur independently than the initiation at the *atp8* start site rather than by slippage of the ribosome from the *atp8* stop codon back to the *atp6* start codon, as speculated for *nd4L/nd4*. Indeed, ribosome slippage is one mechanism that has been proposed for the translation of mitochondrial bicistronic transcripts. This scenario was recently put forward in a study that showed that translation of downstream *nd4* and *atp6* ORFs depends on the translation of the respectively upstream-localized *nd4L* and *atp8* ORFs, suggesting a single ribosome-mRNA engagement. It is woth noting that these authors did not rule out multiple mitoribosome engagements [52]. Multiple mitoribosomes mRNA engagements was also recently supported by another study in which mitoribosome density measures across different cell lines indicated higher levels for the downstream ORFs *nd4* and *atp6* compared to the upstream *nd4L* and *atp8* ORFs [49].

Overall, the mechanisms for the translation of bicistronic mitochondrial transcripts remain poorly understood. Similarly, the mechanisms for the translation of inner altORFs such as *mtaltnd4* remain an open question. *Mtaltnd4* can be considered as an internal altORF in the *nd4* mRNA and/or an ORF in the 3'UTR of the *nd4L* mRNA. In the first case, we speculate that an independent initiation event is the most parsimonious hypothesis for the initiation of translation of MTALTND4. Mitoribosome slippage would require a move backward of several hundred nucleotides after the completed translation of ND4. For example, a proportion of scanning complexes could fail to initiate at the codon start of *nd4* leading to translation of downstream ORFs. This is known as leaky scanning, a widespread phenomenon in eukaryotic cells [53]. In the second case, where *mtalnd4* is considered as an ORF in the 3'UTR of the *nd4L* mRNA, the translation of MTALTND4 could be initiated *de novo* with an independent ribosome association event or be reinitiated following the complete translation of ND4L, with the small ribosomal subunit remaining attached and continuing to scan the mRNA, a mechanism known as termination-reinitiation [54]. It is worth mentioning that a recent study uncovered an ORF in the 3'UTR of the mitochondrial gene *nd5* that may be translated, as suggested by its start codon that is retained in the human population and the mitoribosome density across the ORF that has comparable levels to densities found on other OXPHOS ORFs [55]. Finally, alternative mechanisms of translation initiation could involve mRNA *cis*-elements such as internal ribosome entry sites (IRESs), which are structured RNA sequences that facilitate translation initiation that were first described in viral mRNAs but that are now known to function also in nuclear mRNAs [56,57], and possibly in mitochondrial mRNAs [58]. N^6-Deoxyadenosine methylation, or m^{6}A, a prevalent modification in nuclear- and mitochondrial-encoded mRNAs [59], is another mechanism able to promote translation initiation from specific start codons in nuclear mRNAs [56], and possibly in mitochondrial mRNAs [60].

In conclusion, the literature above suggests that the optimal way to demonstrate the existence of a functional noncanonical mitochondrial ORF is to provide proof at the protein level. Although mass spectrometry does not allow the detection of full-length proteins, we were able to detect two unique MTALTND4-derived peptides in fractions immunoprecipitated with the MTALTND4 antibody (Additional file 2: Fig. S6). These peptides sequences covered 30% of the protein sequence. Interestingly, no identical hits were found for these two peptides using BLASTp searches against *Homo sapiens* protein sequences, supporting our conclusion that MTALTND4 is a novel mitochondria-derived protein.
